# Supplementary material for: Thioesters provide a plausible prebiotic path to proto-peptides
Source: Nat Commun. 2022 May 11;13:2569. doi: 10.1038/s41467-022-30191-0 (PMC9095695; doi:10.1038/s41467-022-30191-0)
Supplement: Supplementary file 1 — Supplementary Information [file 41467_2022_30191_MOESM1_ESM.pdf]

Supplementary Information

Thioesters Provide a Plausible Prebiotic Path to Proto-Peptides

Moran Frenkel-Pinter[a,b,c], Marcos Bouza[a,b], Facundo M. Fernández[a,b], Luke J. Leman[a,d], Loren Dean Williams[a,b], Nicholas V. Hud[a,b*], and Aikomari Guzman-Martinez[a,e*]

[a] NSF-NASA Center for Chemical Evolution, Georgia Institute of Technology, Atlanta, GA 30332 (USA)

[b] School of Chemistry & Biochemistry, Georgia Institute of Technology, Atlanta, GA 30332 (USA)

[c] Institute of Chemistry, The Hebrew University of Jerusalem, Israel 91904

[d] Department of Chemistry, The Scripps Research Institute, La Jolla, CA 92037 (USA)

[e] Department of Chemistry, University of Puerto Rico, Mayagüez, Mayagüez, PR 00681 (USA)

*Corresponding authors

*Corresponding Authors:

Prof. Aikomari Guzman-Martinez
Department of Chemistry
University of Puerto Rico, Mayagüez
Mayagüez, PR 00681
Ph: (+1) (787) 832-4040
Fax: (+1) (787) 265-3849
aikomari.guzman@upr.edu

Prof. Nicholas V. Hud
School of Chemistry and Biochemistry
Georgia Institute of Technology
315 Ferst Drive NW
Atlanta, GA 30332-0400
Ph: (+1) (404) 385-1162
Fax: (+1) (404) 894-2295
hud@chemistry.gatech.edu

Table of Contents

Supplementary Methods 5
Materials 5
Synthesis of thioglycolic-L-alanine standard 5
Synthesis of thioglycolic-L-alanine-L-Alanine standard 9
Synthesis of L-alanine-thioglycolic acid standard 12
Synthesis of thioglycolic acid-L-alanine-L-Alanine-L-Alanine standard 14
Synthesis of (S)-3-methylthiazine-2,5-dione standard 14
Crystal structure determination of (S)-3-methylthiazine-2,5-dione 17
Supplementary Figures 19
Supplementary Figure 1. C18-HPLC analysis shows greater yield of thiodepsipeptides following dry-down reactions of tg and Ala when tg is in excess. 19
Supplementary Figure 2. C18-HPLC analysis of thiodepsipeptide formation following dry-down reactions of tg and Ala at 1:1 molar ratio at varying amounts. 20
Supplementary Figure 3. Some of the possible products of a dry-down reaction of tg and Ala. 21
Supplementary Figure 4. Minimal oxidation occurs upon dry-down reactions under experimental anoxic conditions. 22
Supplementary Figure 5. High reproducibility of product mixture is observed upon dry-down of mercaptoacid and amino acids. 23
Supplementary Figure 6. Fourier Transform Infrared Spectroscopy (FTIR) shows shifts in the C=O stretch upon dry-down of tg, supportive of thioester formation. 24
Supplementary Figure 7. Validation of the formation of tgA peptide upon dry-down of tg with Ala. 25
Supplementary Figure 8. Validation of the formation of tgAA peptide upon dry-down of tg with Ala. 26
Supplementary Figure 9. Validation of the formation of tgAAA peptide upon dry-down of tg with Ala. 27
Supplementary Figure 10. Validation of the formation of c(tgA) upon dry-down of tg with Ala. 28
Supplementary Figure 11. Quantification of products from dry-down reactions using calibration curves of synthesized standards. 29
Supplementary Figure 12. Two plausible pathways for acyl substitutions for tgAA formation through thioester-amide exchange. 30
Supplementary Figure 13. Confirmation of thioester-containing compounds upon dry-down reactions of tg and Ala. 31

Supplementary Figure 14. Hydrolysis occurs upon incubation of dry-heated reactions in water. 32
Supplementary Figure 15. Kinetics of thiodepsipeptide formation over time. 33
Supplementary Figure 16. Proposed ring-opening polymerization pathways using c(tgA). 34
Supplementary Figure 17. Dry-down reactions of c(tgA) in the presence or absence of tg or alanine. 35
Supplementary Figure 18. Dry-down reactions of Atg in the presence or absence of tg or alanine. 36
Supplementary Figure 19. Dry-down reactions of tgA in the presence or absence of tg or alanine. 37
Supplementary Figure 20. Verification of formation of amide bonds in dry-down reactions of mercaptoacids and amino acids using MS/MS analysis. 38
Supplementary Figure 21. Full spectrum of a fresh, non-dried tg plus Ala mixture. 39
Supplementary Figure 22. Full spectrum of a dried mixture of tg plus Ala mixture. 40
Supplementary Figure 23. ^{1}H-1H COSY NMR spectra of dry-down reactions of tg and Ala. 41
Supplementary Figure 24. Full spectrum of the tgA standard. 42
Supplementary Figure 25. Validation of the formation of tgA peptide upon dry-down of tg with Ala. 43
Supplementary Figure 26. Full spectrum of the tgAA standard. 44
Supplementary Figure 27. Validation of the formation of tgAA peptide upon dry-down of tg with Ala. 45
Supplementary Figure 28. ESI-MS of a dry-down reaction of tg and Gly at a 5:1 molar ratio supports the formation of thiodepsipeptides. 46
Supplementary Figure 29. HPLC of a dry-down reaction of tg and Gly at a 5:1, 1:1 molar ratio or Gly control supports the formation of thiodepsipeptides. 47
Supplementary Figure 30. ESI-MS of a dry-down reaction of tg and L-Phe at a 5:1 molar ratio supports the formation of thiodepsipeptides. 48
Supplementary Figure 31. HPLC of a dry-down reaction of tg and Phe supports the formation of thiodepsipeptides. 49
Supplementary Figure 32. ESI-MS of a dry-down reaction of ta and L-Ala at a 5:1 molar ratio supports the formation of thiodepsipeptides. 50
Supplementary Figure 33. HPLC of a dry-down reaction of ta and L-Ala at a 5:1, 1:1 molar ratio or Ala control supports the formation of thiodepsipeptides. 51
Supplementary Figure 34. ESI-MS of a dry-down reaction of ta and L-Cys at a 5:1 molar ratio supports the formation of thiodepsipeptides. 52
Supplementary Figure 35. HPLC of a dry-down reaction of ta and L-Cys at a 5:1, 1:1 molar ratio or Cys control supports the formation of thiodepsipeptides. 53

Supplementary Figure 36. ^{1}H NMR Spectrum supports the formation of oligomers in dry-down reactions of tg and Cys. 54
Supplementary Figure 37. ^{1}H NMR Spectrum supports the formation of oligomers in dry-down reactions of tg and Phe. 55
Supplementary Figure 38. Formation of thiodepsipeptides in dry-down reactions occurs over a wide range of pH conditions. 56
Supplementary Figure 39. ^{1}H NMR Spectrum of the product mixture from dry-down reactions of tg and Ala with 1 eq of imidazole 57
Supplementary Figure 40. ^{1}H NMR Spectrum of the product mixture from dry-down reactions of tg and Ala with 5 eq of imidazole 58
Supplementary Figure 41. ^{1}H NMR Spectrum of the product mixture from dry-down reactions of tg and Ala with 10 eq of imidazole 59
Supplementary Figure 42. ^{1}H NMR Spectrum of the product mixture from dry-down reactions of tg and Ala with 20 eq of imidazole 60
Supplementary Figure 43. Thiodepsipeptide formation occurs under a range of pH conditions. 61
Supplementary Figure 44. Thiodepsipeptide formation occurs in aqueous solution. 62
Supplementary Figure 45. ^{1}H NMR Spectrum of the product mixture from dry-down reactions of tg and Ala at 25 ˚C. 63
Supplementary Figure 46. ^{1}H NMR Spectrum of the product mixture from dry-down reactions of tg and Ala at 37 ˚C. 64
Supplementary Figure 47. ^{1}H NMR Spectrum of the product mixture from dry-down reactions of tg and Ala at 50 ˚C. 65
Supplementary Figure 48. Imidazole does not catalyze formation of depsipeptides upon drying of glycolic acid with Ala. 66
Supplementary Figure 49. Temperature-dependent formation of depsipeptides under dry-down reactions of glycolic acid and Ala. 67
Supplementary Figure 50. Incubation of glycolic acid or a mixture of glycolic acid and Ala in water does not result in formation of polyesters or depsipeptides. 68
Supplementary Figure 51. Similar conversion of Ala into polymers in dry-down reactions containing mercapto and hydroxy acids. 69
Supplementary Figure 52. Side product formation under oxic conditions. 70
Supplementary Figure 53. Thiodepsipeptide formation during dry-wet cycles. 71
Supplementary References 72

Supplementary Methods

Materials

All chemicals and reagents were obtained from Sigma-Aldrich and used as received.

Synthesis of thioglycolic-L-alanine standard

Preparation of 2-mercaptoacetylamino acids

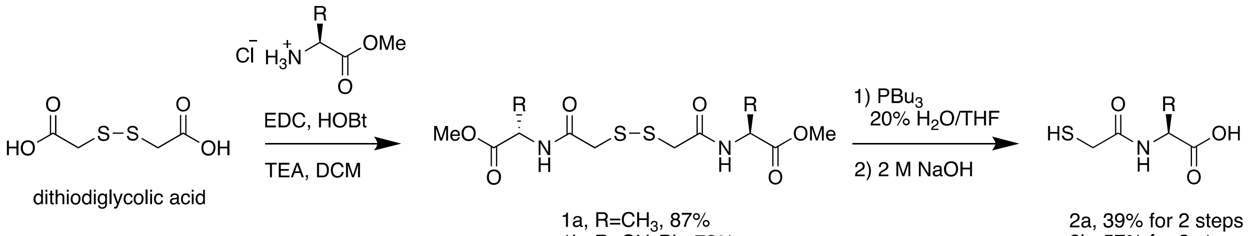

1a, R=CH_3, 87%
1b, R=CH_2Ph, 73%

2a, 39% for 2 steps
2b, 57% for 2 steps

2-Mercaptoacetylamino acids have been synthesized as previously described (1) (see below).

Thioglycolic-L-alanine methyl ester bisamide disulfide, 1a

Dithiodiglycolic acid (0.91 g, 5 mmol, Sigma) was suspended in 40 mL dry DCM. HOBt hydrate (1.84 g, 12 mmol) was added, followed by EDC (2.3 g, 12 mmol) and L-Ala-OMe hydrochloride (1.47 g, 10.5 mmol). Finally, triethylamine (2.09 mL, 15 mmol) was added. The initially cloudy mixture gradually cleared to a reddish solution that stirred at RT overnight. The reaction was diluted with DCM (100 mL) and washed 2x with sat. $NaHCO_3$, 2x with sat. $KHSO_4$, 1x with brine, and dried over $MgSO_4$. TLC (visualized by UV) with 7:3 EtOAc/hexanes showed conversion to the product (Rf of 0.2), and the product mass was confirmed by TLC-ESI-MS (obs'd. 352.8 for M+H, obs'd. 374.7 for M+Na). The solvent was evaporated to yield a yellow oily solid (1.7 g crude weight). The product was purified by flash chromatography using 100 g silica in a 5 cm diameter column with 2% MeOH/DCM as the eluent. After evaporation of the solvent from fractions containing the product, a pale yellow solid was obtained (1.53 g, 87%).

^{1}H NMR (500 MHz, DMSO) δ 8.53-8.51 (d, 2H), 4.33-4.27 (m, 2H), 3.63 (s, 6H), 3.53 (s, 4H), 1.30-1.29 (d, 6H).

^{13}C NMR (126 MHz, DMSO) δ 173.26, 168.28, 52.38, 48.37, 42.23, 17.47.

HRMS calculated [M+H] 353.0841; Found [M+H] 353.0838

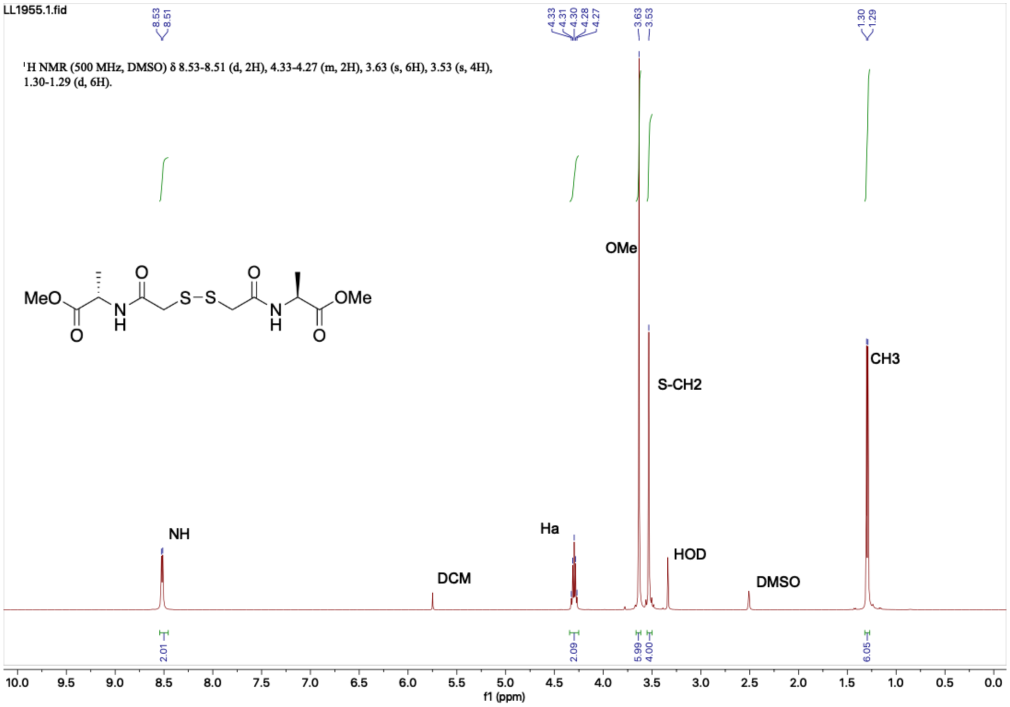

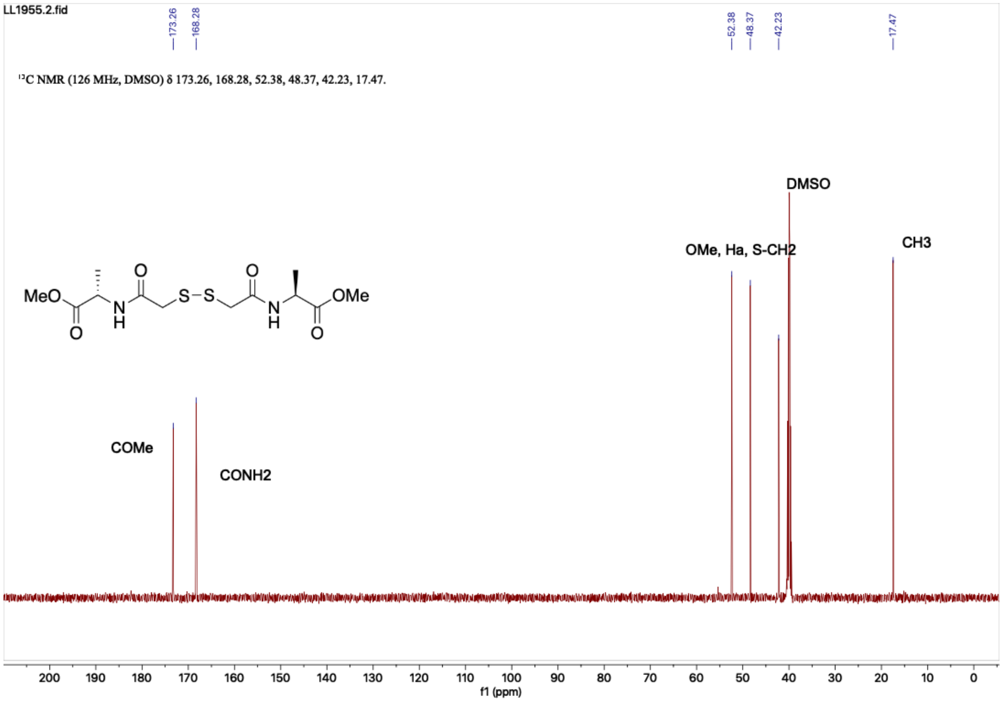

Thioglycolic-L-alanine, 2a

The bisamide disulfide 1a was dissolved in 20% H2O/THF (19 mL). The solution was degassed by bubbling Ar for 10 min, after which Bu3P was added slowly and the mixture stirred at RT for 10 min. TLC (7:3 EtOAc/hexanes, visualized by UV) indicated the disulfide reduction was complete. The mixture was diluted with 7.5 mL ethanol, after which 9.25 mL 2 M NaOH was added. The solution stirred at RT for 1 h. The solvent was evaporated and the residue was taken up in water and extracted 3x with EtOAc. The organic layers were discarded. The aqueous layer was acidified using 1 N HCl to pH 2. The aqueous layer was evaporated to a gummy oil. Purification via preparative HPLC 438 mg (39%) of a white solid.

^{1}H NMR (500 MHz, DMSO) δ 12.59 (br s, 1H), 8.31-8.30 (d, 1H), 4.22-4.16 (m, 1H), 3.18-3.10 (m, 2H), 2.72-2.68 (t, 1H), 1.28-1.26 (d, 3H).

^{13}C NMR (126 MHz, DMSO) δ 174.39, 169.74, 48.23, 27.28, 17.71.

HRMS Calculated [M+H] 164.0381; Found [M+H] 164.0386

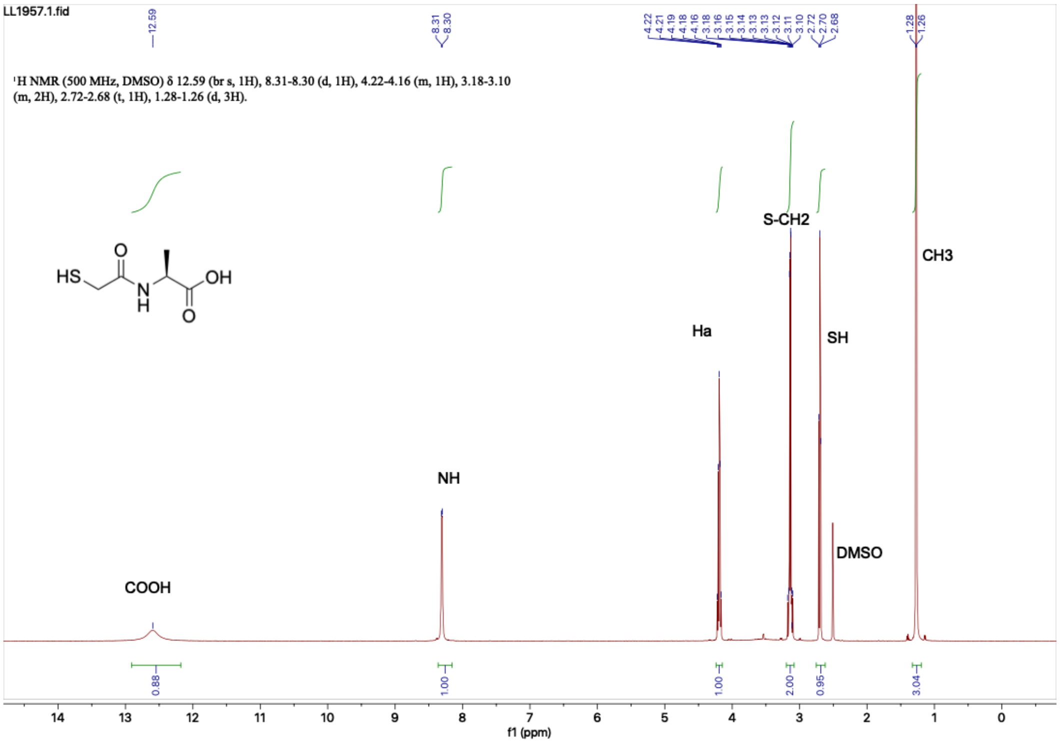

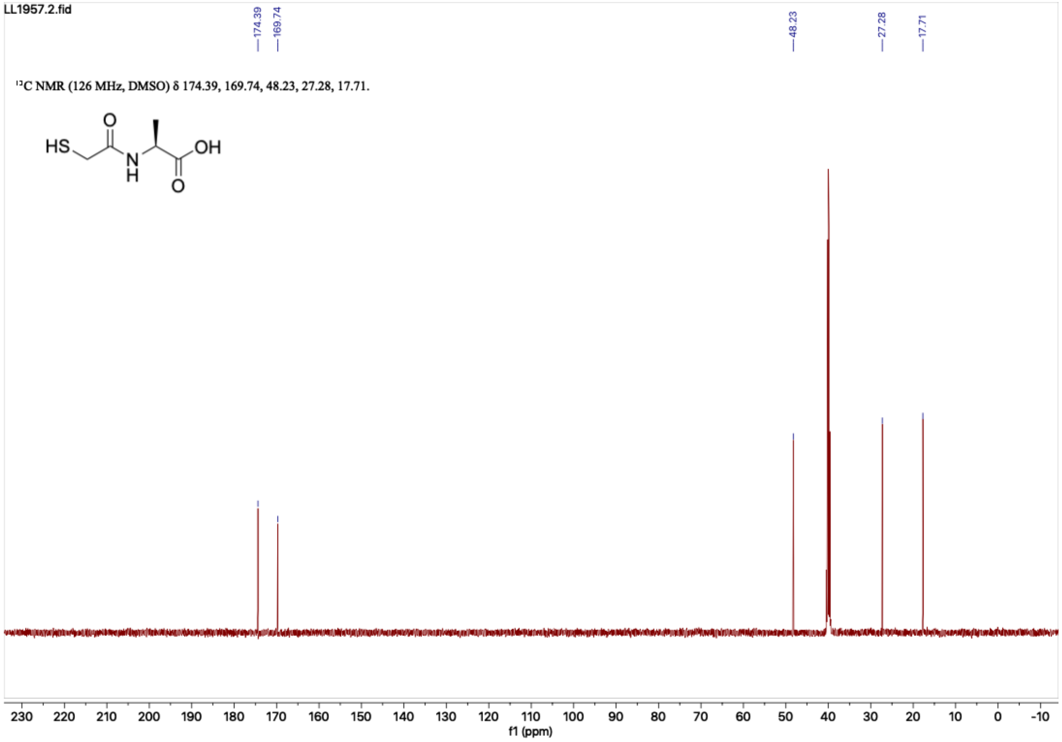
LL1957.2.fid
174.39
169.74
48.23
27.28
17.71
13C NMR (126 MHz, DMSO) δ 174.39, 169.74, 48.23, 27.28, 17.71.
HS
O
N
H
OH
O
230
220
210
200
190
180
170
160
150
140
130
120
110
100
90
80
70
60
50
40
30
20
10
0
-10
f1 (ppm)

Synthesis of thioglycolic-L-alanine-L-Alanine standard

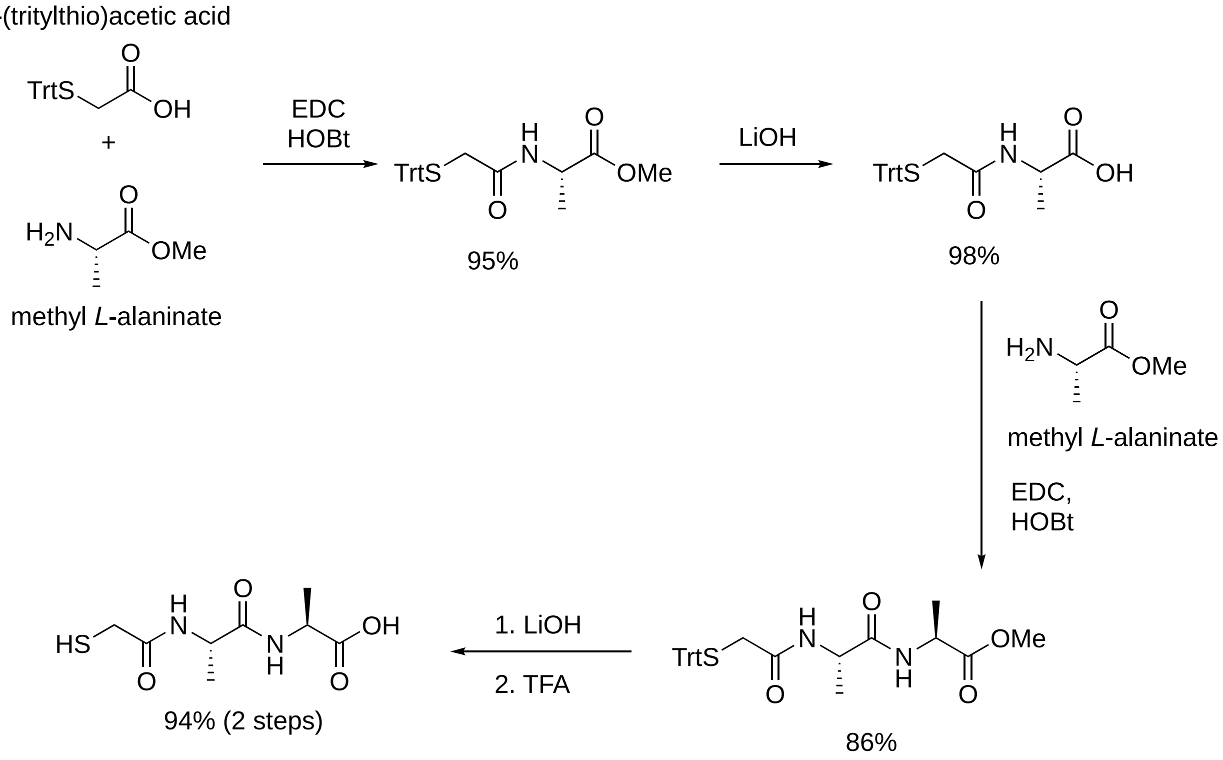

2-(tritylthio)acetic acid (535 mg, 1.6 mmol, Ambeed) and HOBt hydrate (276 mg, 1.8 mmol) were dissolved in DCM (10 mL) and placed in an ice bath. EDC (345 mg, 1.8 mmol) was added, followed by L-Ala-OMe hydrochloride (237 mg, 1.7 mmol). Triethylamine (0.28 mL, 2.0 mmol) was added and the reaction stirred and was allowed to warm to RT overnight. The solvent was removed and the residue was taken up in EtOAc. The organics were washed with saturated $NaHCO_3$ (3x), saturated KHSO4 (3x), and brine. The organic layer was dried over $MgSO_4$, and the solvent was evaporated to yield 0.64 g (95%) of a clear oil. The product was used without further purification. The methyl ester was removed by dissolving the compound (0.64 g, 1.5 mmol) in MeOH (4 mL) and adding a solution of LiOH monohydrate (0.21 g, 5.0 mmol) in water (4 mL). The cloudy mixture was stirred at RT for 1 h, after with HPLC indicated completion of the reaction. The solvent was evaporated and the residue was taken up in EtOAc and 1 N HCl. The organic layer was removed, and the aqueous layer was extracted once more with EtOAc. The combined organic layers were washed with brine and dried over $MgSO_4$. Evaporation of the solvent yielded 0.61 g (98%) of a white foamy solid. Without further purification, the acid thus obtained was coupled to L-Ala-OMe hydrochloride. The acid (200 mg, 0.5 mmol) and HOBt hydrate (87 mg, 0.57 mmol) were dissolved in DCM (5 mL) and placed in an ice bath. EDC (109 mg, 0.57 mmol)

was added, followed by L-Ala-OMe hydrochloride (75 mg, 0.54 mmol). Triethylamine (0.084 mL, 0.6 mmol) was added and the reaction stirred and was allowed to warm to RT overnight. The solvent was removed and the residue was taken up in EtOAc. The organics were washed with saturated $NaHCO_3$ (3x), saturated $KHSO_4$ (3x), and brine. The organic layer was dried over $MgSO_4$, and the solvent was evaporated to yield 0.21 g (86%) of the tritylthioacetic-Ala-Ala-OMe as a white solid. Without further purification, the methyl ester was removed by dissolving the compound (0.21 g, 0.43 mmol) in MeOH (5 mL) and adding a solution of LiOH monohydrate (0.084 g, 2.0 mmol) in water (2 mL). The cloudy mixture was stirred at RT for 1 h, after with HPLC indicated completion of the reaction. The solvent was evaporated and the residue was taken up in EtOAc and 1 N HCl. The organic layer was removed, and the aqueous layer was extracted once more with EtOAc. The combined organic layers were washed with brine and dried over $MgSO_4$. Evaporation of the solvent yielded 0.20 g (quantitative yield) of a white solid. Finally, the trityl group was removed by dissolving the acid thus obtained (0.20 g, 0.42 mmol) in 95:5 TFA/triethylsilane (10 mL) and stirred at RT for 1 h. After this time, the solvent was evaporated, and the product was purified by preparative HPLC. Lyophilization of fractions containing the product yielded 0.09 g of a white solid (92%).

^{1}H NMR (500 MHz, D_2O) δ 4.36- 4.31 (q, 2H), 3.27 (s, 2H), 1.43-1.40 (d, 6H)
^{13}C NMR (126 MHz, D_2O) δ 176.37, 174.58, 173.44, 49.67, 48.59, 26.79, 16.42, 16.02
HRMS Calculated [M+H] 235.0747; Found [M+H] 235.0743

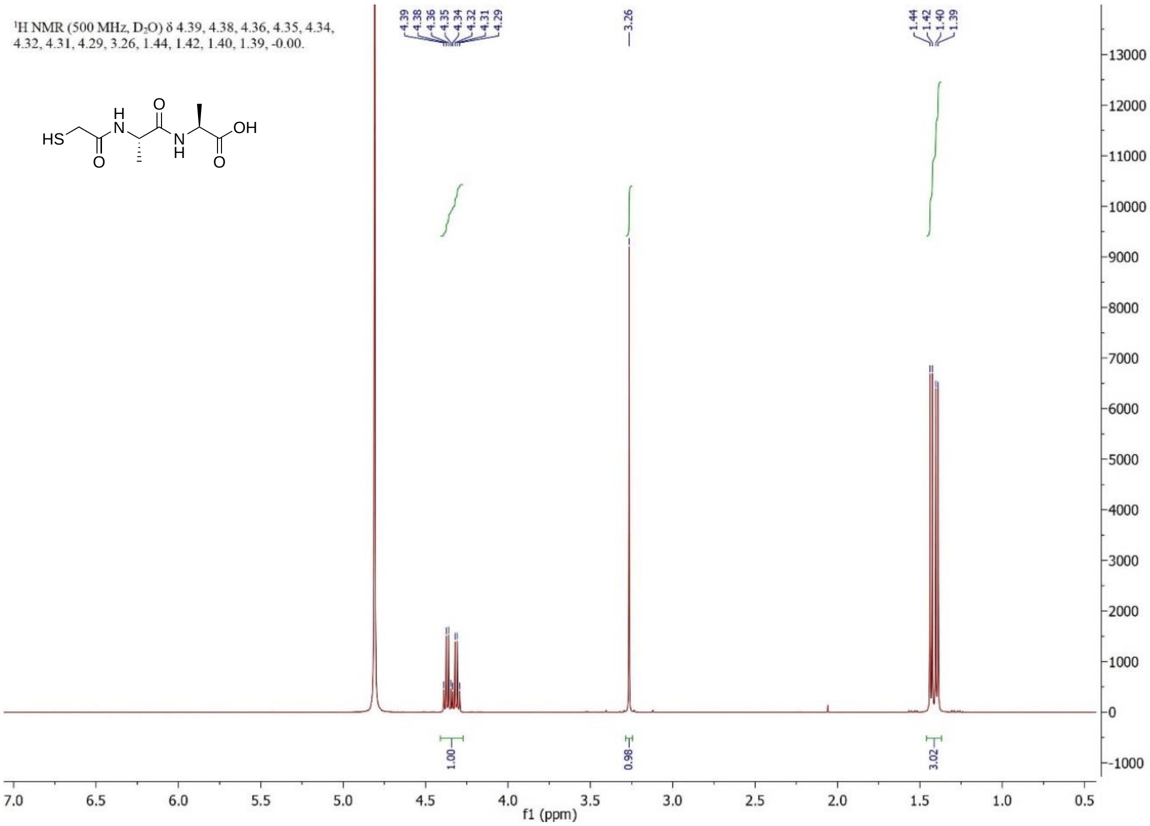
1H NMR (500 MHz, D2O) δ 4.39, 4.38, 4.36, 4.35, 4.34, 4.32, 4.31, 4.29, 3.26, 1.44, 1.42, 1.40, 1.39, -0.00.
HS
N
H
O
O
N
H
OH
O
f1 (ppm)

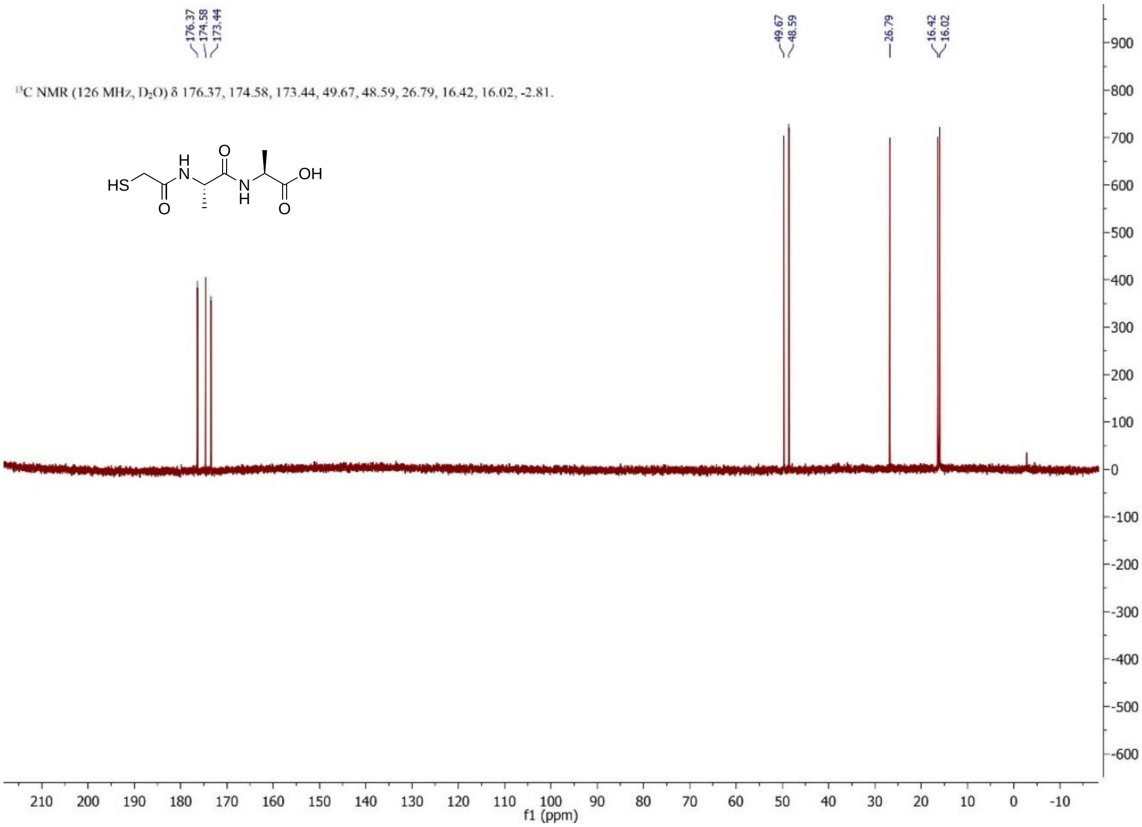
13C NMR (126 MHz, D2O) δ 176.37, 174.58, 173.44, 49.67, 48.59, 26.79, 16.42, 16.02, -2.81.
HS
N
H
O
O
N
H
OH
O
f1 (ppm)

Synthesis of L-alanine-thioglycolic acid standard

L-Ala-thioglycolic acid trifluoroacetate.

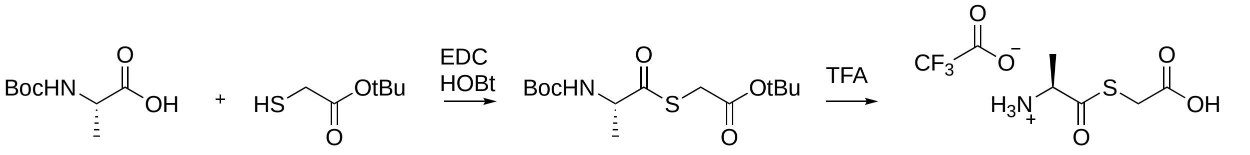

Boc-L-Ala (378 mg, 2.0 mmol) and HOBt hydrate (367 mg, 2.4 mmol) were dissolved in DCM (10 mL) and placed in an ice bath. EDC (460 mg, 2.4 mmol) was added, followed by tBu-2-sulfanylacetate (341 mg, 2.3 mmol, Enamine). The reaction stirred and was allowed to warm to RT overnight. The solvent was removed and the residue was taken up in EtOAc. The organics were washed with saturated NaHCO3 (3x), saturated KHSO4 (3x), and brine. The organic layer was dried over MgSO4, and the solvent was evaporated to yield 0.61 g of a clear oil. Without further purification, the protected thioester was dissolved in 95:5 TFA/triethylsilane (10 mL) and stirred at RT for 1 h. The solvent was evaporated, and the product was purified by preparative HPLC. Lyophilization of fractions containing the product yielded 0.48 g of a clear oil (87% over 2 steps).

^{1}H NMR (500 MHz, D_2O) δ 4.49- 4.43 (q, 1H), 3.86 (s, 2H), 1.65-1.63 (d, 3H)

^{13}C NMR (126 MHz, D_2O) δ 198.02, 172.62, 54.82, 31.66, 16.32

HRMS Calculated [M+H] 164.0381; Found [M+H] 164.0375

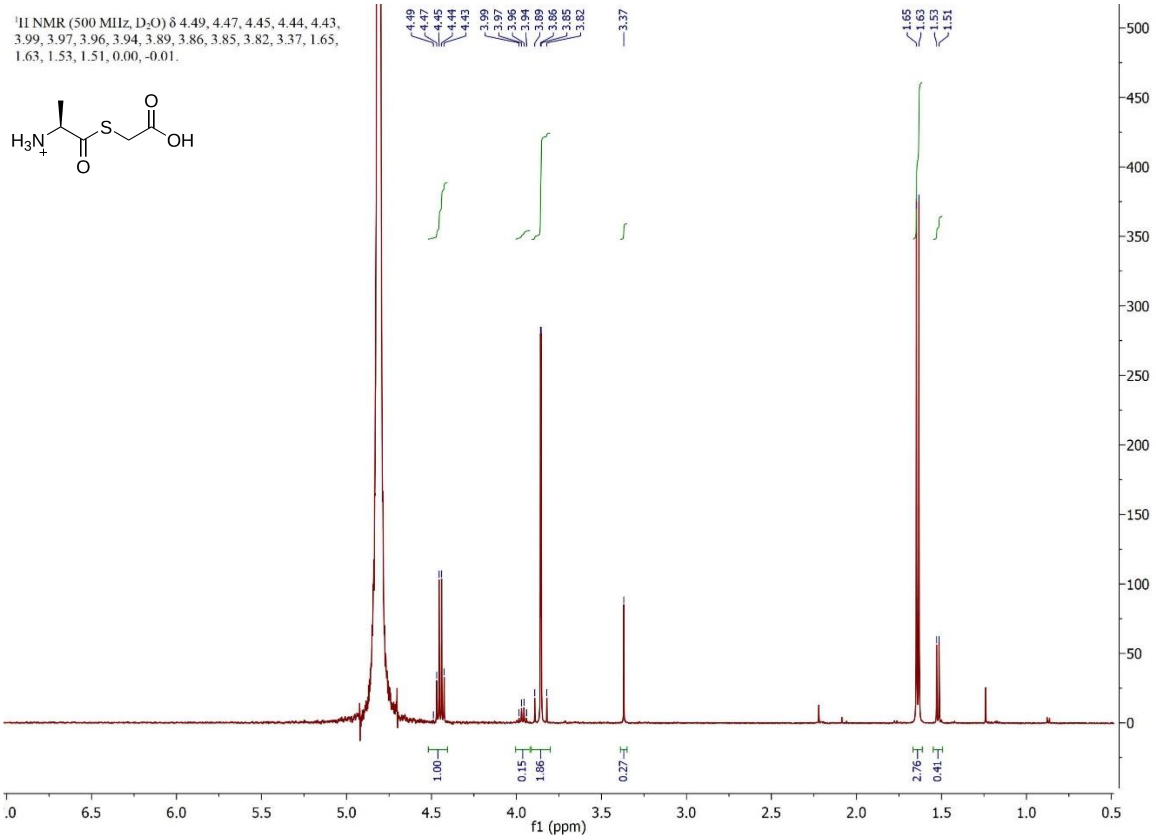
^{1}H NMR (500 MHz, D_2O) δ 4.49, 4.47, 4.45, 4.44, 4.43, 3.99, 3.97, 3.96, 3.94, 3.89, 3.86, 3.85, 3.82, 3.37, 1.65, 1.63, 1.53, 1.51, 0.00, -0.01.
H_3N^+
S
O
OH
1.00
0.15
1.86
0.27
2.76
0.41
f1 (ppm)

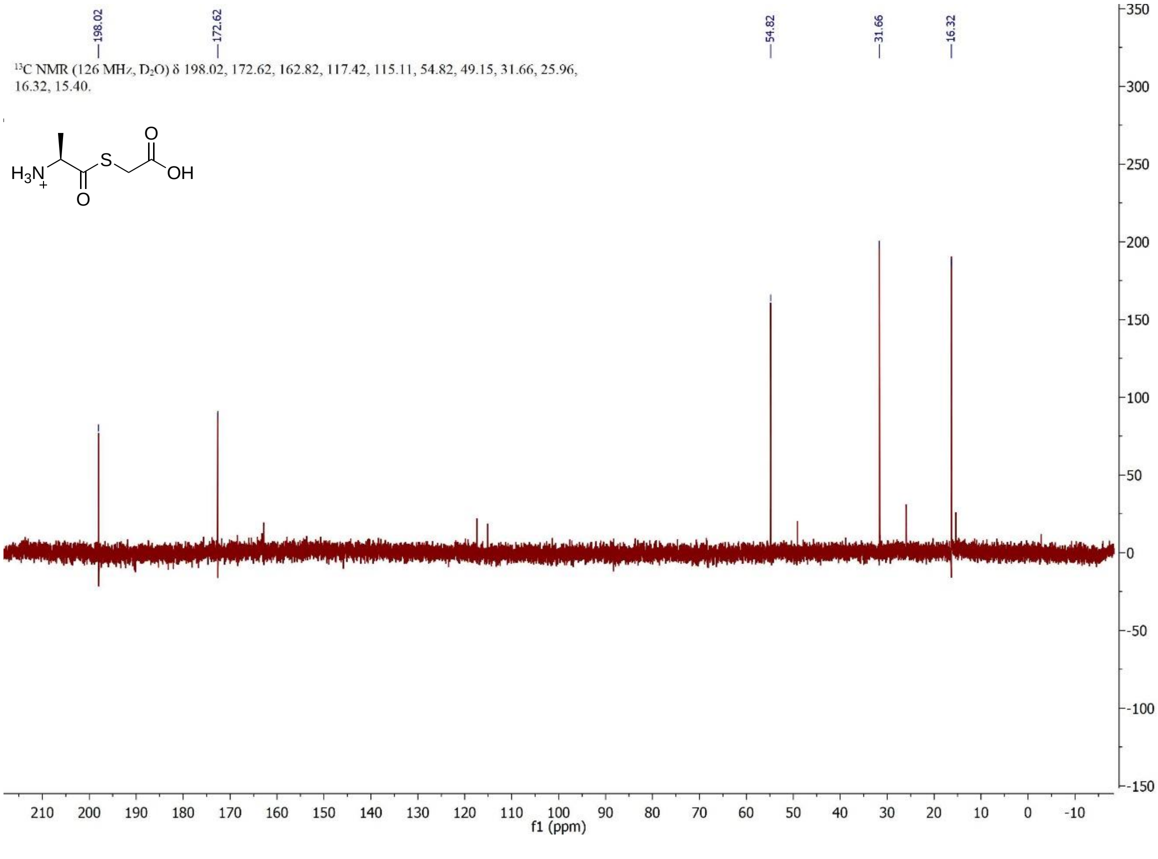
^{13}C NMR (126 MHz, D_2O) δ 198.02, 172.62, 162.82, 117.42, 115.11, 54.82, 49.15, 31.66, 25.96, 16.32, 15.40.
198.02
172.62
54.82
31.66
16.32
H_3N^+
S
O
OH
f1 (ppm)

Synthesis of thioglycolic acid-L-alanine-L-Alanine-L-Alanine standard

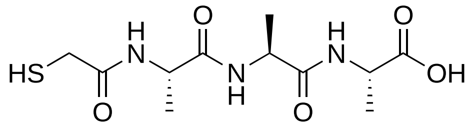

tg-(Ala)$_3$

The compound was synthesized via standard Fmoc solid-phase peptide synthesis protocols on 0.3 mmol scale using Fmoc-Ala-Wang resin. Coupling reactions were carried out using a five-fold excess of oxyma, diisopropylcarbodiimide, and Fmoc-protected Ala or 2-(tritylthio)acetic acid (Ambeed, CAS # 34914-36-8) for 75 min. Fmoc deprotection steps were carried out by treating the resin with 25% 4-methylpiperidine/DMF twice for 10 min. The peptide was cleaved from the resin using 94:2:2:2 TFA/H_2O/ethanedithiol/triethylsilane for 90 min. The cleavage solution was added dropwise to cold diethyl ether (100 mL), and the crude peptide was obtained by centrifugation. The desired compound was purified by preparative HPLC using a water/acetonitrile/TFA solvent system. Fractions containing the product were lyophilized to yield 67 mg of a white powder (73% yield based on resin loading). Purity was verified by analytical HPLC.

HRMS Calculated [M+H] 306.1124; Found [M+H] 306.1121

Synthesis of (S)-3-methylthiazine-2,5-dione standard

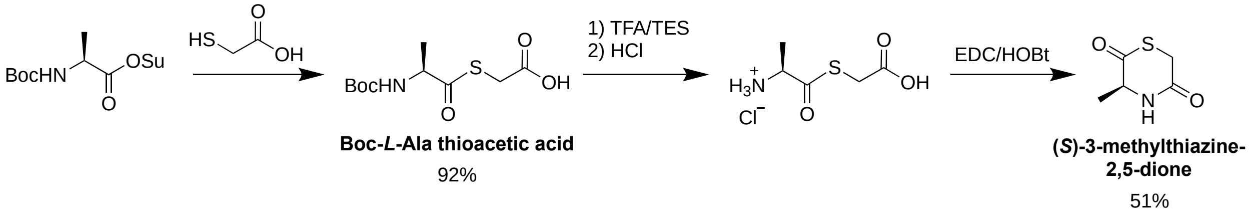

Boc-L-Ala-thioacetic acid

Boc-L-Ala N-hydroxysuccinimide ester (1.225 g, 4.28 mmol, Combi-Blocks) and thioglycolic acid (0.34 mL, 4.88 mmol) were dissolved in dry methylene chloride (30 mL) and chilled to 0 °C. Next, diisopropylethylamine (1.6 mL, 9.16 mmol) was added. After 1 h, the ice bath was removed and the solution was stirred at room temperature for 14 h. The solvent was evaporated and the residue was taken up in EtOAc. The organics were washed 3 times with saturated $KHSO_4$, once with brine, and dried over magnesium sulfate. After filtration and removal of the solvent, a white

solid was obtained (1.04 g, 92% yield). The product was used in the next step without further purification.

^{1}H NMR (500 MHZ, DMSO) δ 12.71 (br s, 1H), 7.71-7.69 (d, 1H), 4.16-4.10 (m, 1H), 3.61 (s, 2H), 1.42 (s, 9H), 1.24-1.22 (d, 3H).
^{13}C NMR (126 MHZ, DMSO) δ 202.81, 170.22, 155.61, 79.13, 56.42, 31.38, 28.68, 17.58.
HRMS Calculated [M+H] 264.0906; Found [M+H] 264.0909

(S)-3-methylthiazine-2,5-dione

Boc-L-Ala-thioacetic acid (800 mg, 3.04 mmol) was dissolved in 95:5 TFA/triethylsilane (10 mL) and allowed to stir at room temperature for 1 h. The volatiles were removed by evaporation. To remove residual water and TFA, the residue was taken up in toluene and evaporated (two times). The resulting white solid was dissolved in 2 N HCL (20 mL) and evaporated (two times) to exchange the TFA salt for the HCl salt. The resulting gummy white solid was lyophilized and used directly in the next step. Methylene chloride (80 mL) was added to the residue and the suspension was chilled to 0 °C. Next were added EDC.HCl (642 mg, 3.35 mmol), HOBt.H_2O (512 mg, 3.35 mmol), and diisopropylethylamine (1.07 mL, 6.14 mmol). The mixture was stirred vigourously for 3 h. The organics were washed 2 times with saturated $NaHCO_3$, 2 times with saturated $KHSO_4$ once with brine, and dried over magnesium sulfate. After filtration and removal of the solvent, a white solid was obtained. The solid was taken up in a minimal volume of hot acetonitrile and allowed to cool slowly to yield colorless crystals (223 mg, 51% yield).

^{1}H NMR (500 MHZ, DMSO) δ 8.29 (br s, 1H), 4.40-4.34 (m, 1H), 4.25-4.22 (d, 1H), 3.47-3.44 (dd, 1H), 1.23-1.22 (d, 3H)
^{13}C NMR (126 MHZ, DMSO) δ 202.84, 168.80, 57.93, 32.23, 14.37.
HRMS Calculated [M+H] 146.0276; Found [M+H] 146.0272

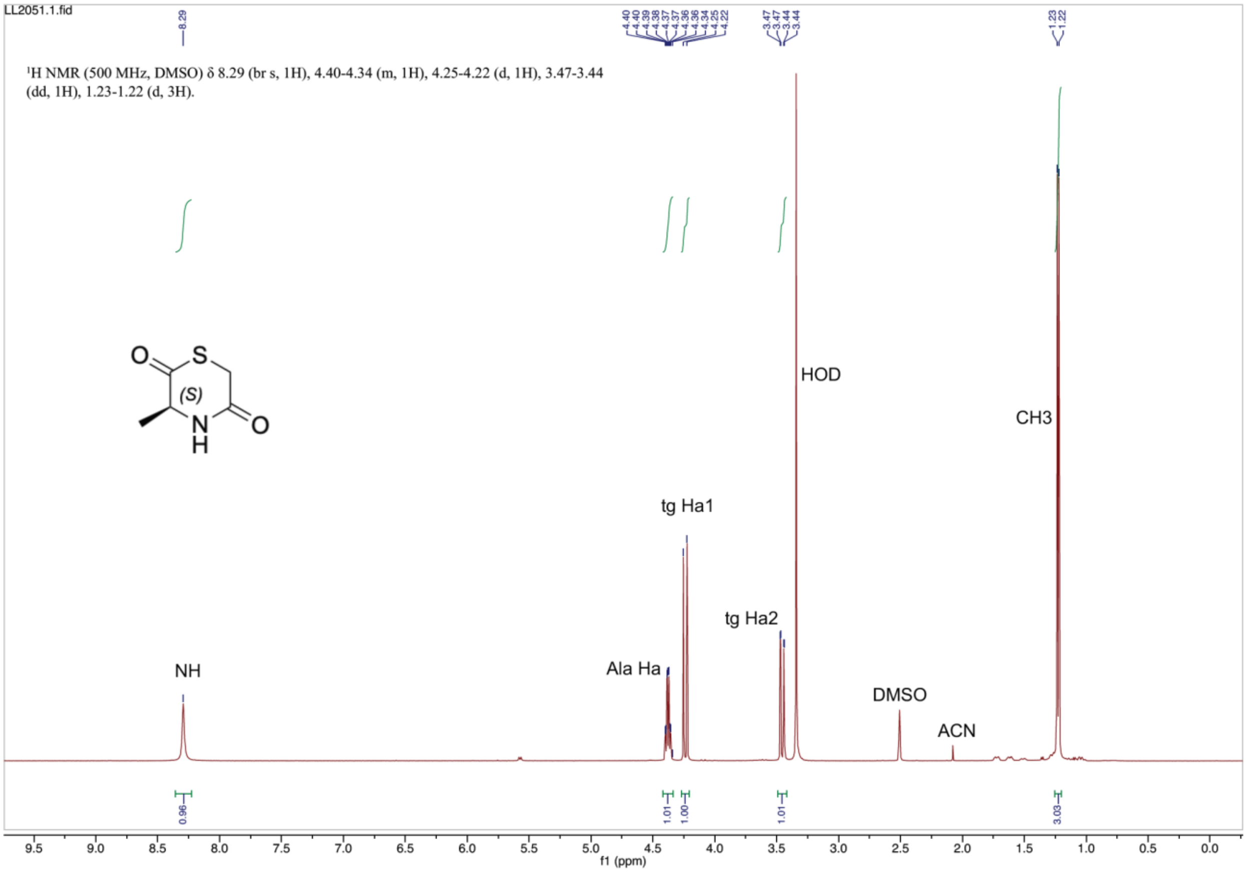
LL2051.1.fid
¹H NMR (500 MHz, DMSO) δ 8.29 (br s, 1H), 4.40-4.34 (m, 1H), 4.25-4.22 (d, 1H), 3.47-3.44 (dd, 1H), 1.23-1.22 (d, 3H).
(S)
HOD
CH3
tg Ha1
tg Ha2
NH
Ala Ha
DMSO
ACN
f1 (ppm)

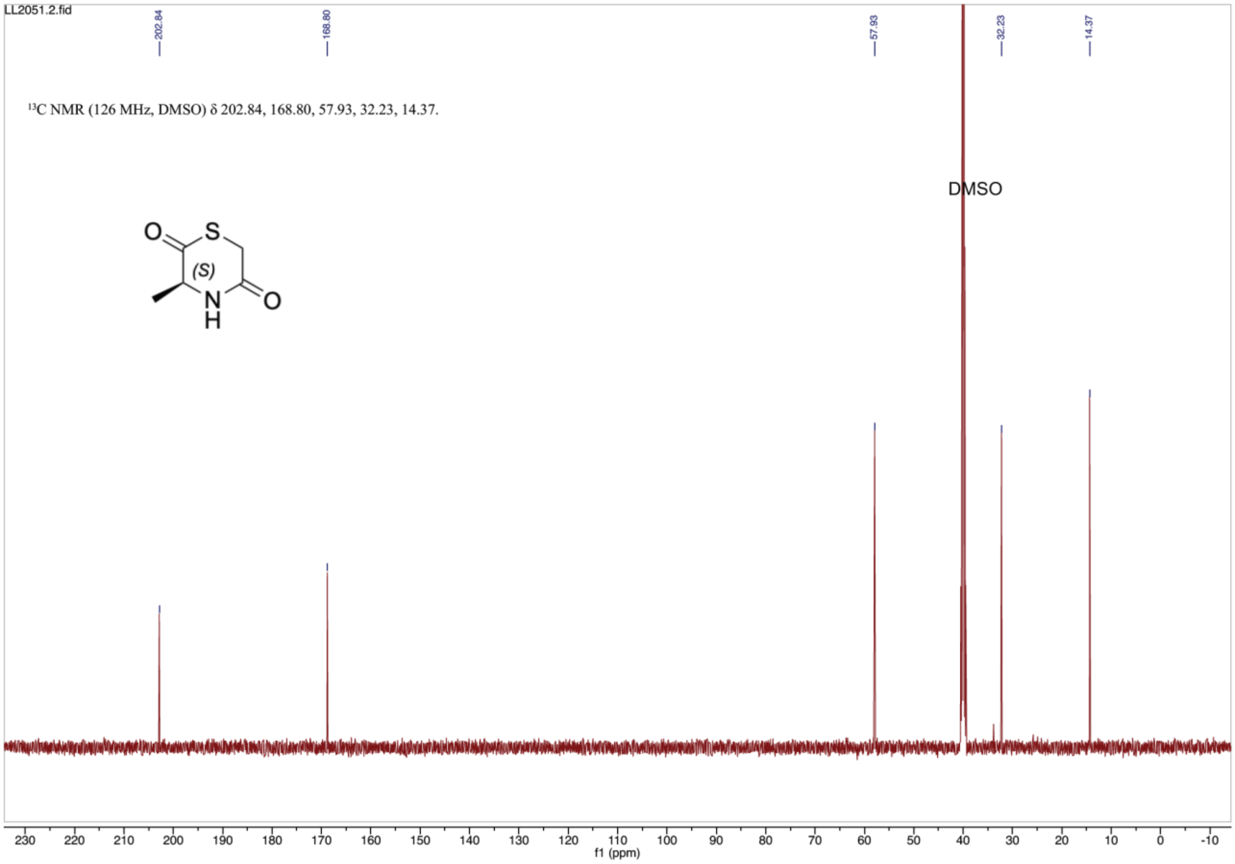

Crystal structure determination of (S)-3-methylthiazine-2,5-dione

The single crystal X-ray diffraction studies were carried out on a Bruker D8 Venture Ultra diffractometer equipped with Mo K_α radiation (λ = 0.71073). Crystals of the subject compound were used as received (grown from Acetonitrile). A 0.250 x 0.200 x 0.175 mm colorless block was mounted on a Cryoloop with Paratone oil.

Data were collected in a nitrogen gas stream at 100(2)K using ϕ and ϖ scans. Crystal-to-detector distance was 50 mm using exposure time 2s (depending on the detector 2θ position) with a scan width of 0.70°. Data collection was 99.8% complete to 25.242° in θ. A total of 14669 reflections were collected covering the indices, -9<=h<=9, -10<=k<=10, -13<=l<=13. 2831 reflections were found to be symmetry independent, with a R_{int} of 0.0395. Indexing and unit cell refinement indicated a Primitive, Monoclinic lattice. The space group was found to be $P2_1$. The data were integrated using the Bruker SAINT Software program and scaled using the SADABS software program. Solution by direct methods (SHELXT) produced a complete phasing model consistent with the proposed structure.

All nonhydrogen atoms were refined anisotropically by full-matrix least-squares (SHELXL-2014). All carbon bonded hydrogen atoms were placed using a riding model. Their positions were constrained relative to their parent atom using the appropriate HFIX command in SHELXL-2014. The data have been deposited to CCDC as a pre-publication file (Deposition Number #2078153).

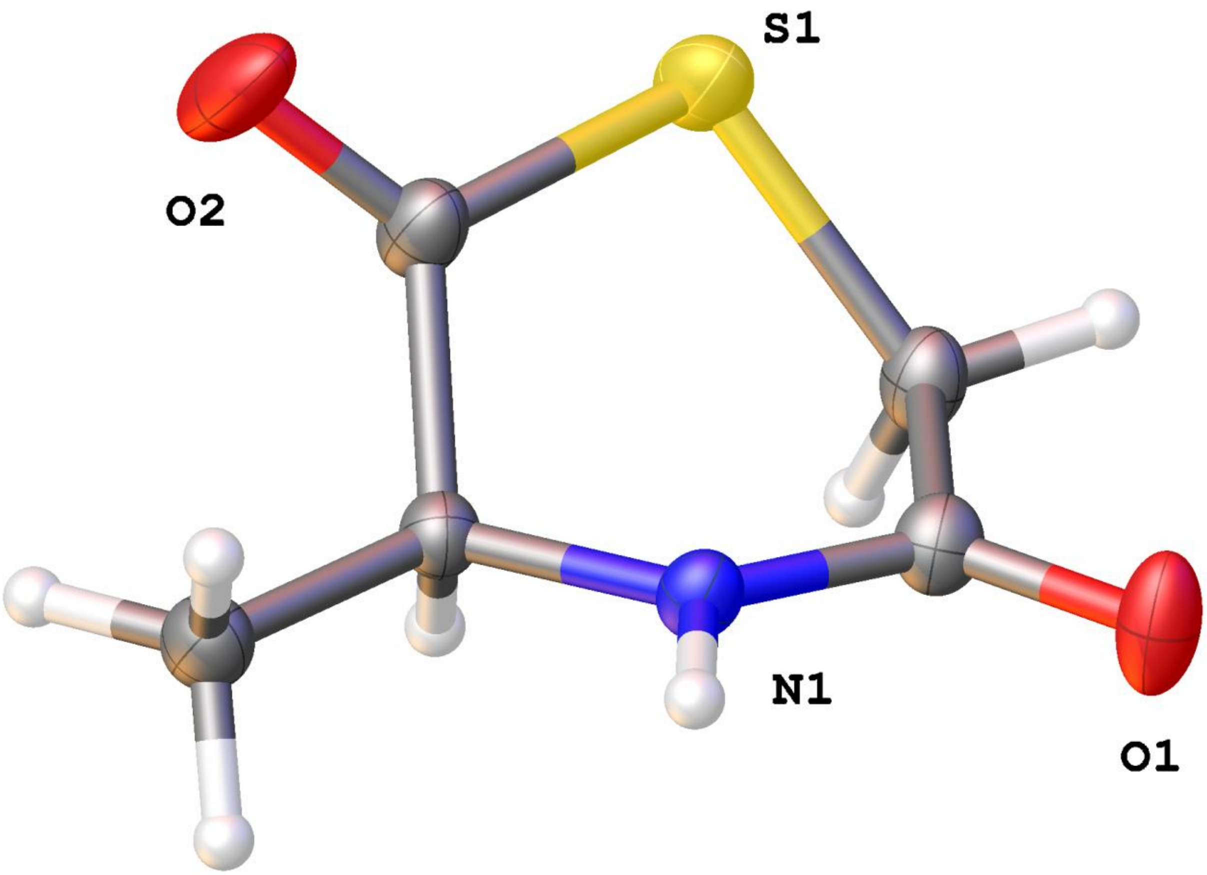

Supplementary Figures

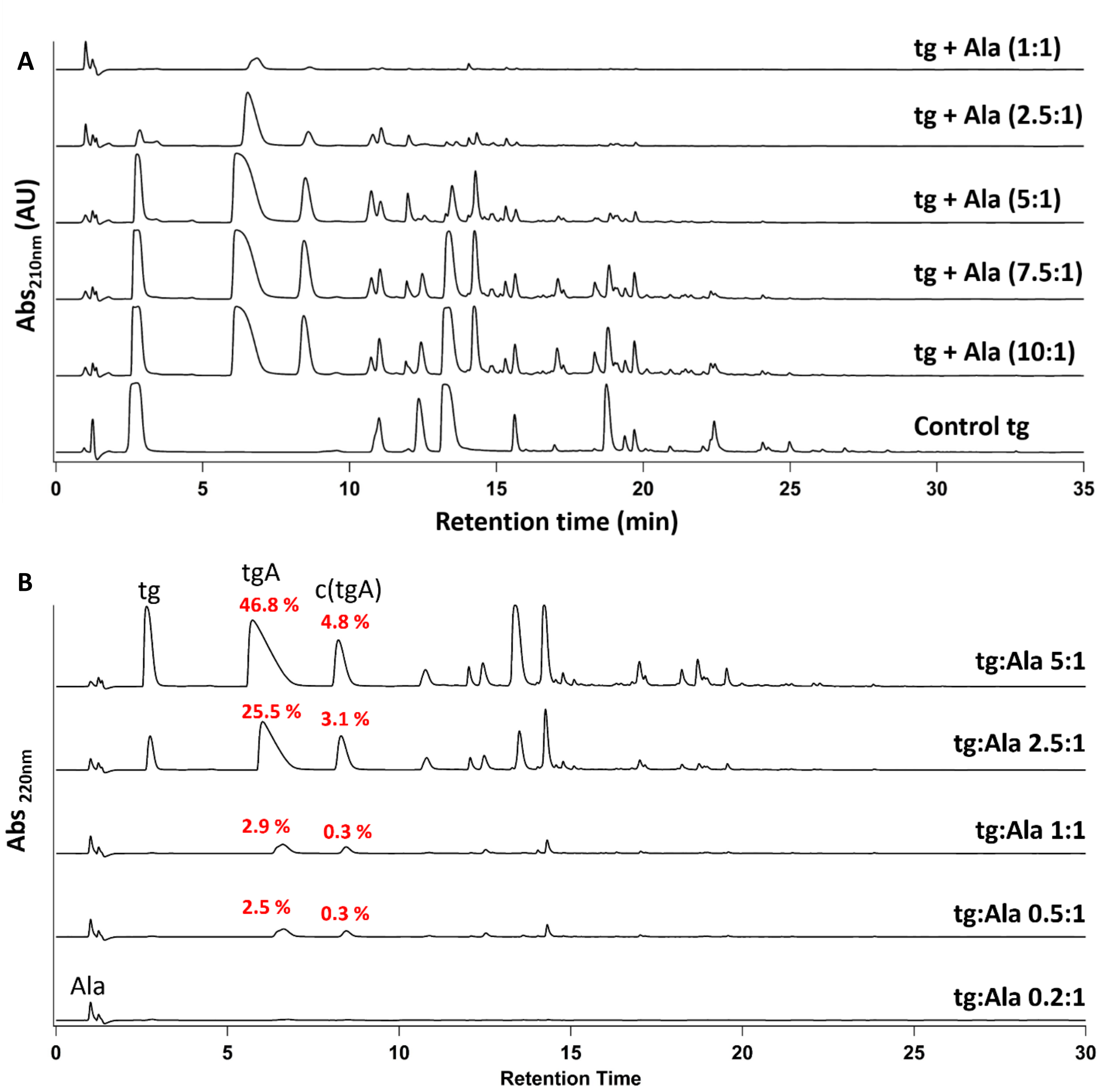

Supplementary Figure 1. C18-HPLC analysis shows greater yield of thiodepsipeptides following dry-down reactions of tg and Ala when tg is in excess. tg and L-Ala were dried at varying molar ratios, from 0.2:1 up to 10:1 (tg:Ala), at 65 ˚C for seven days and the resulting products were analyzed by hydrophobicity-based separation using C18-HPLC. Control tg reaction contained 10 eq of tg alone, in the absence of Ala. In panel B, percent conversion of Ala into tgA or c(tgA) is shown in red.

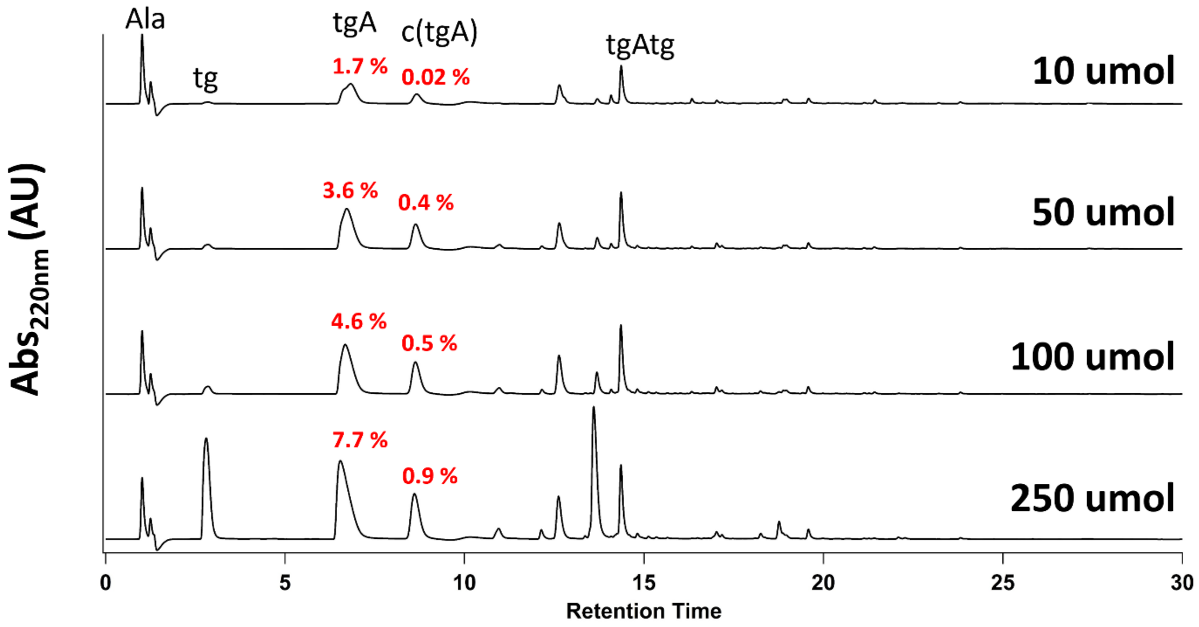

Supplementary Figure 2. C18-HPLC analysis of thiodepsipeptide formation following dry-down reactions of tg and Ala at 1:1 molar ratio at varying amounts. tg and L-Ala were dried at a 1:1 molar ratio at varying amounts (from 10 umol to 250 umol) at 65 ˚C for seven days and the resulting products were analyzed by hydrophobicity-based separation using C18-HPLC. Upon resuspension, samples were rehydrated with appropriate volumes to give 100mM Ala concentration (referring to initial amino acid concentration). Percent conversion of Ala into tgA or c(tgA) is shown in red. The results indicate that there is a positive correlation between the total starting amount and product yield, as well as loss of tg monomer as the starting amount of tg gets lower, suggesting that tg evaporates throughout the dry-heating process.

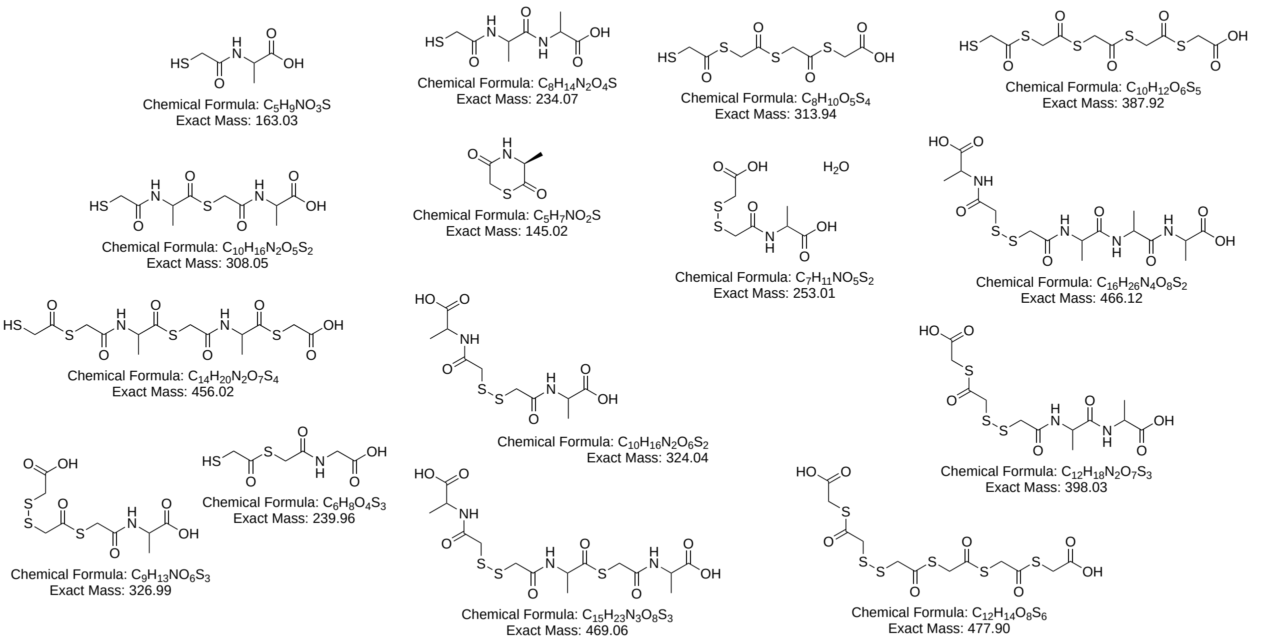

Supplementary Figure 3. Some of the possible products of a dry-down reaction of tg and Ala.

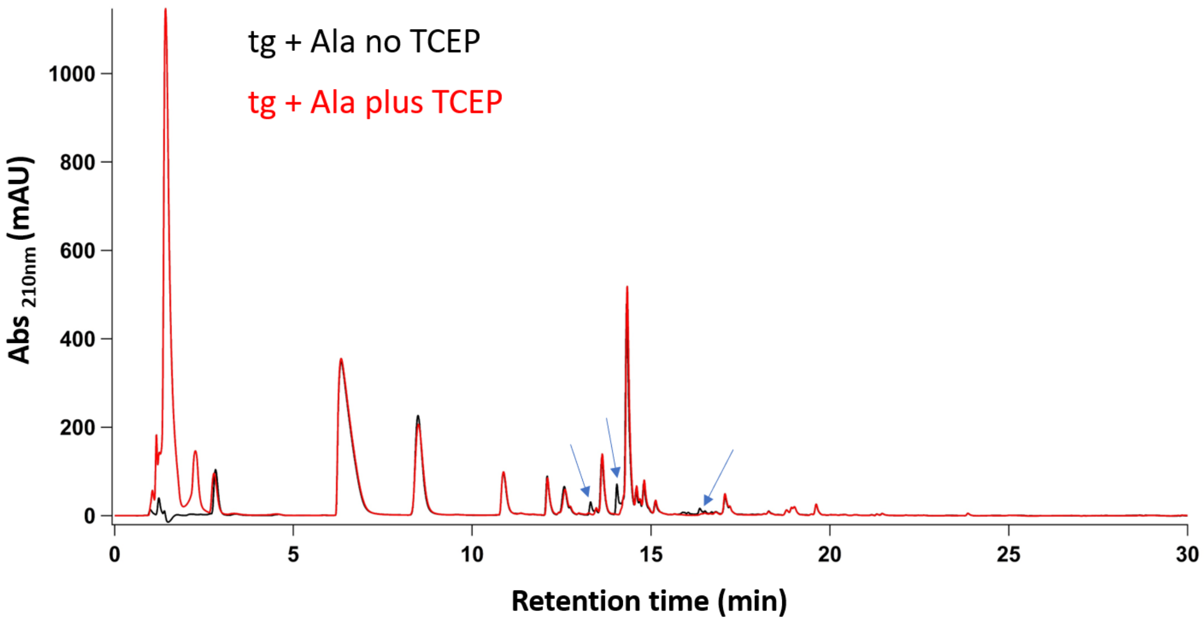

Supplementary Figure 4. Minimal oxidation occurs upon dry-down reactions under experimental anoxic conditions. tg and L-Ala were dried at a 5:1 molar ratio (in favor of tg) at 65 ˚C for seven days and the resulting products were analyzed by hydrophobicity-based separation using C18-HPLC before (black) and after (red) incubation with 500 mM TCEP for 1 hr at RT. Blue arrows indicate species that were reduced with TCEP.

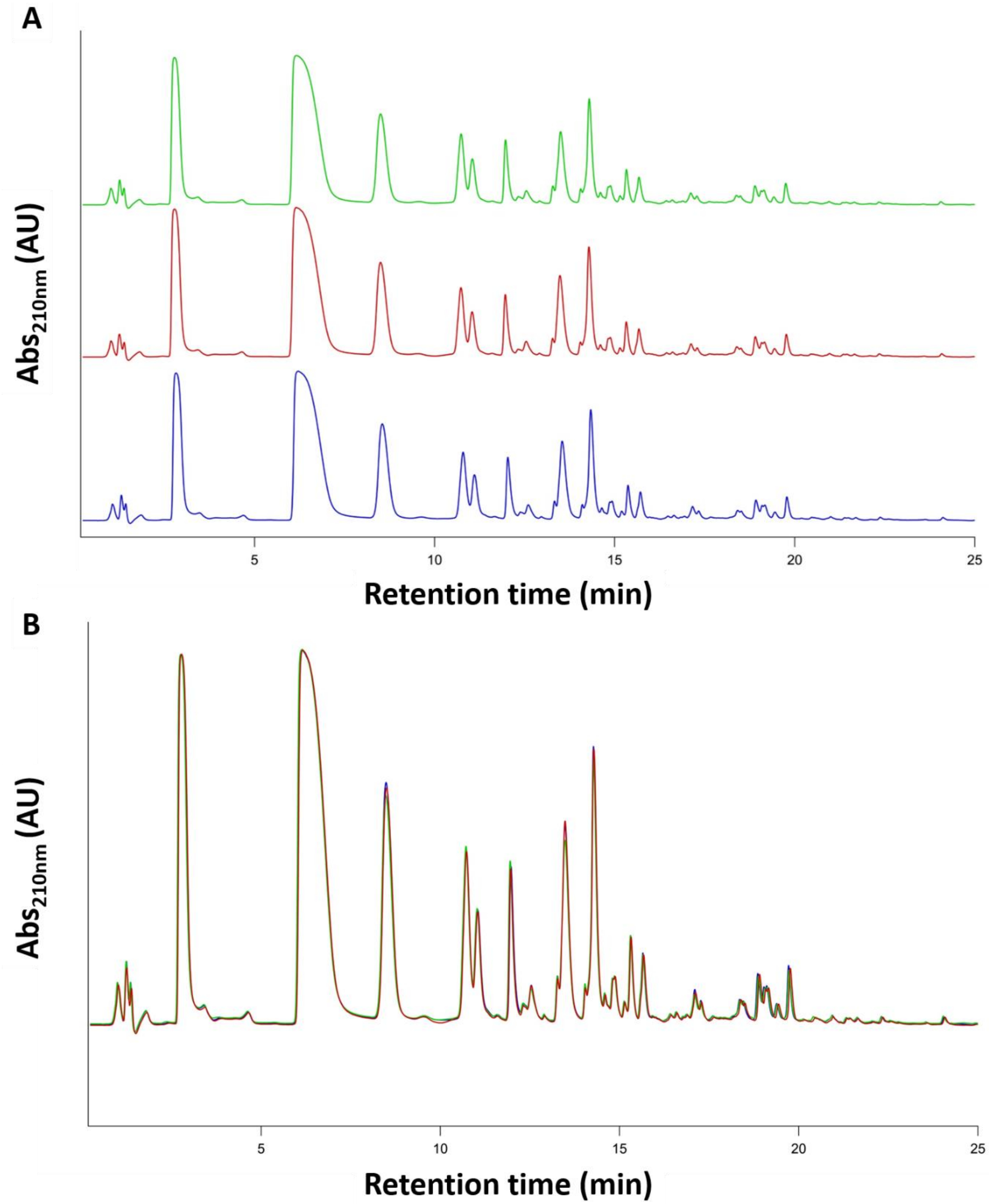

Supplementary Figure 5. High reproducibility of product mixture is observed upon dry-down of mercaptoacid and amino acids. Three independent replicates of dry-down reactions of tg and Ala at a 5:1 molar ratio (tg:Ala) at 65 ˚C for seven days shows very high reproducibility by hydrophobicity-based separation using C18-HPLC. The three replicate experiments are shown as a stack (A) and overlaid (B).

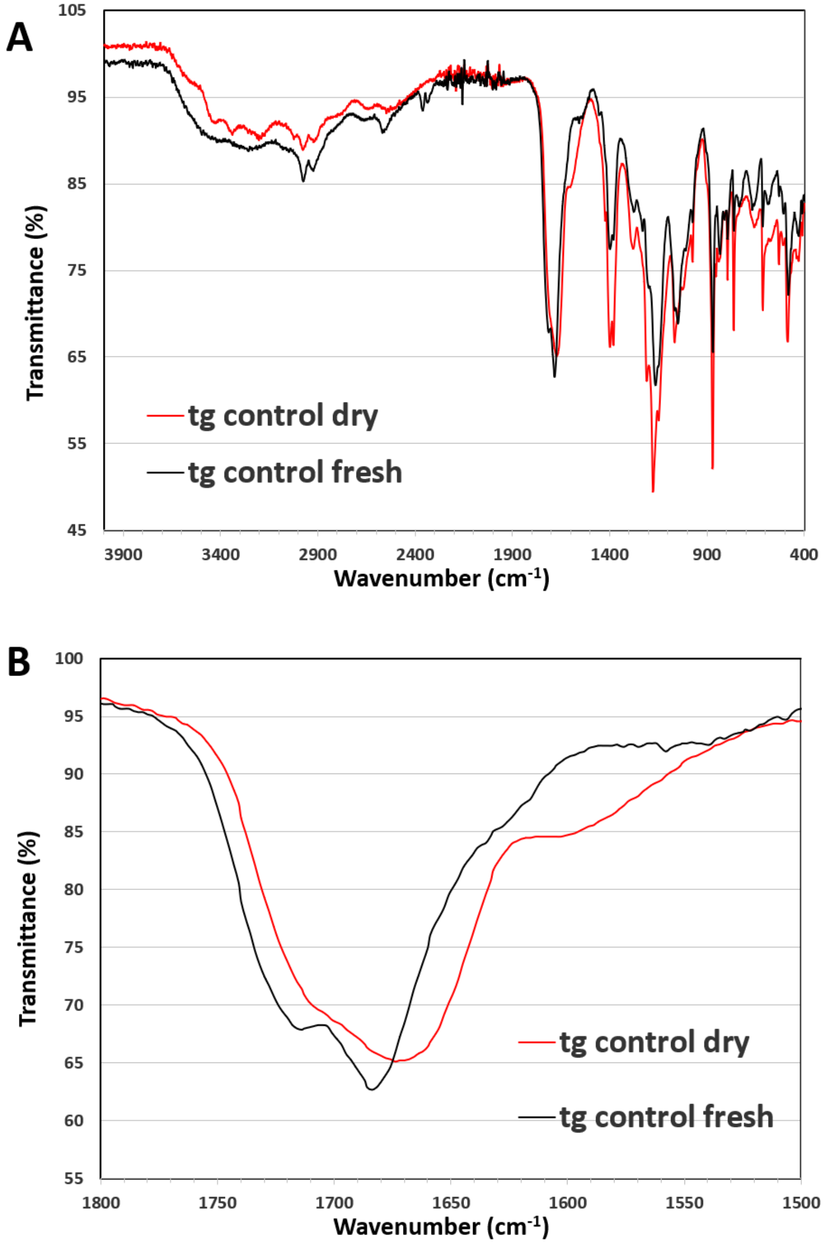

Supplementary Figure 6. Fourier Transform Infrared Spectroscopy (FTIR) shows shifts in the C=O stretch upon dry-down of tg, supportive of thioester formation. tg was dried at 65 ˚C for seven days and the resulting mixture was analyzed by FTIR (A-B). Panel B is an enlargement of the 1500-1800 cm^{-1} region. Thioesters have lower carbonyl stretching frequencies than esters (2). Upon dry-down of tg, the C=O stretch shifts from a carboxylic acid to lower frequencies, as expected following formation of thioesters (2, 3). Changes were also observed in the thiol S-H stretch (~2570 cm^{-1}) following the dry-down of tg (4, 5).

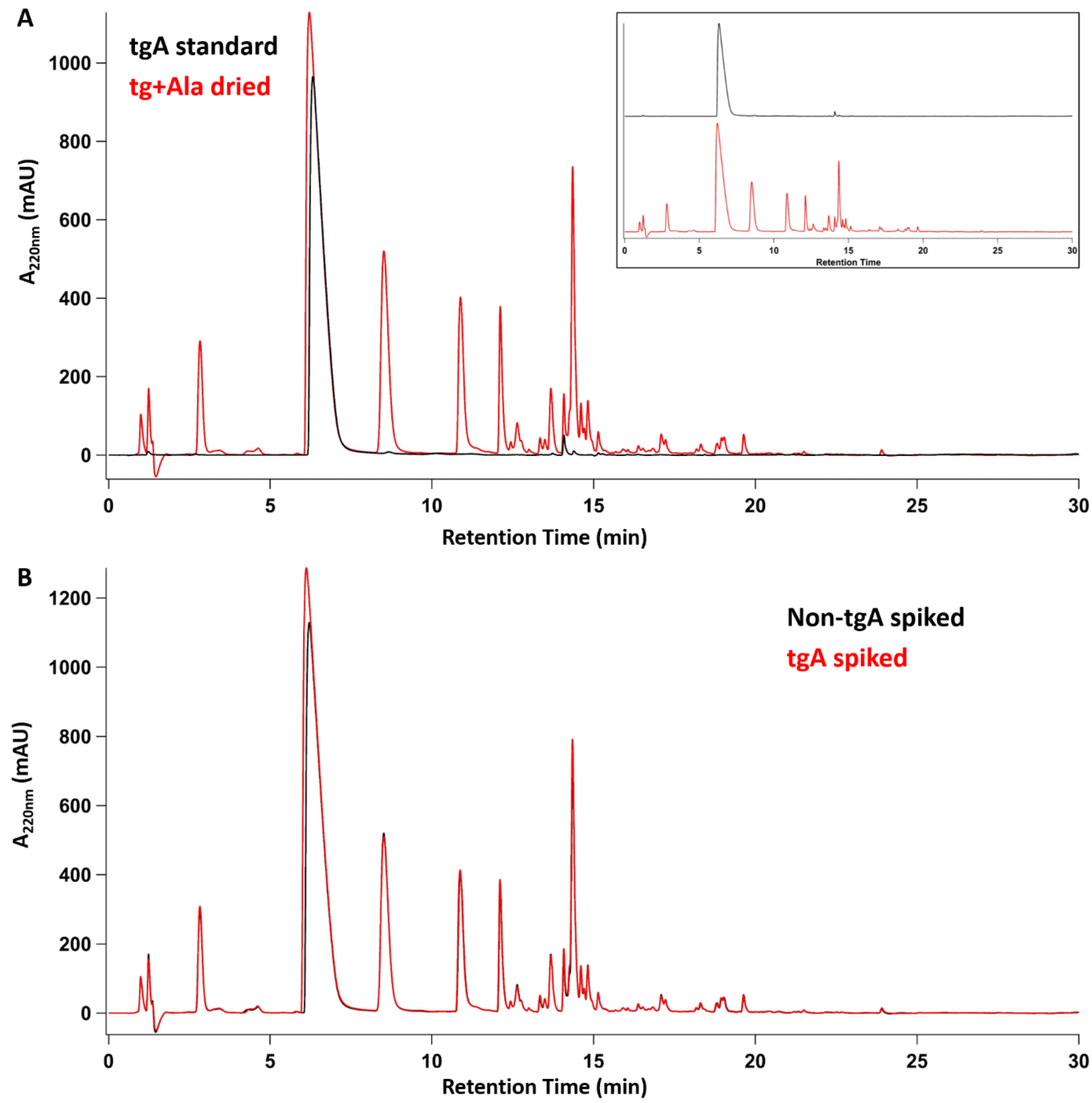

Supplementary Figure 7. Validation of the formation of tgA peptide upon dry-down of tg with Ala. (A) Comparison between a synthesized tgA peptide (10 mM), composed of a terminal tg linked with an amide to Ala (*black* trace), to product mixture formed by drying of tg with Ala at a 5:1 molar ratio (tg:Ala) at 65 ˚C for seven days (*red* trace). Separation via hydrophobicity-based C18-HPLC confirms the formation of the tgA peptide. (B) Overlay of a product mixture formed by drying of tg with Ala at a 5:1 molar ratio at 65 ˚C for seven days (*black* trace) compared to the same mixture that was spiked with 1mM tgA (*red* trace), also supports the formation of tgA upon drying of tg with Ala.

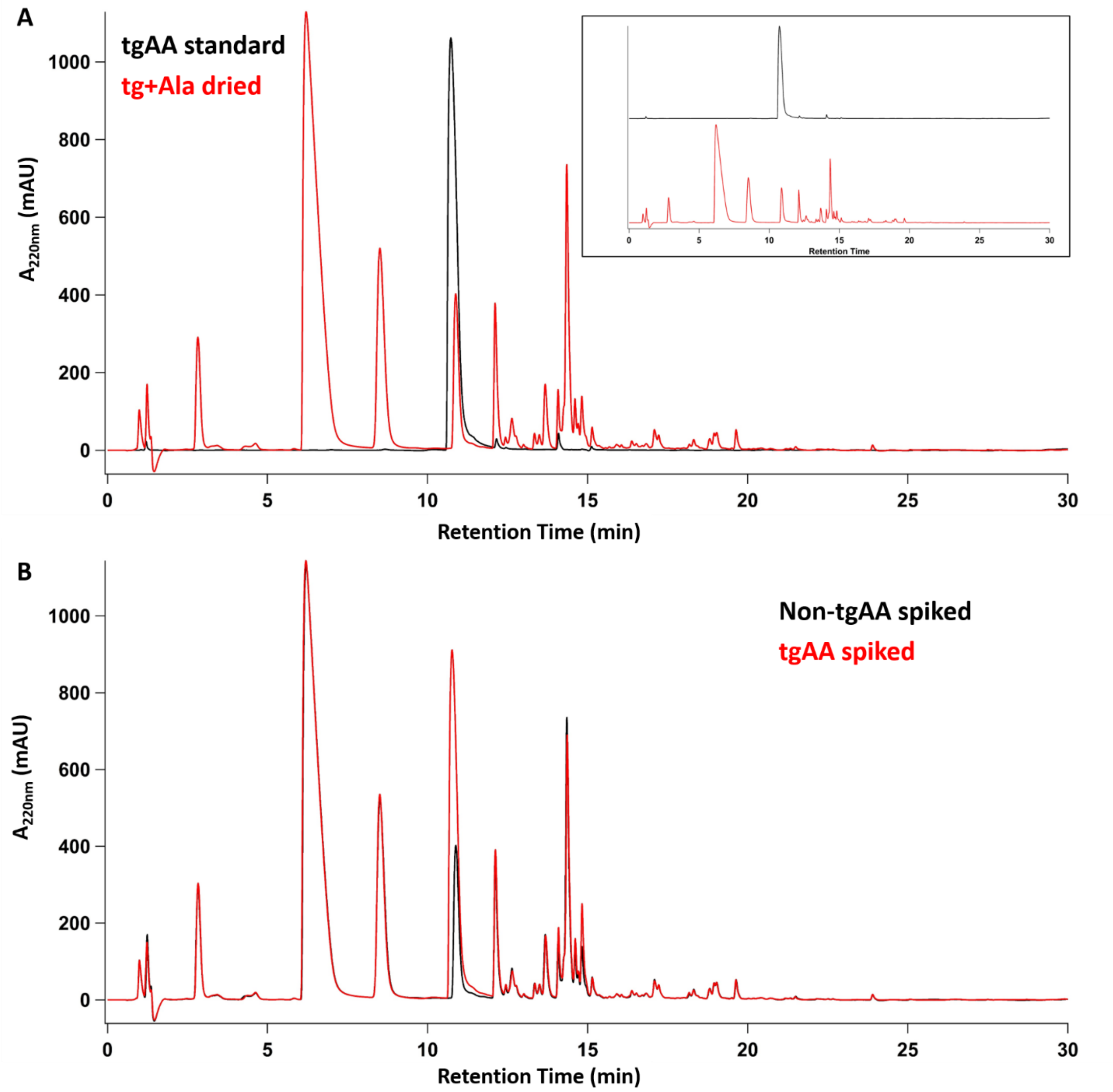

Supplementary Figure 8. Validation of the formation of tgAA peptide upon dry-down of tg with Ala. (A) Comparison between a synthesized tgAA peptide (10 mM), composed of a terminal tg linked with two consecutive amides to Ala (*black* trace), to product mixture formed by drying of tg with Ala at a 5:1 molar ratio (tg:Ala) at 65 ˚C for seven days (*red* trace). Separation via hydrophobicity-based C18-HPLC confirms the formation of the tgAA peptide. (B) Overlay of a product mixture formed by drying of tg with Ala at a 5:1 molar ratio at 65 ˚C for seven days (*black* trace) compared to the same mixture that was spiked with 1mM tgAA (*red* trace), also supports the formation of tgAA upon drying of tg with Ala.

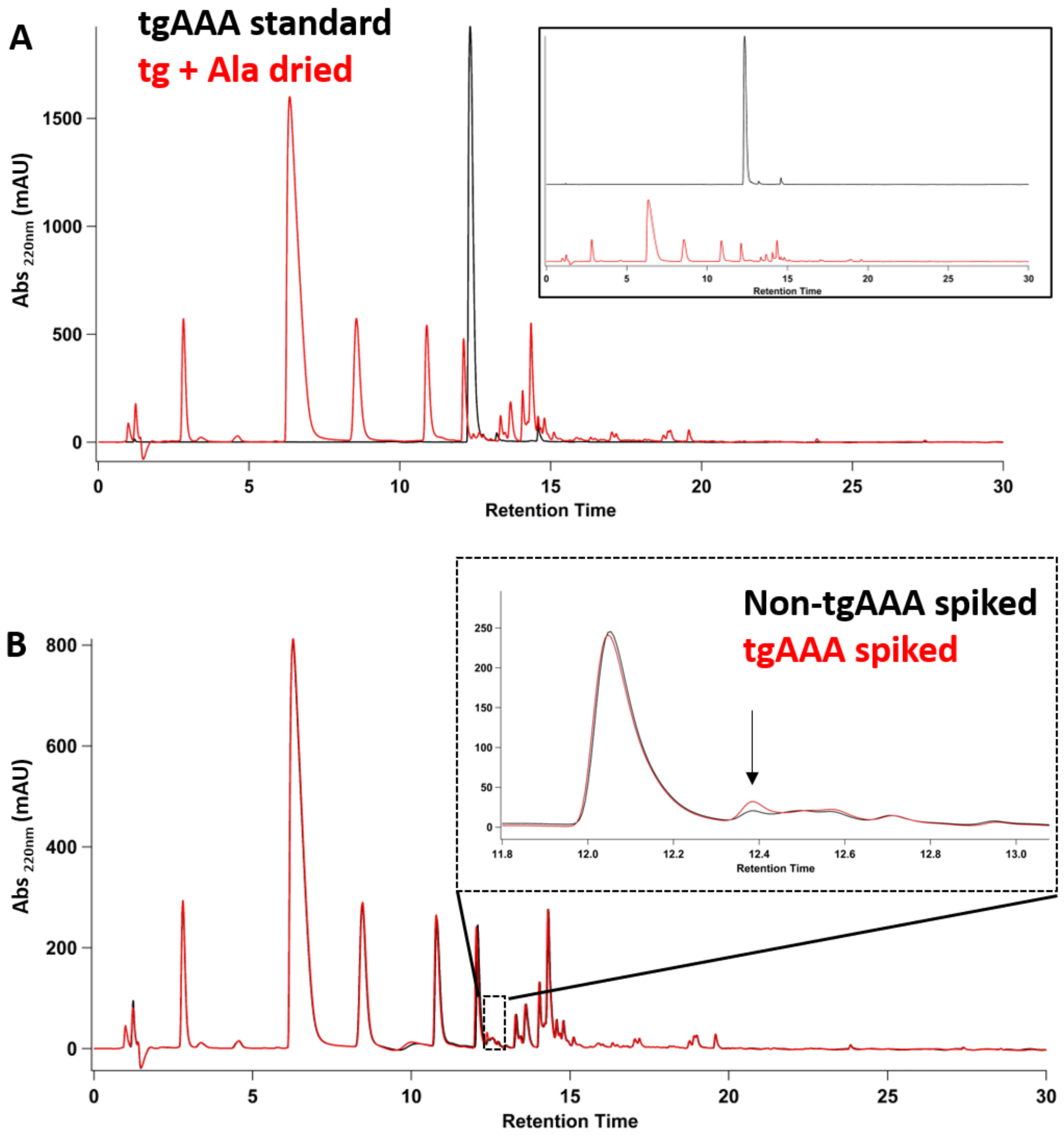

Supplementary Figure 9. Validation of the formation of tgAAA peptide upon dry-down of tg with Ala. (A) Comparison between a synthesized tgAAA peptide (10 mM), composed of a terminal tg linked with three consecutive amides to Ala (*black* trace), to product mixture formed by drying of tg with Ala at a 5:1 molar ratio (tg:Ala) at 65 ˚C for seven days (*red* trace). Separation via hydrophobicity-based C18-HPLC confirms the formation of the tgAAA peptide. (B) Overlay of a product mixture formed by drying of tg with Ala at a 5:1 molar ratio at 65 ˚C for seven days (*black* trace) compared to the same mixture that was spiked with 0.05 mM tgAAA (*red* trace), also supports the formation of tgAAA upon drying of tg with Ala.

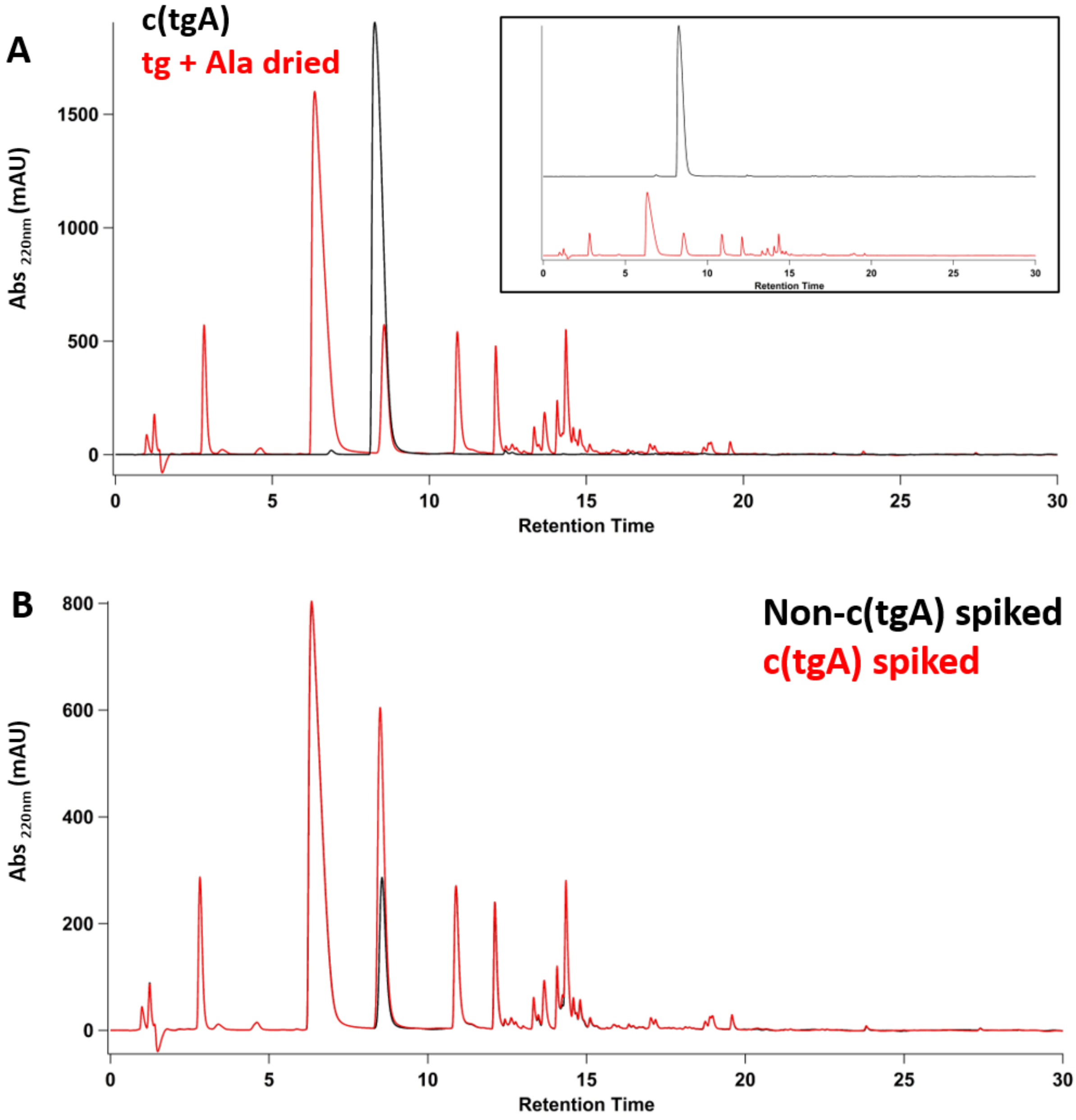

Supplementary Figure 10. Validation of the formation of c(tgA) upon dry-down of tg with Ala. (A) Comparison between a synthesized (S)-3-methylthiazine-2,5-dione (c(tgA), 10 mM, *black* trace), to product mixture formed by drying of tg with Ala at a 5:1 molar ratio (tg:Ala) at 65 ˚C for seven days (*red* trace). Separation via hydrophobicity-based C18-HPLC confirms the formation of c(tgA). (B) Overlay of a product mixture formed by drying of tg with Ala at a 5:1 molar ratio at 65 ˚C for seven days (*black* trace) compared to the same mixture that was spiked with 1mM c(tgA) (*red* trace), also supports the formation of c(tgA) upon drying of tg with Ala.

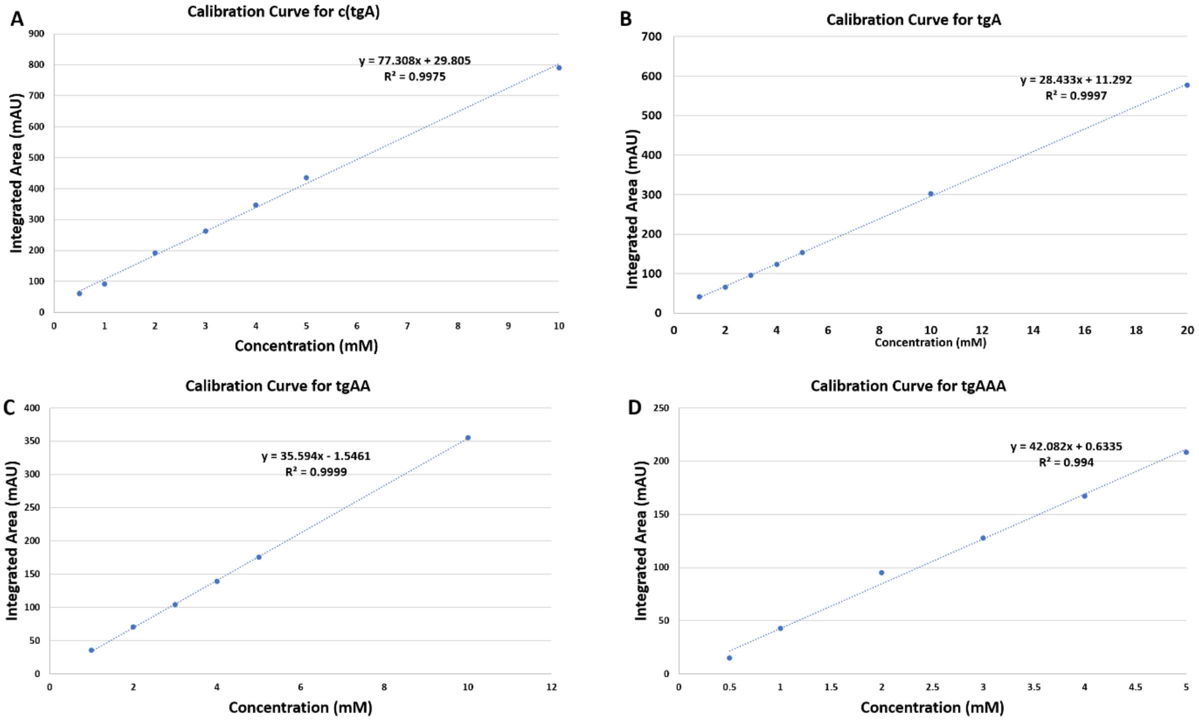

E

compound	area	equation	concentration	yield (%)
tgA	653.76	y = 28.433x + 11.292	22.59585693	45.19171
c(tgA)	142.57	y = 77.308x + 29.805	1.458645936	2.917292
tgAA	109.53	y = 35.594x - 1.5461	3.033766927	6.067534
tgAAA	4.2475	y = 42.082x + 0.6335	0.085879949	0.17176

Supplementary Figure 11. Quantification of products from dry-down reactions using calibration curves of synthesized standards. Calibration curves were performed for four synthesized standards: A) c(tgA), B) tgA, C) tgAA, and D) tgAAA and were used to calculate product yield (E) following dry-down reactions of Ala and tg at 65 °C for seven days (Figure 2c in main text).

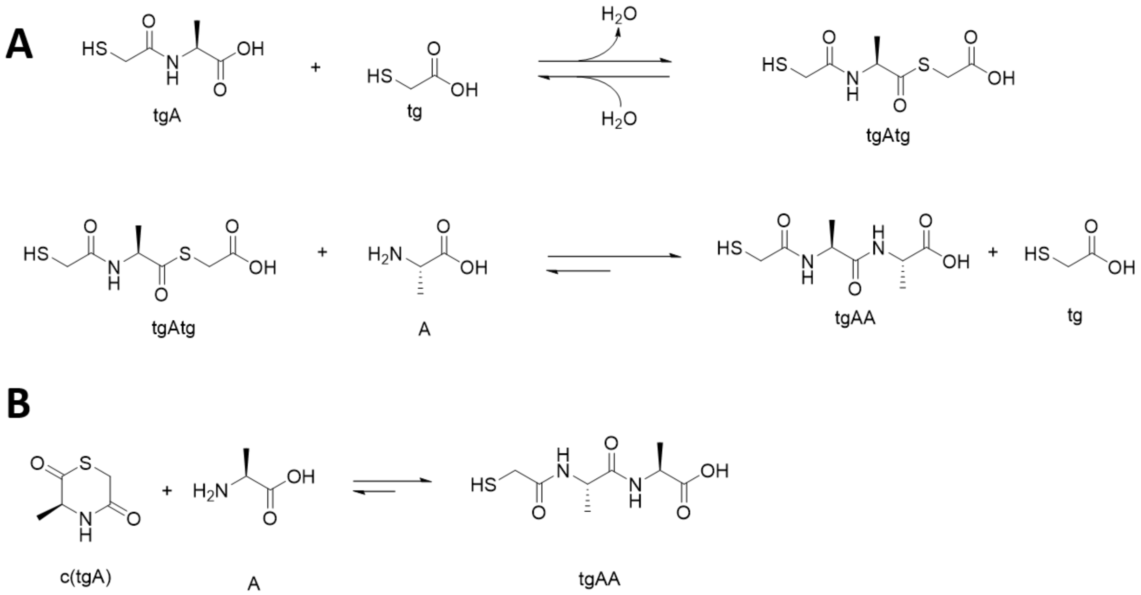

Supplementary Figure 12. Two plausible pathways for acyl substitutions for tgAA formation through thioester-amide exchange. A) tgA condenses to form tgAtg in dry conditions, and the thioester can be exchanged for amide bonds in the presence of alanine (A) to form tgAA. B) The thioester c(tgA) can be ring-opened to form tgAA in the presence of alanine (A).

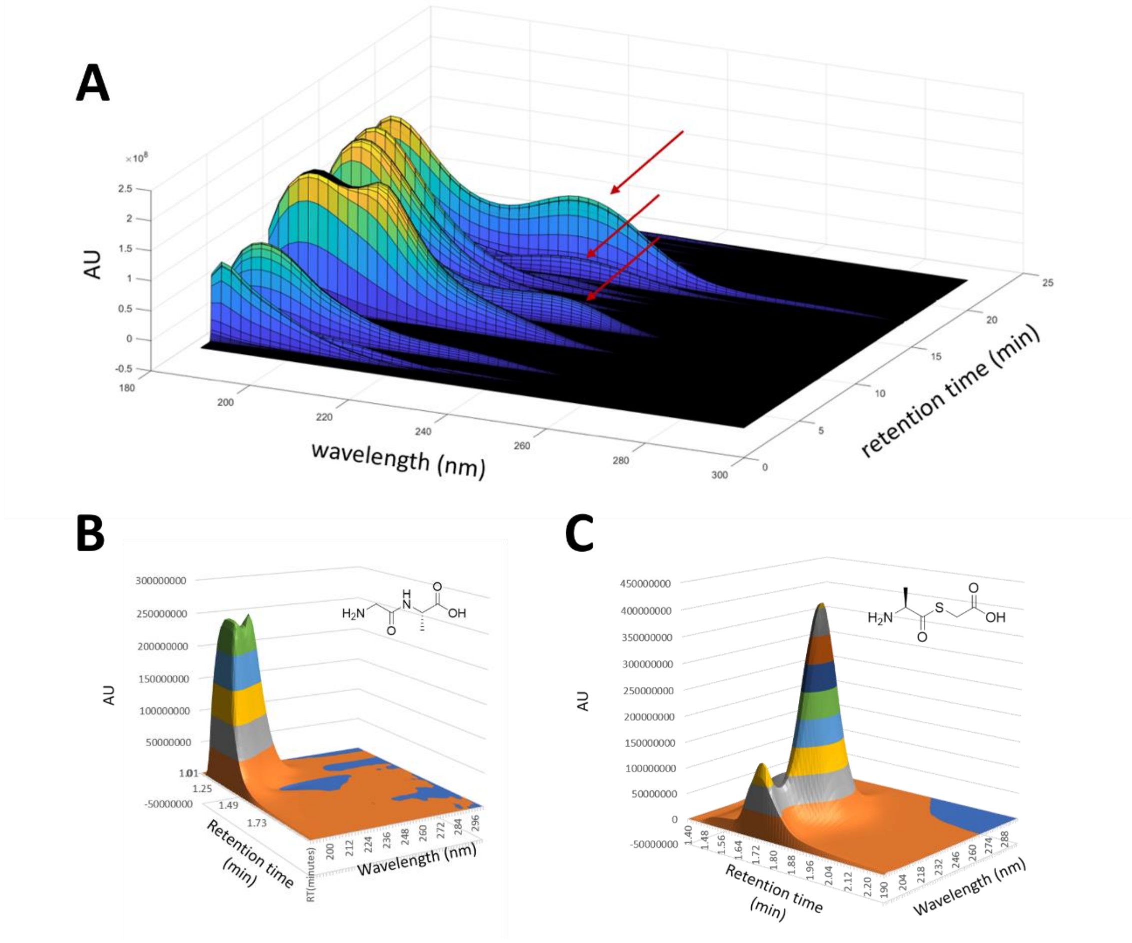

Supplementary Figure 13. Confirmation of thioester-containing compounds upon dry-down reactions of tg and Ala. A) tg and Ala were dried at 65 °C for seven days and the resulting products were separated via C18-HPLC. The existence of thioester-containing compounds was verified via collection of full UV-VIS spectrum during the HPLC analysis, which demonstrated the existence of various peaks with the typical thioester maximal absorbance at ~235nm (examples are indicated with arrows). B) Full UV-VIS spectrum of an amide-containing standard (Gly-Ala dipeptide). C) Full UV-VIS spectrum of a thioester-containing standard (Atg).

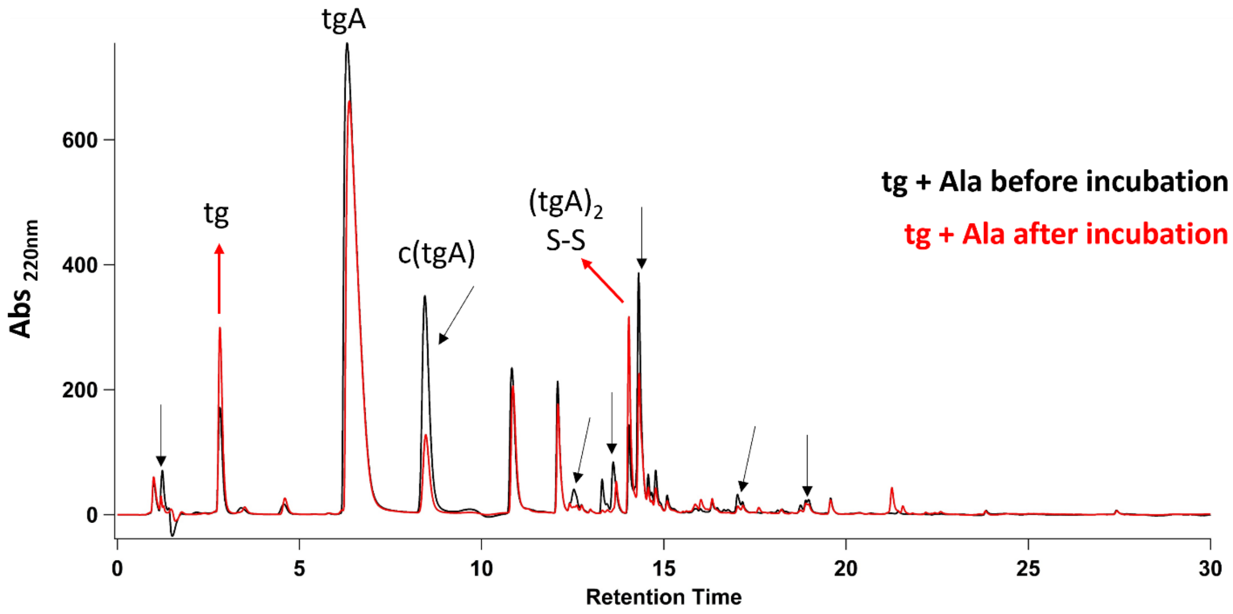

Supplementary Figure 14. Hydrolysis occurs upon incubation of dry-heated reactions in water. tg and L-Ala were dried at a 5:1 molar ratio (in favor of tg) at 65 ˚C for seven days and the resulting products were rehydrated and incubated in water for 3 days at 65°C (unbuffered, pH~3.5) under anoxic conditions. Samples were analyzed by hydrophobicity-based separation using C18-HPLC before (*black*) and after (*red*) incubation in water. Black arrows indicate species that hydrolyzed. Red arrow indicates tg, which increased in abundance following hydrolysis.

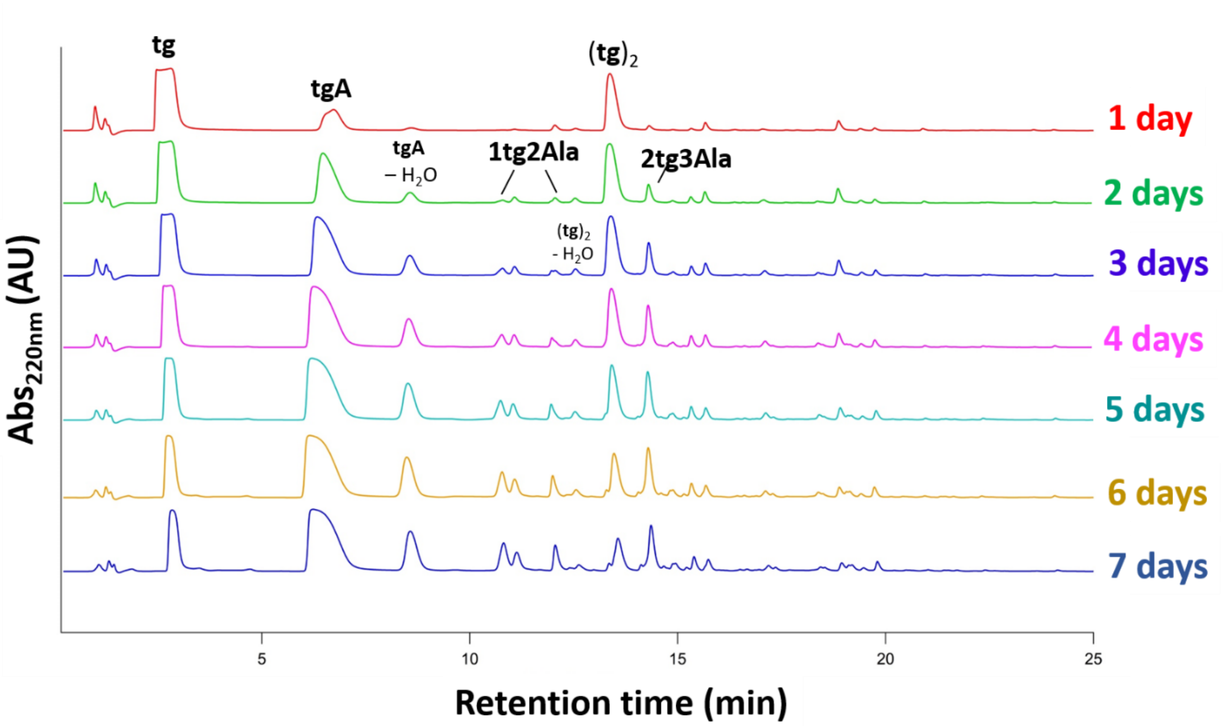

Supplementary Figure 15. Kinetics of thiodepsipeptide formation over time. tg and Ala were dried at a 5:1 molar ratio (tg:Ala) at 65 ˚C for up to seven days. Gradual polymer formation is shown by hydrophobicity-based separation using C18-HPLC.

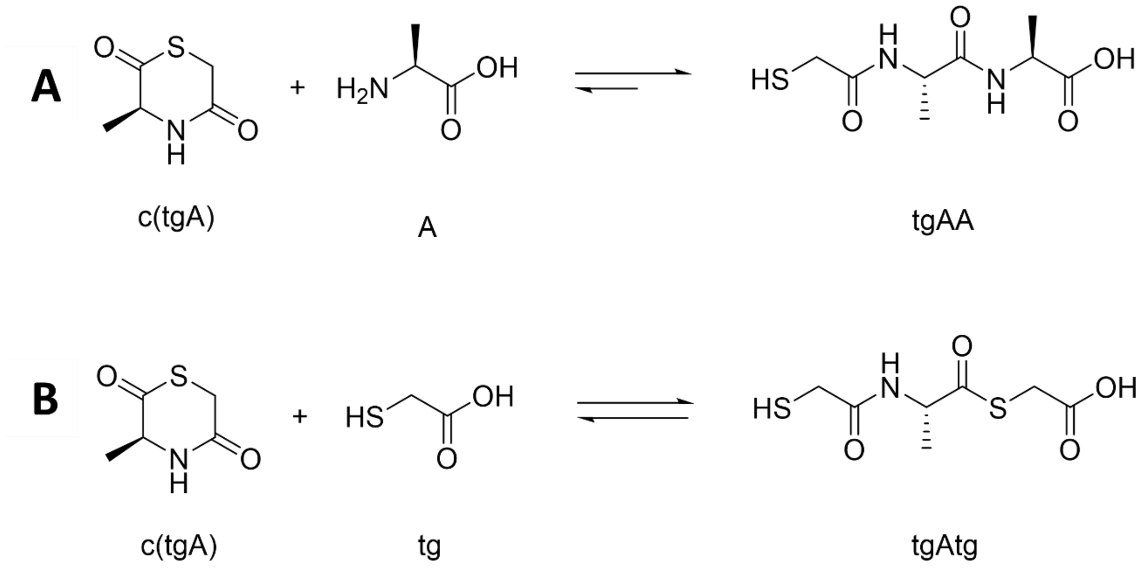

Supplementary Figure 16. Proposed ring-opening polymerization pathways using c(tgA). The thioester c(tgA) can be ring-opened to form (A) tgAA in the presence of alanine or tgAtg (B) in the presence of tg.

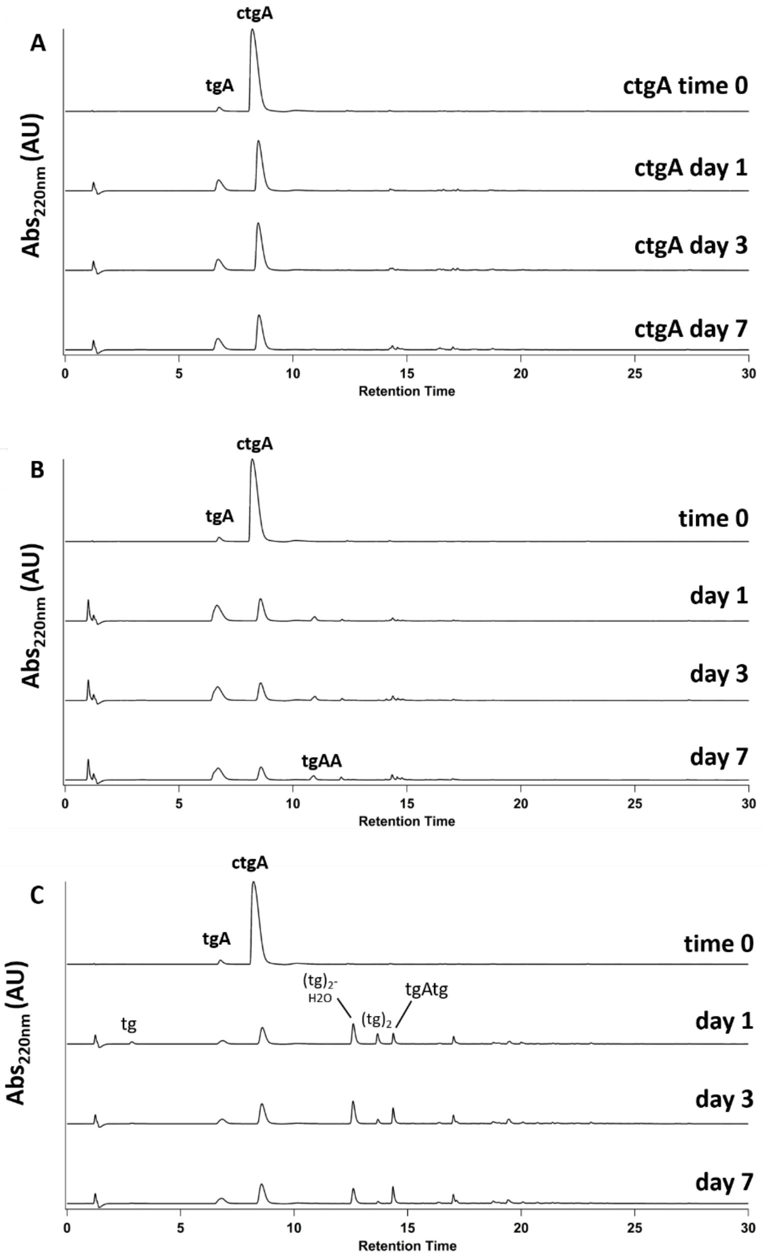

Supplementary Figure 17. Dry-down reactions of c(tgA) in the presence or absence of tg or alanine. c(tgA) was dry-heated at 65°C for up to seven days in the absence (A) or presence of either alanine (B) or tg (C) at a 1:10 molar ratio (in favor of either tg or alanine). Gradual polymer formation is shown by hydrophobicity-based separation using C18-HPLC. Indeed, fast formation of products that are expected to result from ring-opening polymerization appeared following dry-down. Specifically, tgAA is formed upon reaction of c(tgA) with A and tgAtg is formed upon reaction of c(tgA) with tg. Some hydrolysis of the c(tgA) thioester is observed which leads to formation of the linear tgA form.

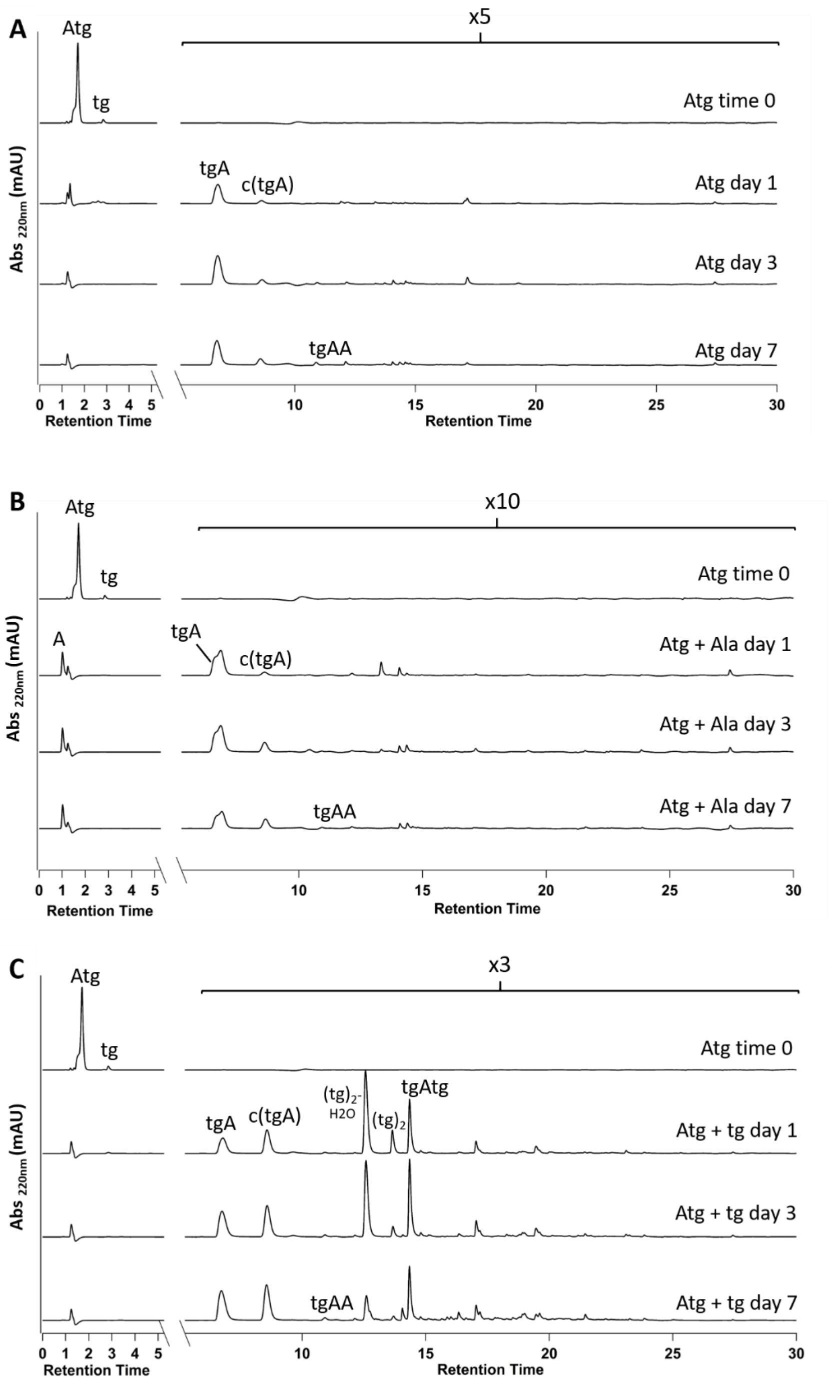

Supplementary Figure 18. Dry-down reactions of Atg in the presence or absence of tg or alanine. Atg was dry-heated at 65°C for up to seven days in the absence (A) or presence of either alanine (B) or tg (C) at a 1:10 molar ratio (in favor of either tg or alanine). Gradual polymer formation is shown by hydrophobicity-based separation using C18-HPLC. Notably, Atg hydrolyzes very quickly followed by rearrangement into tgA from free tg and Ala.

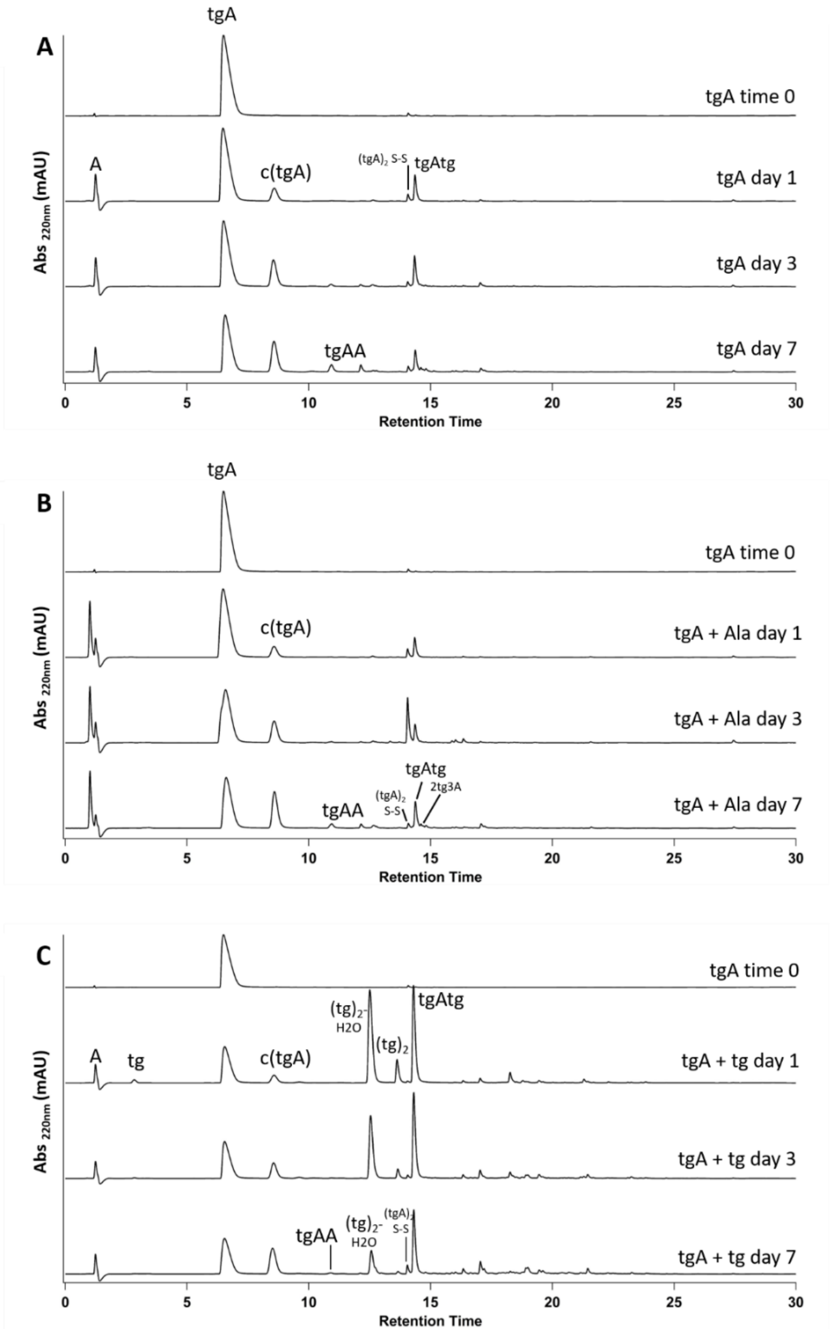

Supplementary Figure 19. Dry-down reactions of tgA in the presence or absence of tg or alanine. tgA was dry-heated at 65°C for up to seven days in the absence (A) or presence of either alanine (B) or tg (C) at a 1:10 molar ratio (in favor of either tg or alanine). Gradual polymer formation is shown by hy hydrophobicity-based separation using C18-HPLC. Notably, some amide hydrolysis of the tgA standard occurred during the dry-heating process (e.g. the tgAA product is observed).

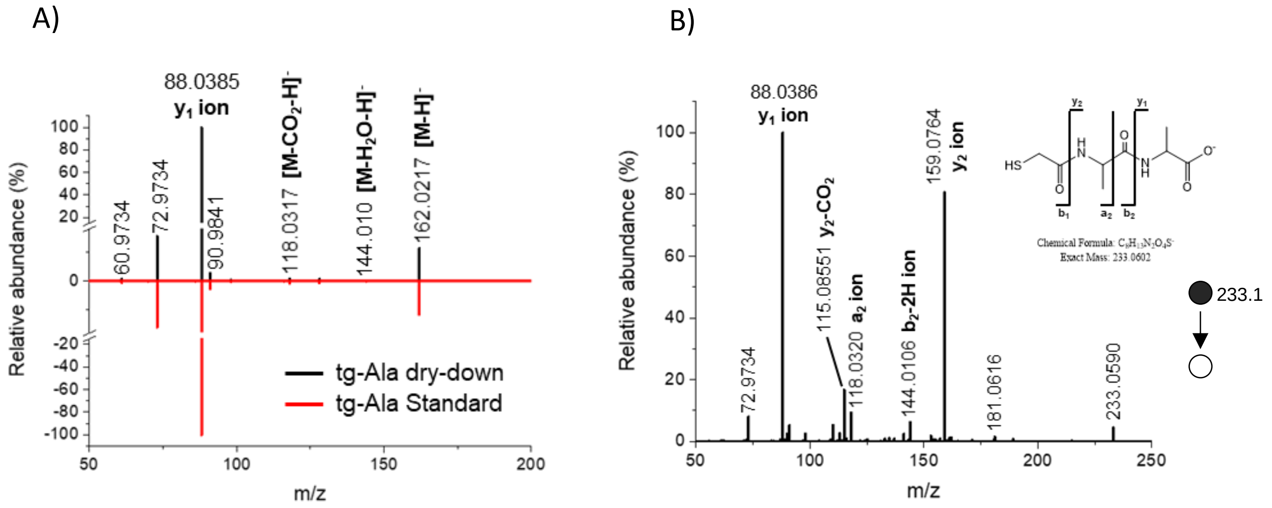

Supplementary Figure 20. Verification of formation of amide bonds in dry-down reactions of mercaptoacids and amino acids using MS/MS analysis. tg and Ala were dried at a 5:1 molar ratio (tg:Ala) at 65 ˚C for seven days and the resulting products were subjected to MS/MS analysis. (A) MS/MS spectra is shown for tgA formed during the dry-down reaction (*black* trace) compared to that of a synthetized tgA peptide standard (*red* trace). (B) Tandem MS of *m/z* 233.1 corresponding to [M-H]$^-$ of 1tg2Ala exhibited major fragment ions for the sequence tgAA, further supporting the formation of amide bonds in dry-down reactions; *y*, *b* and *a* ions confirmed a terminal tg followed by two Ala residues.

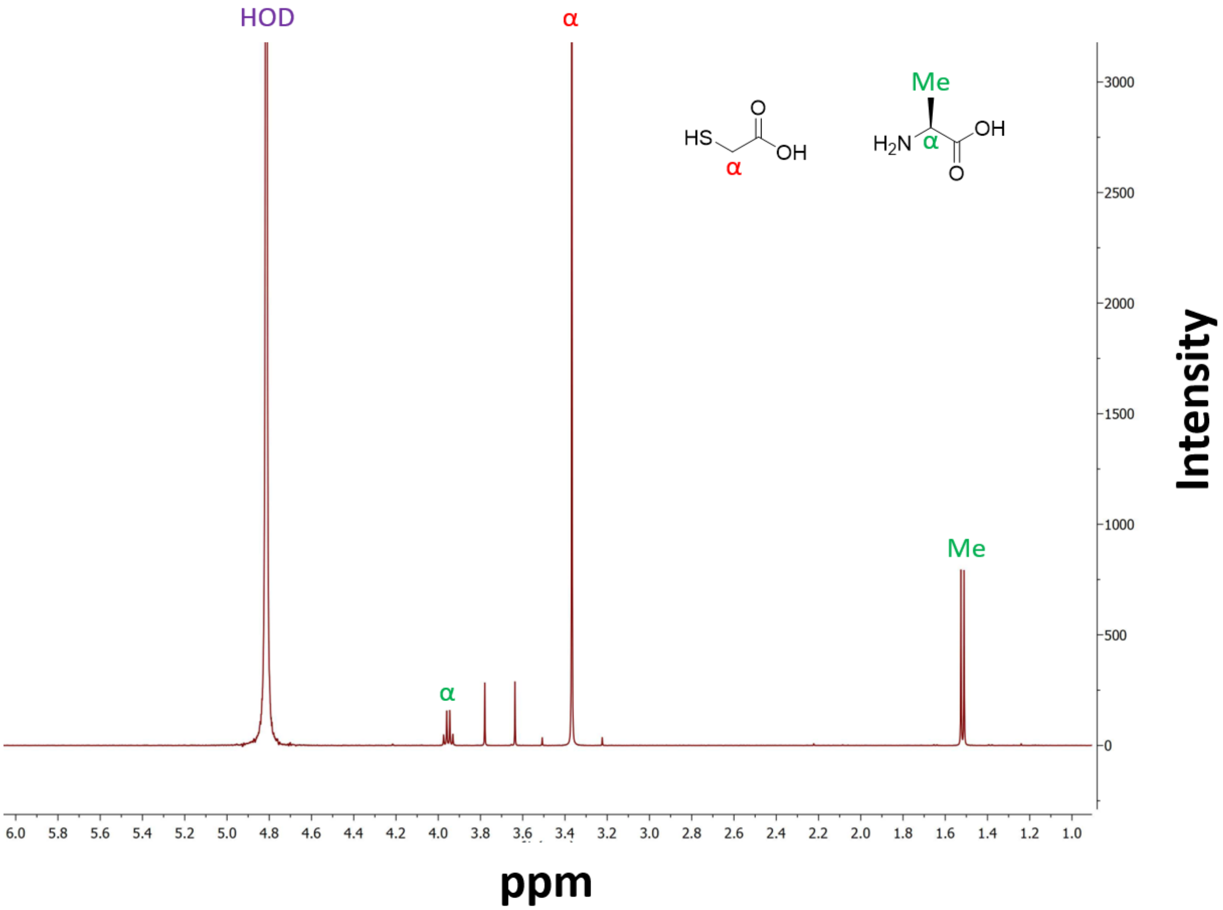

Supplementary Figure 21. Full spectrum of a fresh, non-dried tg plus Ala mixture. A fresh mixture of unreacted tg and Ala at a 5:1 molar ratio (tg:Ala) was prepared in a phosphate buffer in D_2O (pH 2.5). The free α-proton chemical shift of Ala is centered at 3.95 parts per million (ppm).

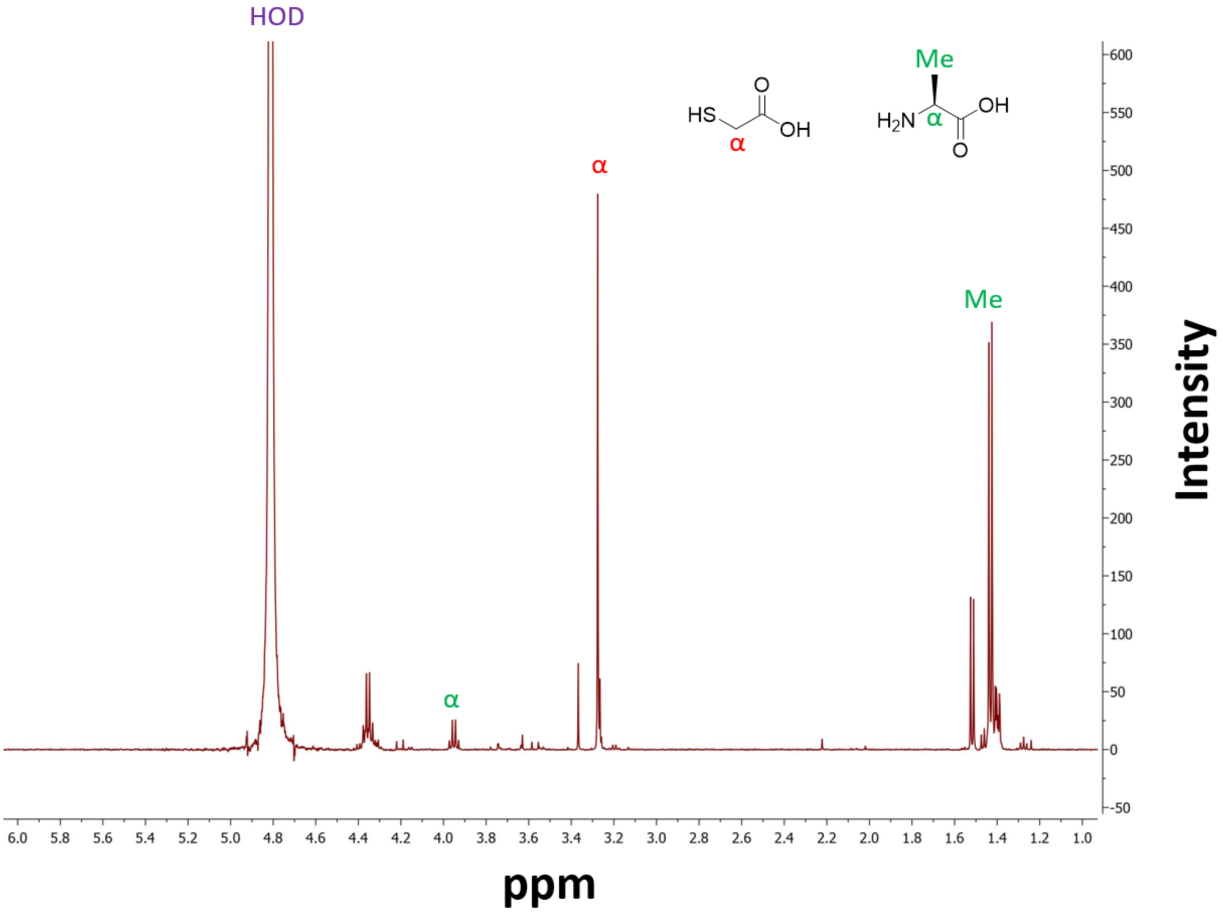

Supplementary Figure 22. Full spectrum of a dried mixture of tg plus Ala mixture. A mixture of tg and Ala was dried-down at a 5:1 molar ratio (tg:Ala) at 65 ˚C for six days. The dried mixture was resuspended in a phosphate buffer in D_2O (pH 2.5). The free α-proton chemical shift of Ala is centered at 3.95 parts per million (ppm). Upon dry-down of Ala with tg, the α-proton resonance shifts down-field to ~4.35 ppm. Integration of the free, un-reacted α–proton resonance of Ala indicated that 82% of Ala was converted into oligomers after 1 week at 65 °C.

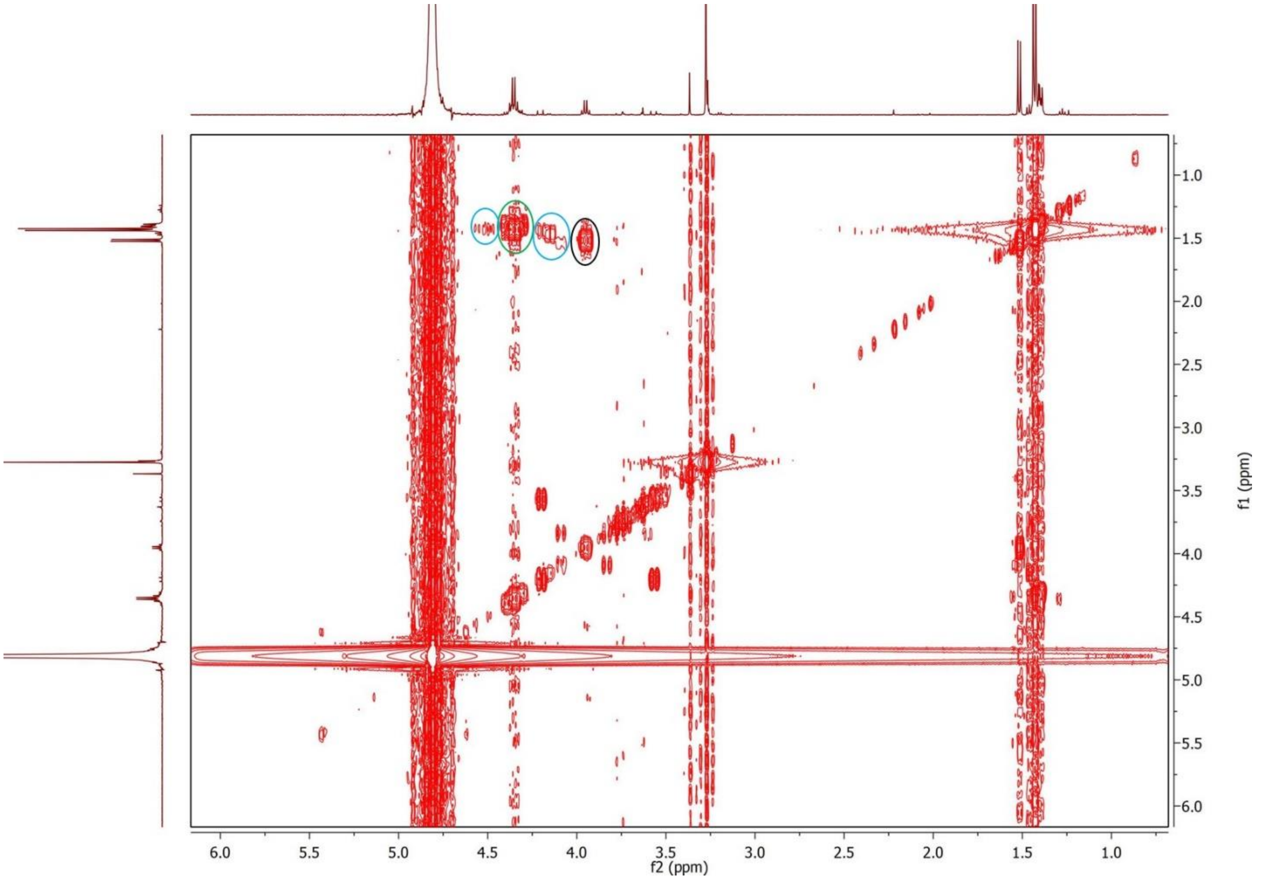

Supplementary Figure 23. ^{1}H-1H COSY NMR spectra of dry-down reactions of tg and Ala. A mixture of tg and Ala was dried in a 5:1 molar ratio (tg:Ala) at 65 ˚C for seven days and the resulting product mixture was resuspended in D_2O and analyzed by ^{1}H-^{1}H COSY for resonance assignments. The α-proton correlates to the methylene protons and in free Ala has a chemical shift of 3.95 ppm. The chemical shift of the α-proton shifts down-field upon reactivity through either its carboxylic side or amine. Cross peaks of interest are circled: black - correlation between the α-proton and methylene protons of non-reacted Ala; green - correlation between α-amidated protons of Ala and their corresponding methylene protons; blue – two envelopes are shown of reacted Ala species in which correlation between the α-protons and their corresponding methylene protons of Ala are shown.

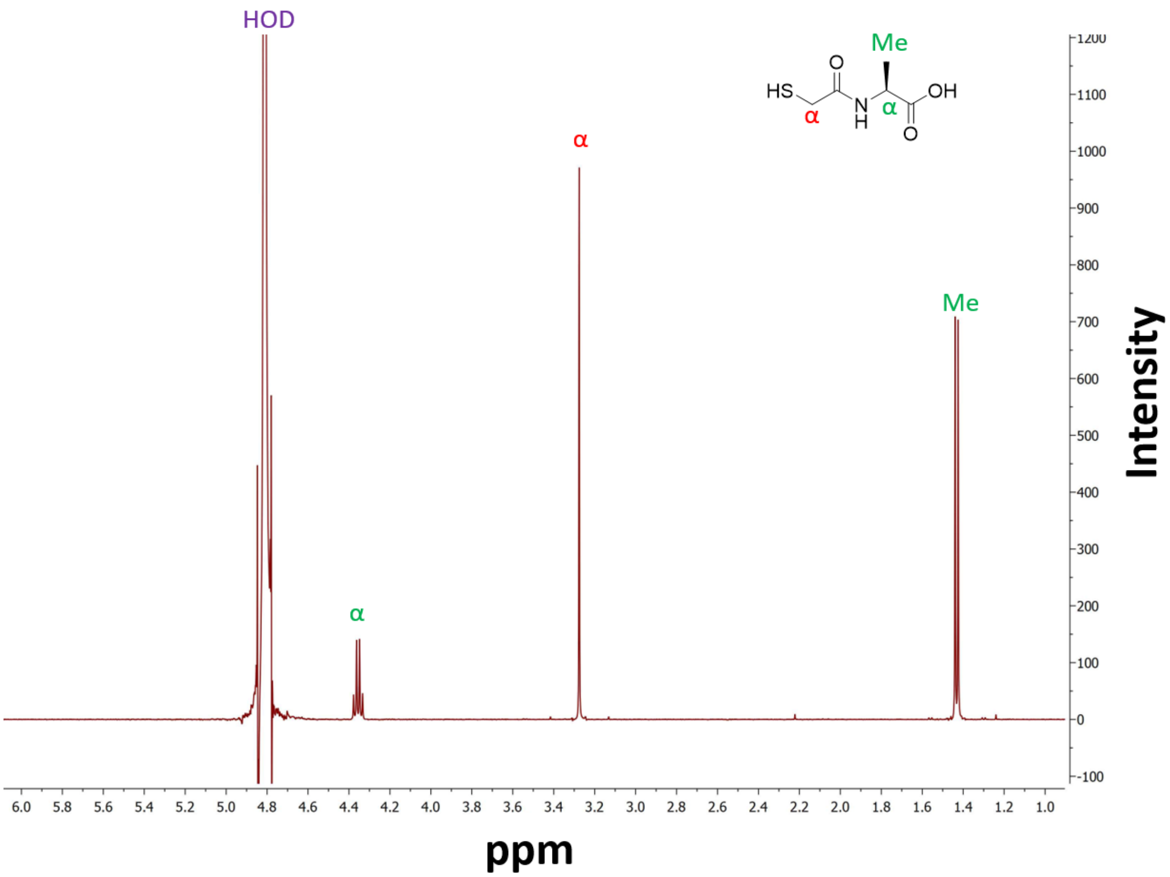

Supplementary Figure 24. Full spectrum of the tgA standard. A synthesized standard that is composed of a terminal tg linked with an amide to Ala, herein termed tgA, was resuspended in a phosphate buffer in D_2O (pH 2.5). The α-proton chemical shift of the Ala moiety is centered at 4.35 parts per million (ppm).

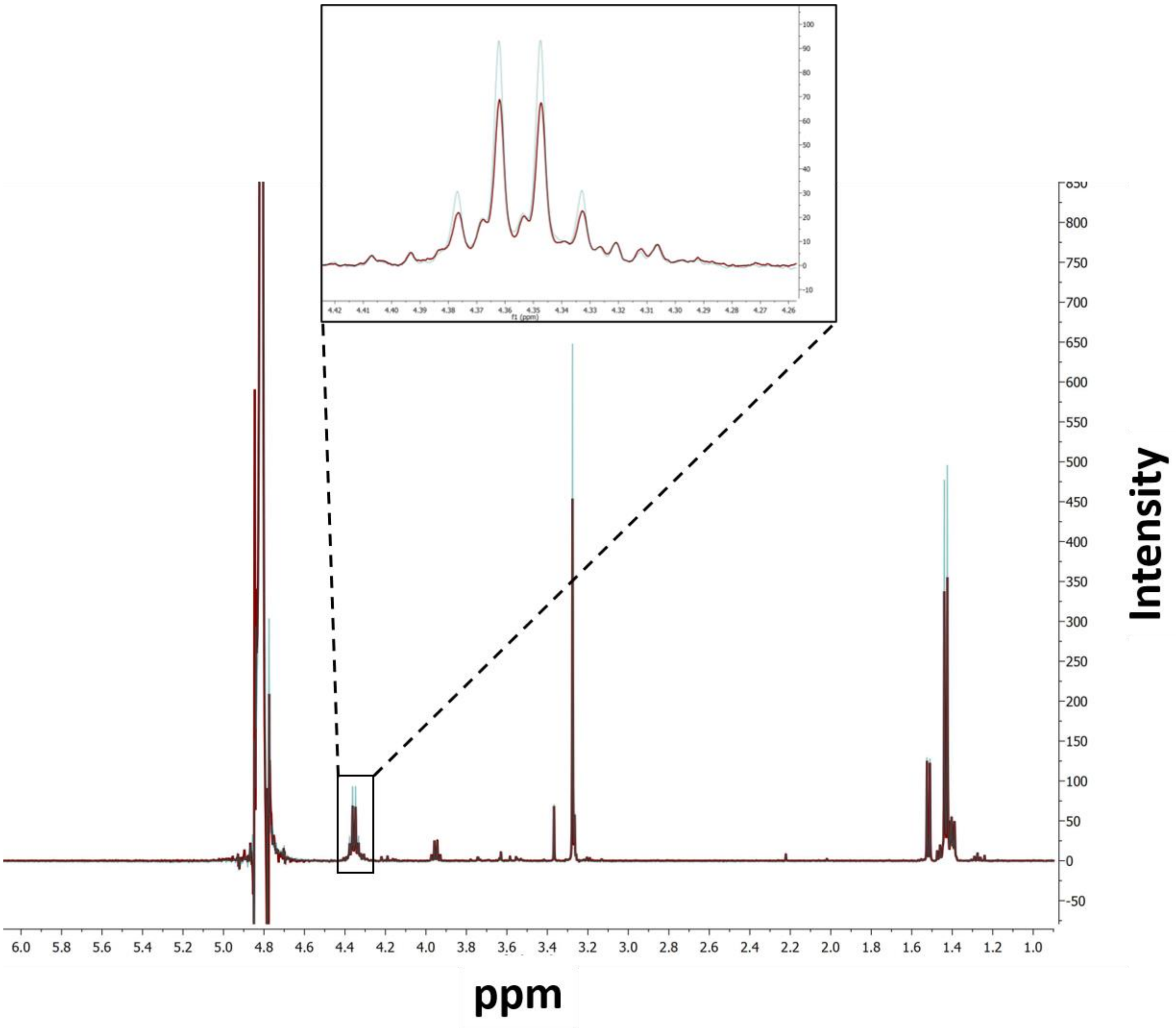

Supplementary Figure 25. Validation of the formation of tgA peptide upon dry-down of tg with Ala. A mixture of tg and Ala was dried at a 5:1 molar ratio (tg:Ala) at 65 ˚C for seven days. ^{1}H NMR spectra in a phosphate buffer in D_2O (pH 2.5) is shown before (*red* trace) and after (*turquoise* trace) spiking of a tgA standard into the dried mixture, supporting the formation of tgA upon drying of tg and Ala.

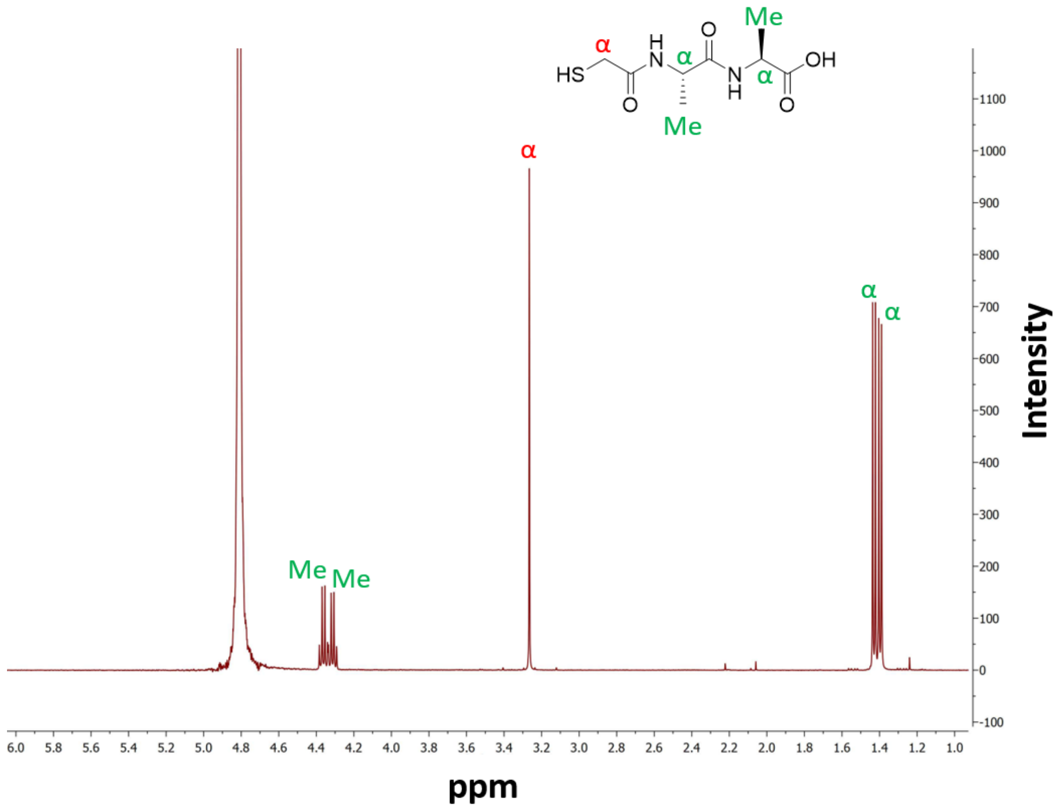

Supplementary Figure 26. Full spectrum of the tgAA standard. A synthesized standard that is composed of a terminal tg linked with two consecutive amides to Ala, herein termed tgAA, was resuspended in a phosphate buffer in D_2O (pH 2.5). The α-protons chemical shift of the Ala moieties are centered at 4.34 parts per million (ppm).

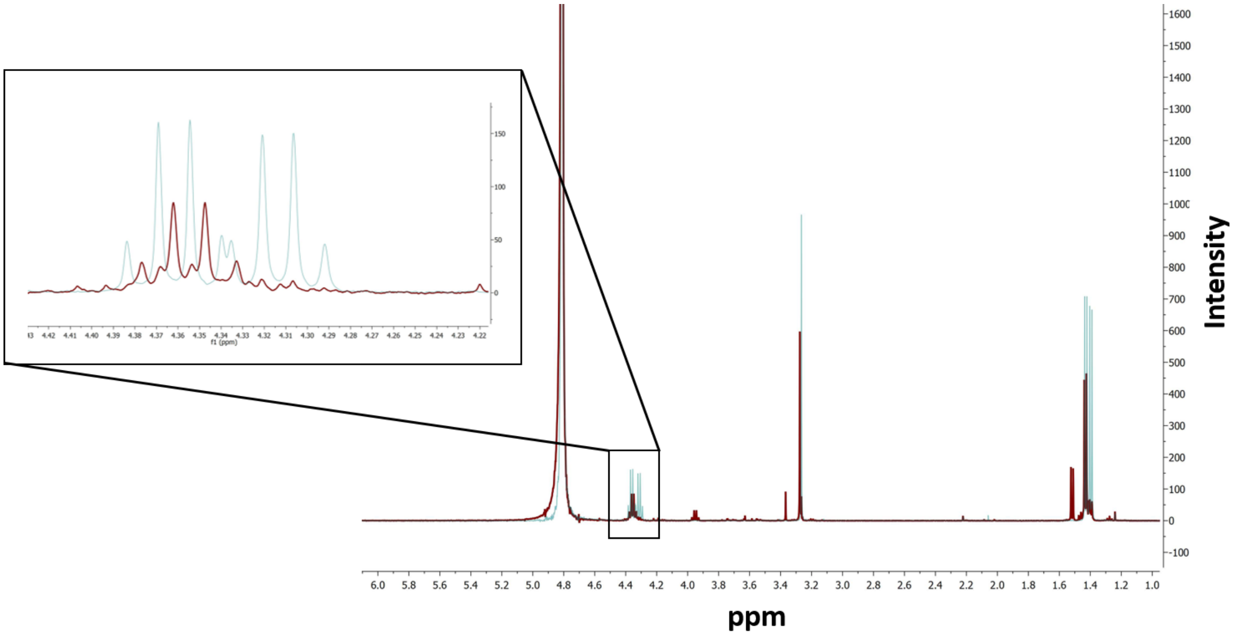

Supplementary Figure 27. Validation of the formation of tgAA peptide upon dry-down of tg with Ala. A mixture of tg and Ala was dried at a 5:1 molar ratio (tg:Ala) at 65 ˚C for seven days. ^{1}H NMR spectra in a phosphate buffer in D_2O (pH 2.5) is shown before (*red* trace) and after (*turquoise* trace) spiking of a tgAA standard into the dried mixture, supporting the formation of tgAA upon drying of tg and Ala.

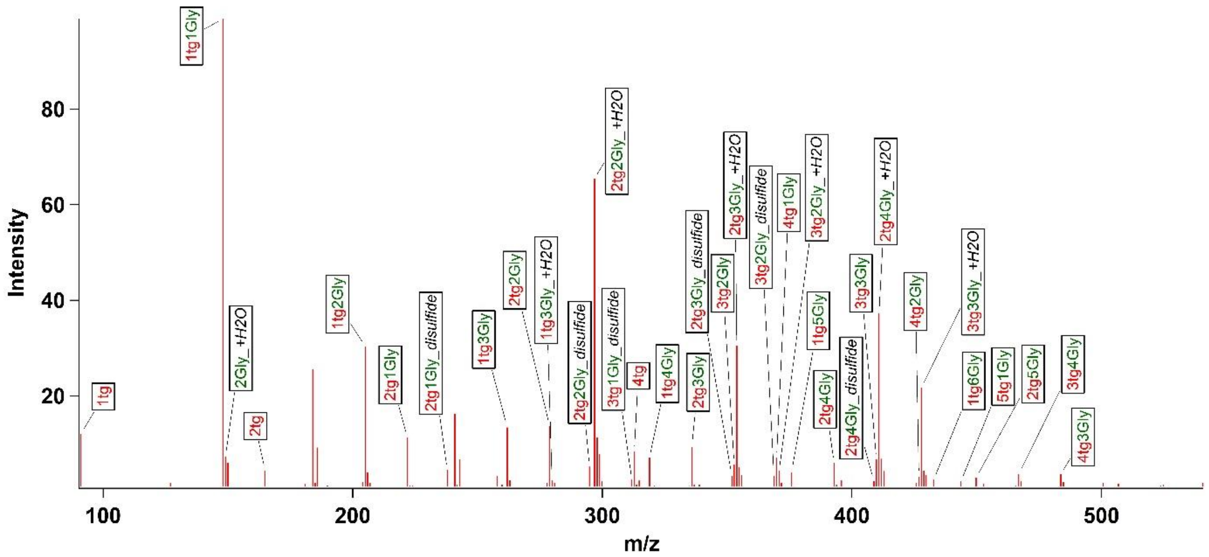

Supplementary Figure 28. ESI-MS of a dry-down reaction of tg and Gly at a 5:1 molar ratio supports the formation of thiodepsipeptides. tg and Gly were dried in a 5:1 molar ratio (tg:Gly) at 65 ˚C for seven days and the resulting products were analyzed by negative-mode ESI-MS, indicating a variety of thiodepsipeptides. tg is labeled in red, Gly is labeled in green. Species that are labelled with H_2O correspond to non-covalent adducts. All labeled species correspond to $[M-H]^-$ ions.

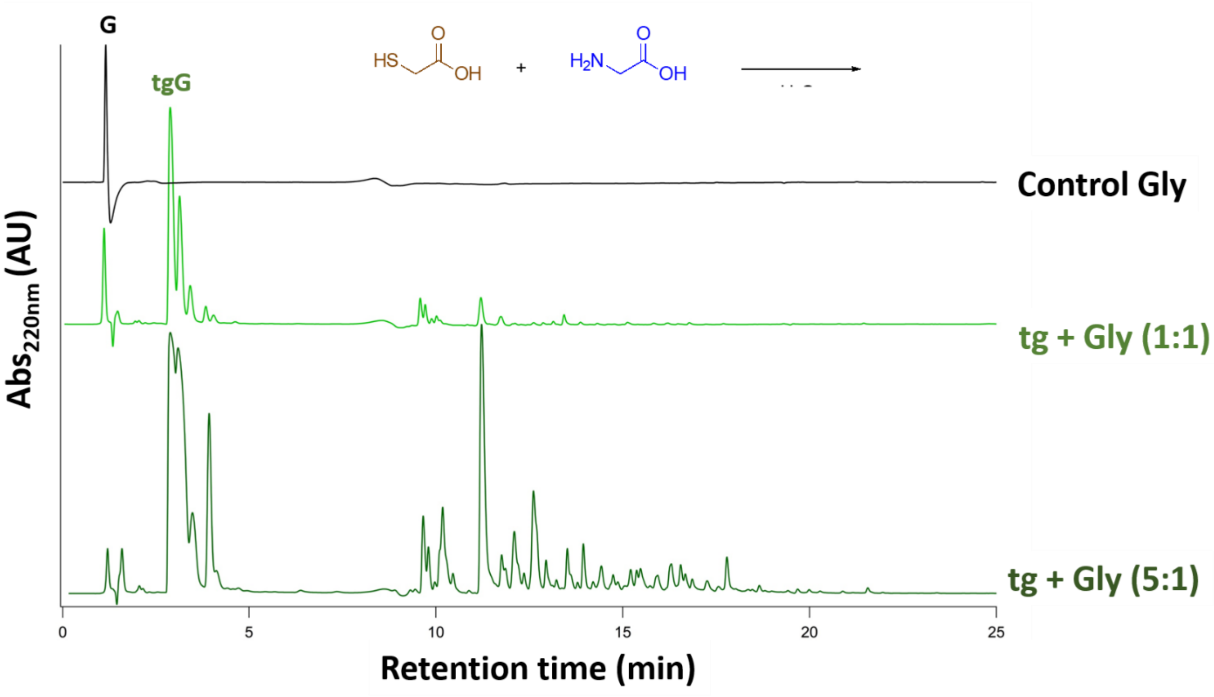

Supplementary Figure 29. HPLC of a dry-down reaction of tg and Gly at a 5:1, 1:1 molar ratio or Gly control supports the formation of thiodepsipeptides. tg and glycine (Gly) were dried at a 5:1 molar ratio (tg:Gly) or 1:1 molar ratio at 65 ˚C for seven days and the resulting products were analyzed by C18-HPLC, indicating a variety of thiodepsipeptides formed under the evaporative conditions. Control reaction that involved drying of Gly alone in the absence of the mercaptoacid did not result in formation of polymers, as expected.

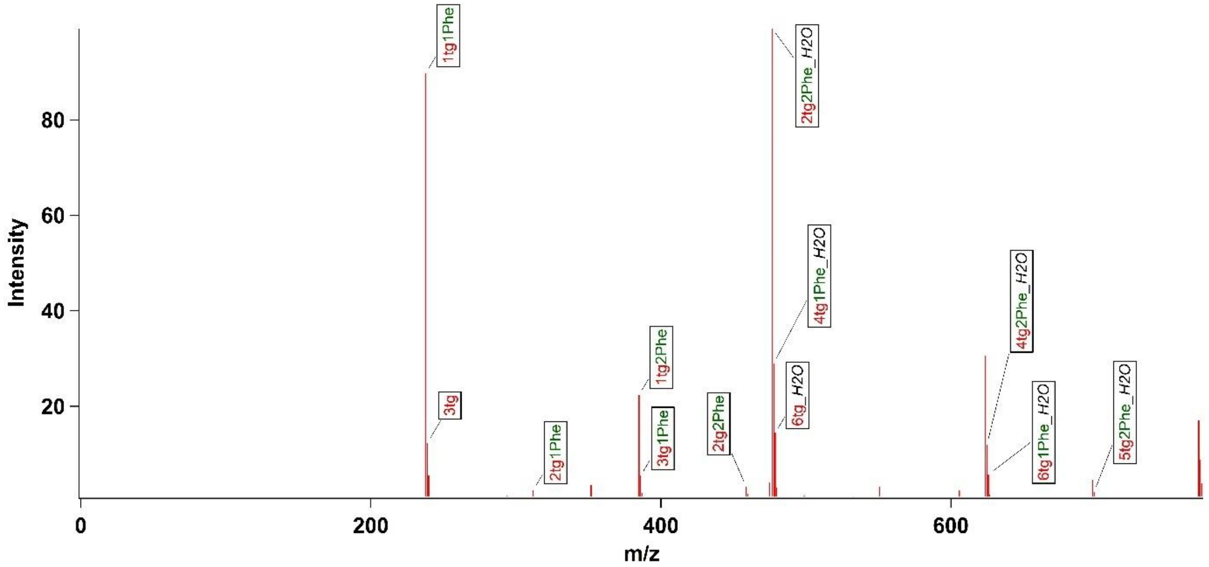

Supplementary Figure 30. ESI-MS of a dry-down reaction of tg and L-Phe at a 5:1 molar ratio supports the formation of thiodepsipeptides. tg and phenylalanine (Phe) were dried at a 5:1 molar ratio (tg:Phe) at 65 ˚C for seven days and the resulting products were analyzed by negative-mode ESI-MS, indicating a variety of thiodepsipeptides. tg is labeled in red, Phe is labeled in green. Species that are labelled with H_2O correspond to non-covalent adducts. All labeled species correspond to $[M-H]^-$ ions.

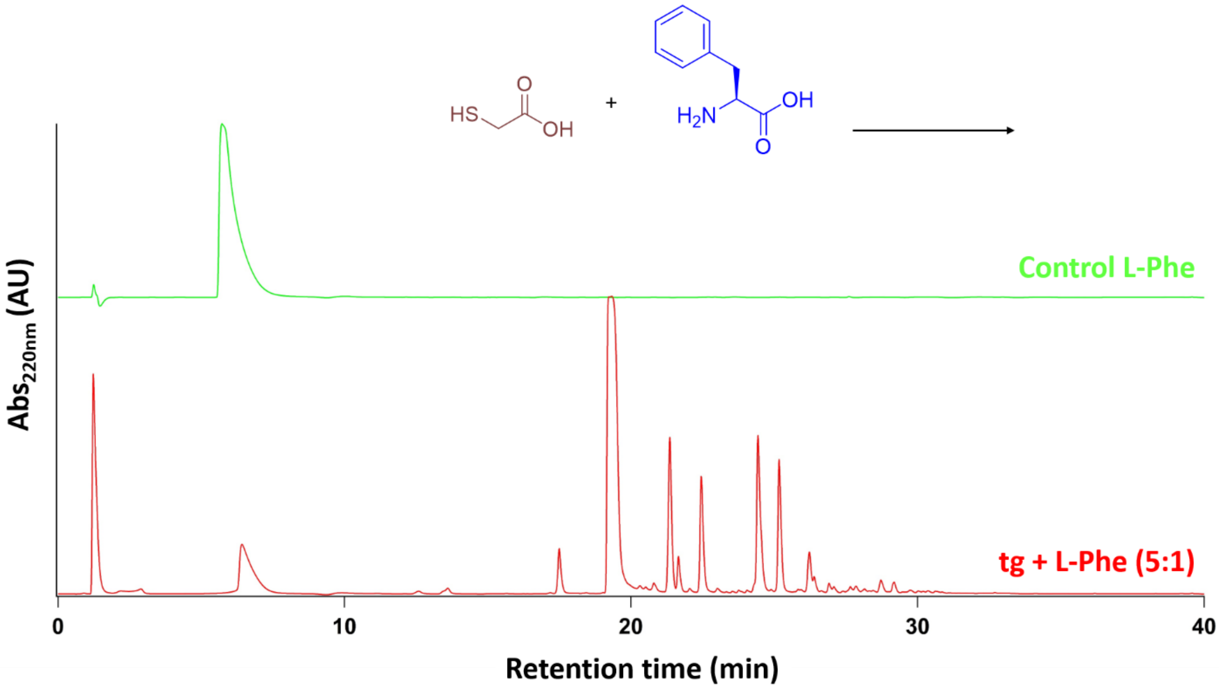

Supplementary Figure 31. HPLC of a dry-down reaction of tg and Phe supports the formation of thiodepsipeptides. tg and phenylalanine (Phe) were dried at a 5:1 molar ratio (tg:Phe) at 65 ˚C for seven days and the resulting products were analyzed by C18-HPLC, indicating a variety of thiodepsipeptides formed under the evaporative conditions. Control reaction that contained dry-down of Phe alone in the absence of the mercaptoacid did not result in formation of polymers, as expected.

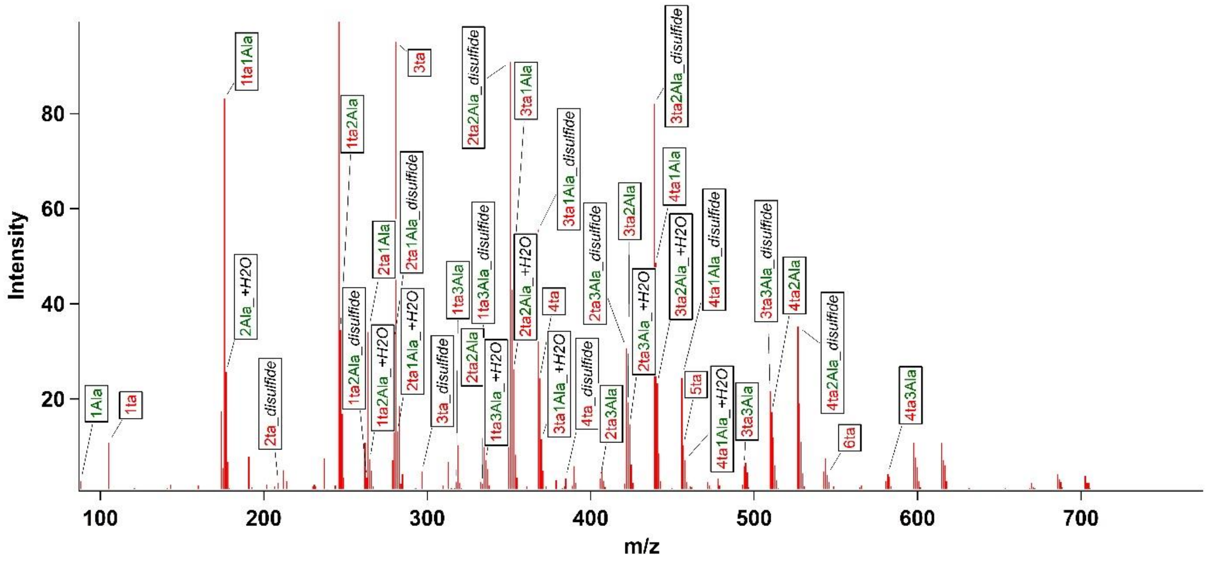

Supplementary Figure 32. ESI-MS of a dry-down reaction of ta and L-Ala at a 5:1 molar ratio supports the formation of thiodepsipeptides. Thiolactic acid (ta, racemic mixture) and L-Ala were dried at a 5:1 molar ratio (tg:Ala) at 65 ˚C for seven days and the resulting products were analyzed by negative-mode ESI-MS, indicating a variety of thiodepsipeptides. ta is labeled in red, Ala is labeled in green. Species that are labelled with H_2O correspond to non-covalent adducts. All labeled species correspond to $[M\text{-}H]^-$ ions.

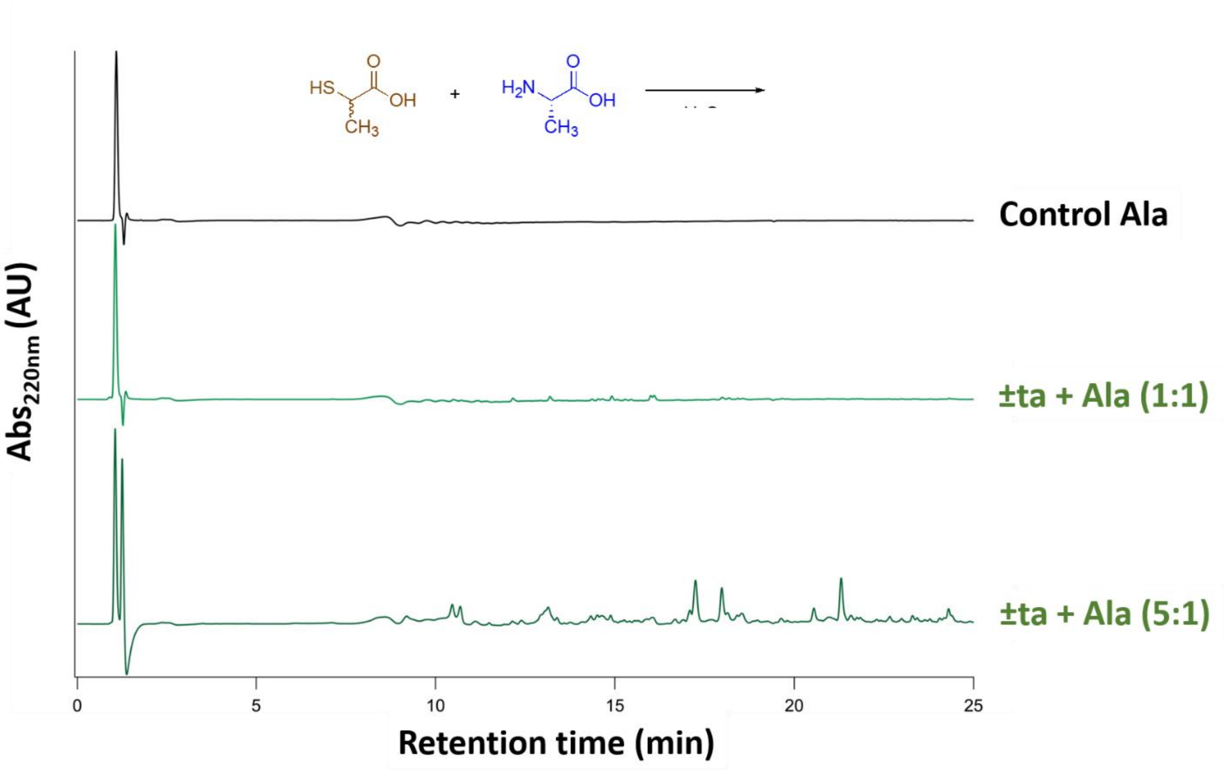

Supplementary Figure 33. HPLC of a dry-down reaction of ta and L-Ala at a 5:1, 1:1 molar ratio or Ala control supports the formation of thiodepsipeptides. Thiolactic acid (ta, racemic mixture) and L-Ala were dried at a 5:1 molar ratio (tg:Ala) or 1:1 molar ratio at 65 ˚C for seven days and the resulting products were analyzed by C18-HPLC, indicating a variety of thiodepsipeptides formed under the evaporative conditions. Control reaction that contained dry-down of Ala alone in the absence of the mercaptoacid did not result in formation of polymers, as expected.

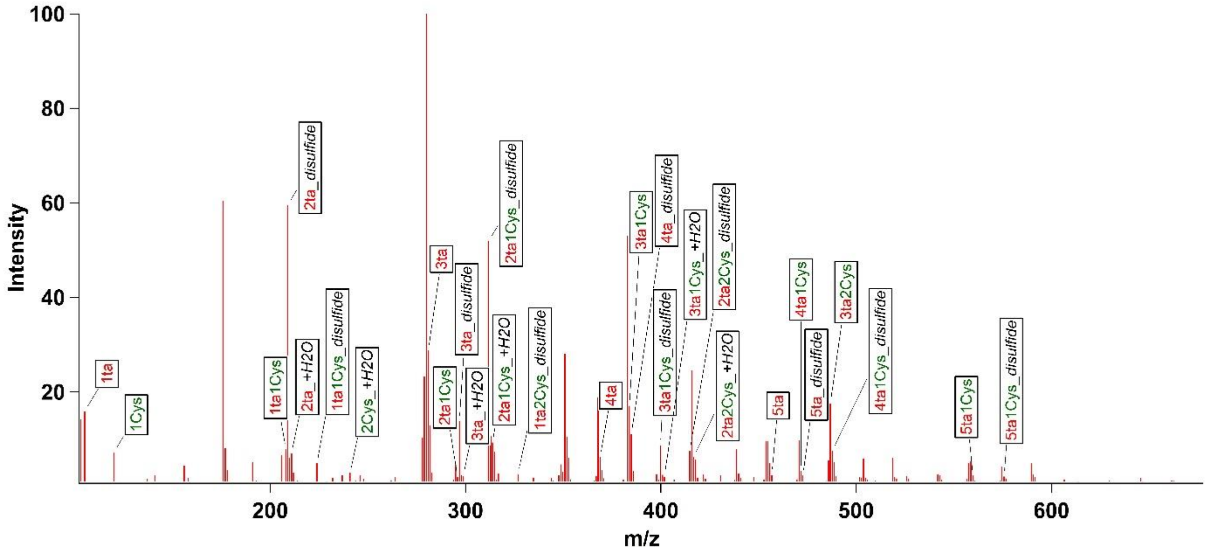

Supplementary Figure 34. ESI-MS of a dry-down reaction of ta and L-Cys at a 5:1 molar ratio supports the formation of thiodepsipeptides. Thiolactic acid (ta, racemic mixture) and L-Cys were dried at a 5:1 molar ratio (tg:Cys) at 65 ˚C for seven days and the resulting products were analyzed by negative-mode ESI-MS, indicating a variety of thiodepsipeptides. ta is labeled in red, Cys is labeled in green. Species that are labelled with H_2O correspond to non-covalent adducts. All labeled species correspond to $[M-H]^-$ ions.

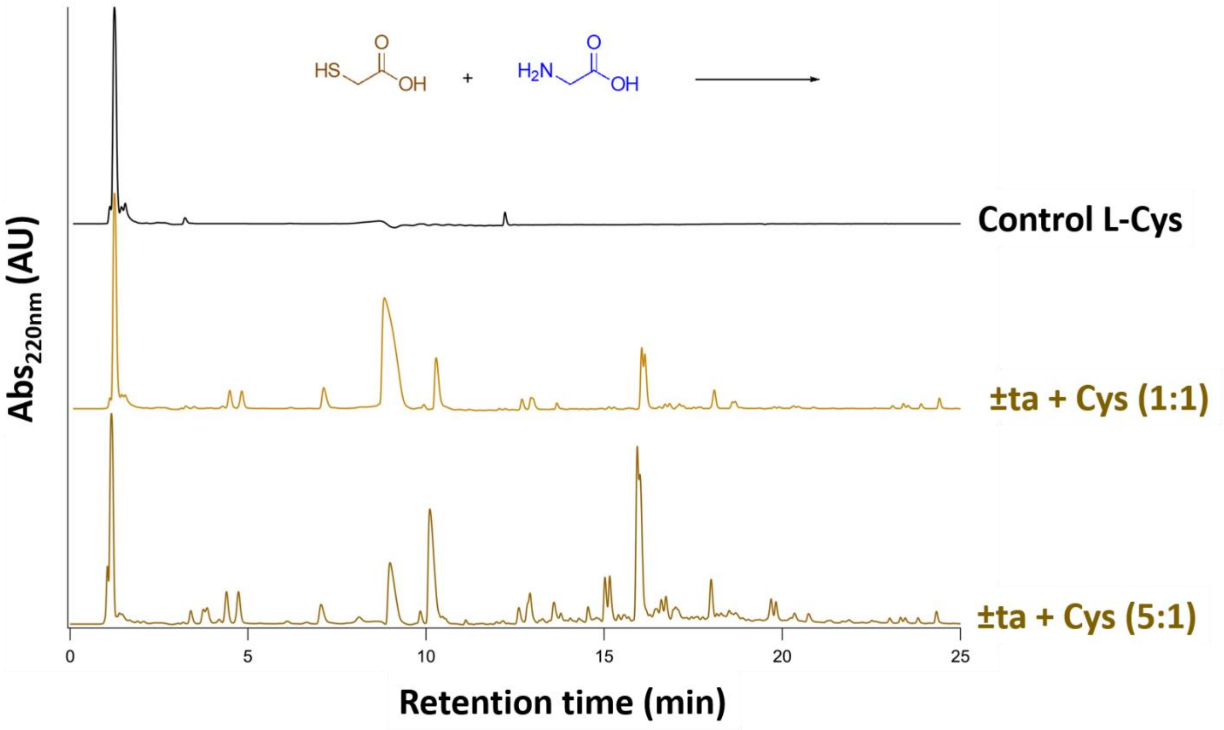

Supplementary Figure 35. HPLC of a dry-down reaction of ta and L-Cys at a 5:1, 1:1 molar ratio or Cys control supports the formation of thiodepsipeptides. Thiolactic acid (ta, racemic mixture) and L-Cys were dried at a 5:1 molar ratio (tg:Cys) or 1:1 molar ratio at 65 ˚C for seven days and the resulting products were analyzed by C18-HPLC, indicating a variety of thiodepsipeptides formed under the evaporative conditions. Control reaction that contained dry-down of Cys alone in the absence of the mercaptoacid did not result in formation of polymers, as expected.

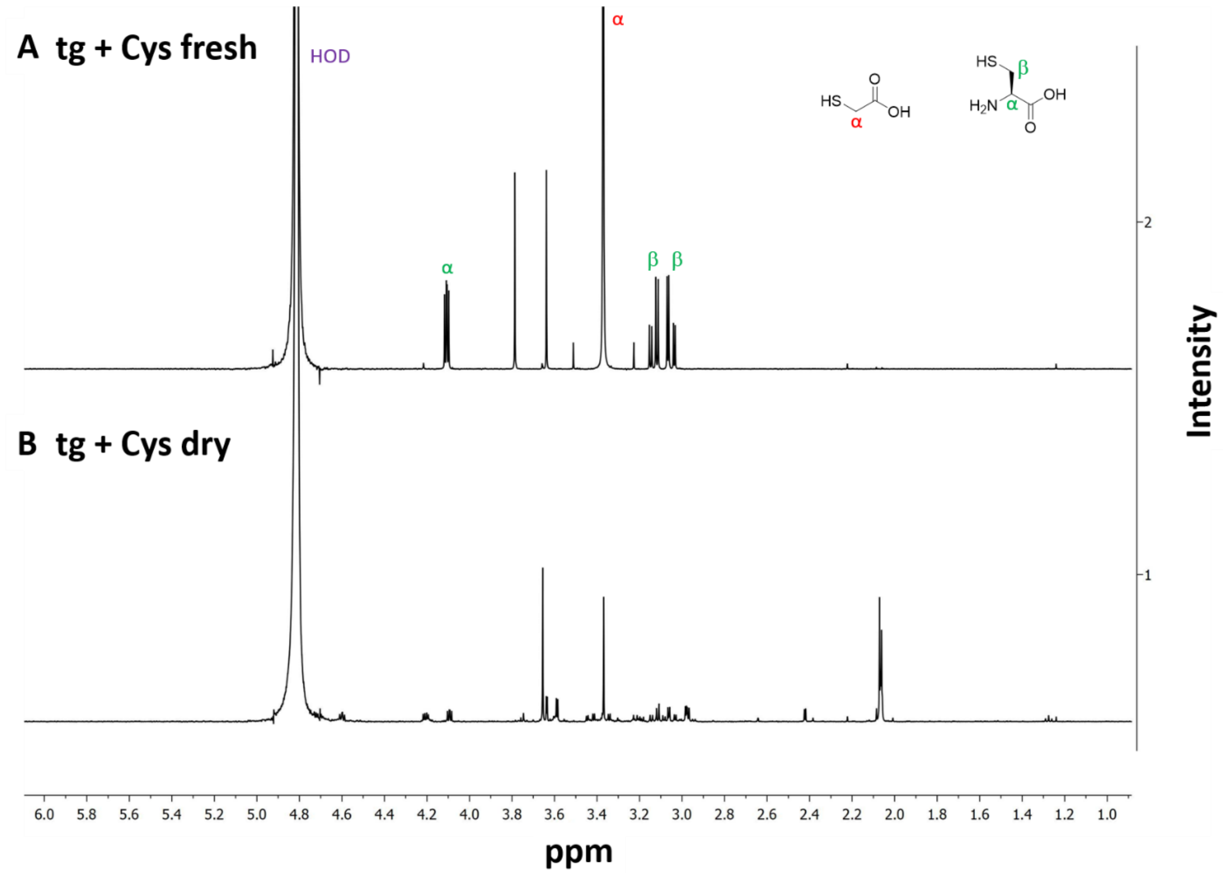

Supplementary Figure 36. ^{1}H NMR Spectrum supports the formation of oligomers in dry-down reactions of tg and Cys. ^{1}H NMR spectrum of a mixture of tg and cysteine (Cys) in D_2O before (A) and after (B) drying at 65 ˚C for seven days.

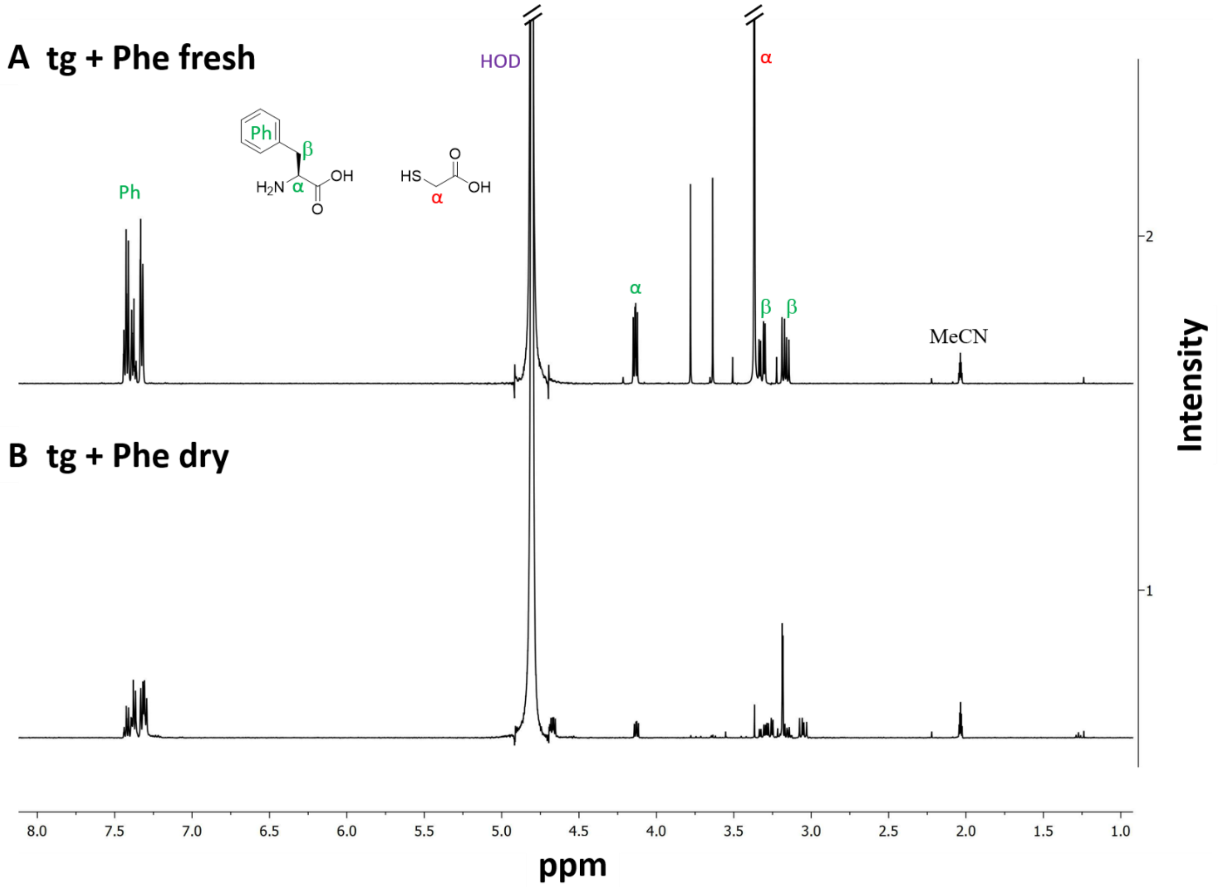

Supplementary Figure 37. ^{1}H NMR Spectrum supports the formation of oligomers in dry-down reactions of tg and Phe. ^{1}H NMR spectrum of a mixture of tg and phenylalanine (Phe) in D_2O/CD_3CN before (A) and after (B) drying at 65 ˚C for seven days. Internal integration of the remaining non-reacted α-proton of Phe to its phenyl protons indicated that about 66% of Phe has converted into oligomers.

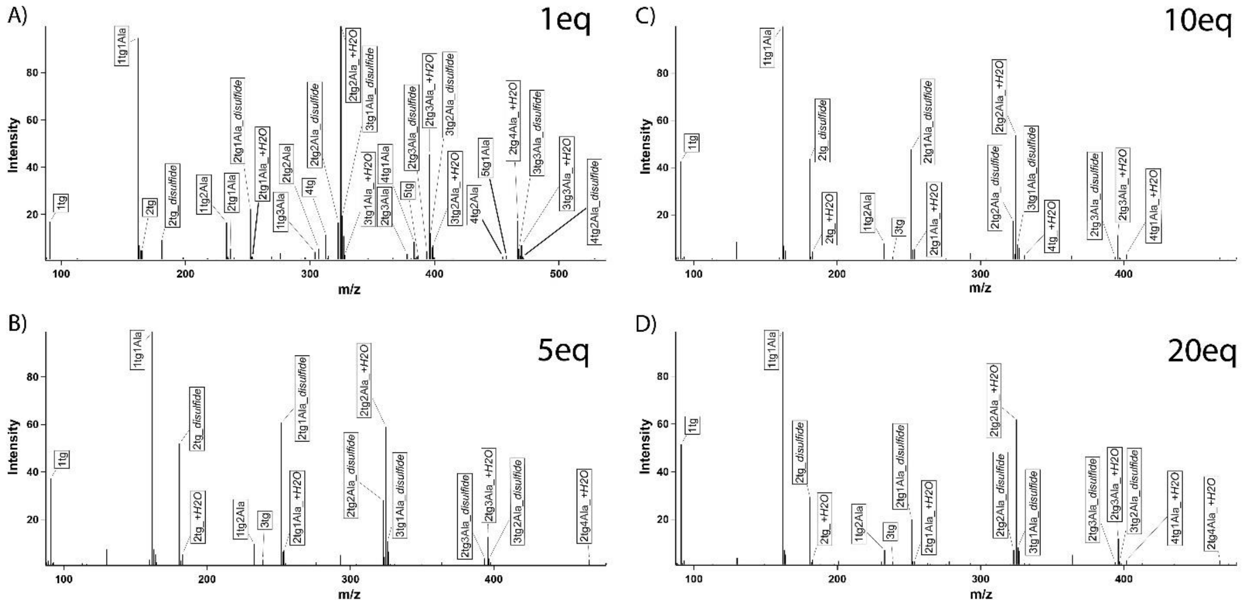

Supplementary Figure 38. Formation of thiodepsipeptides in dry-down reactions occurs over a wide range of pH conditions. tg and Ala were dried at a 5:1 molar ratio (tg:Ala) at 65 ˚C for seven days in the presence of imidazole at varying amounts. Different number of imidazole equivalents were added (referring to the amount of the amino acid): 1eq, 5eq, 10eq, and 20eq, which resulted in pH ranges of ~3.5, ~5.5, ~6.5, and ~7.0, respectively. The resulting products were analyzed by negative-mode ESI-MS, indicating a variety of thiodepsipeptides formed under all pH conditions. All labeled species correspond to $[M-H]^-$ ions. Species that are labelled with H_2O correspond to non-covalent adducts.

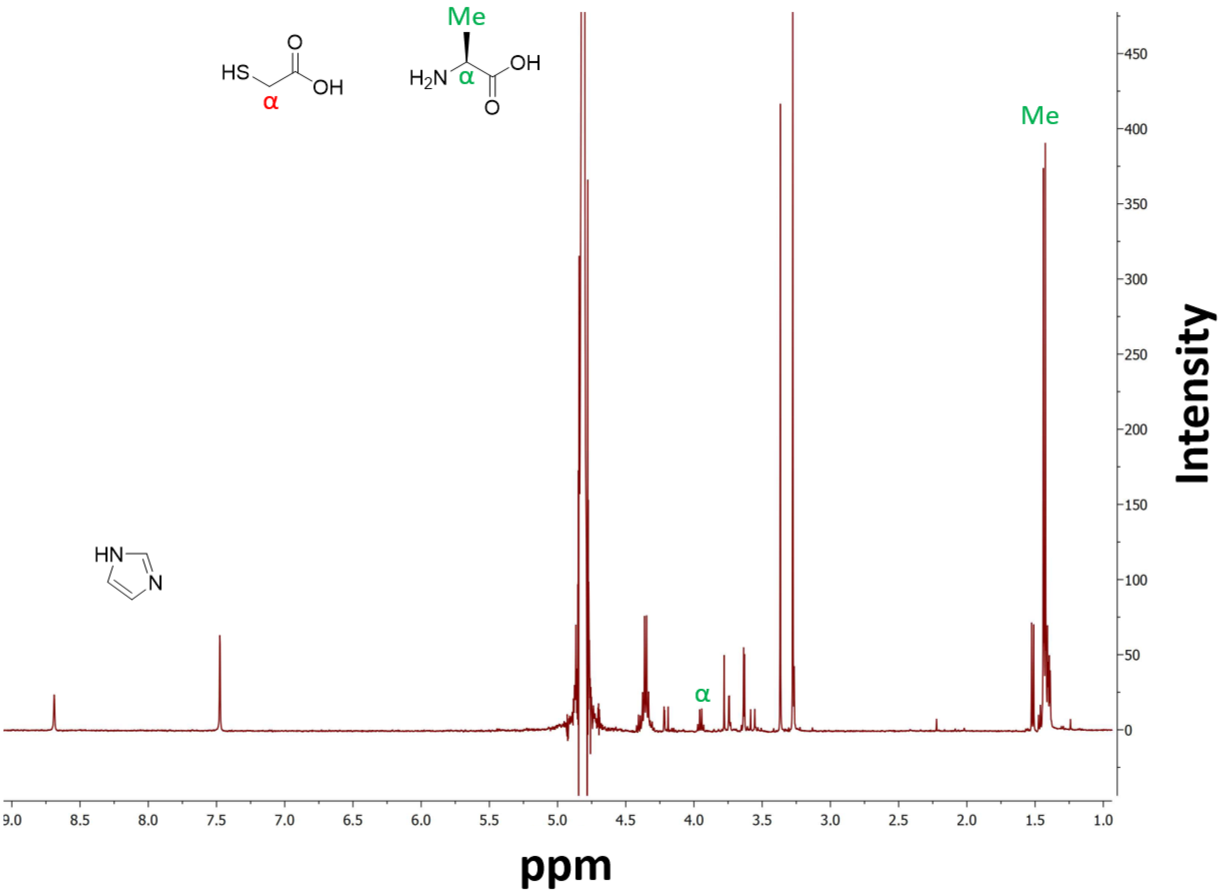

Supplementary Figure 39. ^{1}H NMR Spectrum of the product mixture from dry-down reactions of tg and Ala with 1 eq of imidazole. ^{1}H NMR spectrum of a mixture of tg and Ala in D_2O after dry-down at 65 ˚C for seven days in the presence of 1 equivalent of imidazole (referring to the amount of the amino acid). Integration of the free, un-reacted α–proton resonance of Ala indicated that 90% of Ala was converted into oligomers.

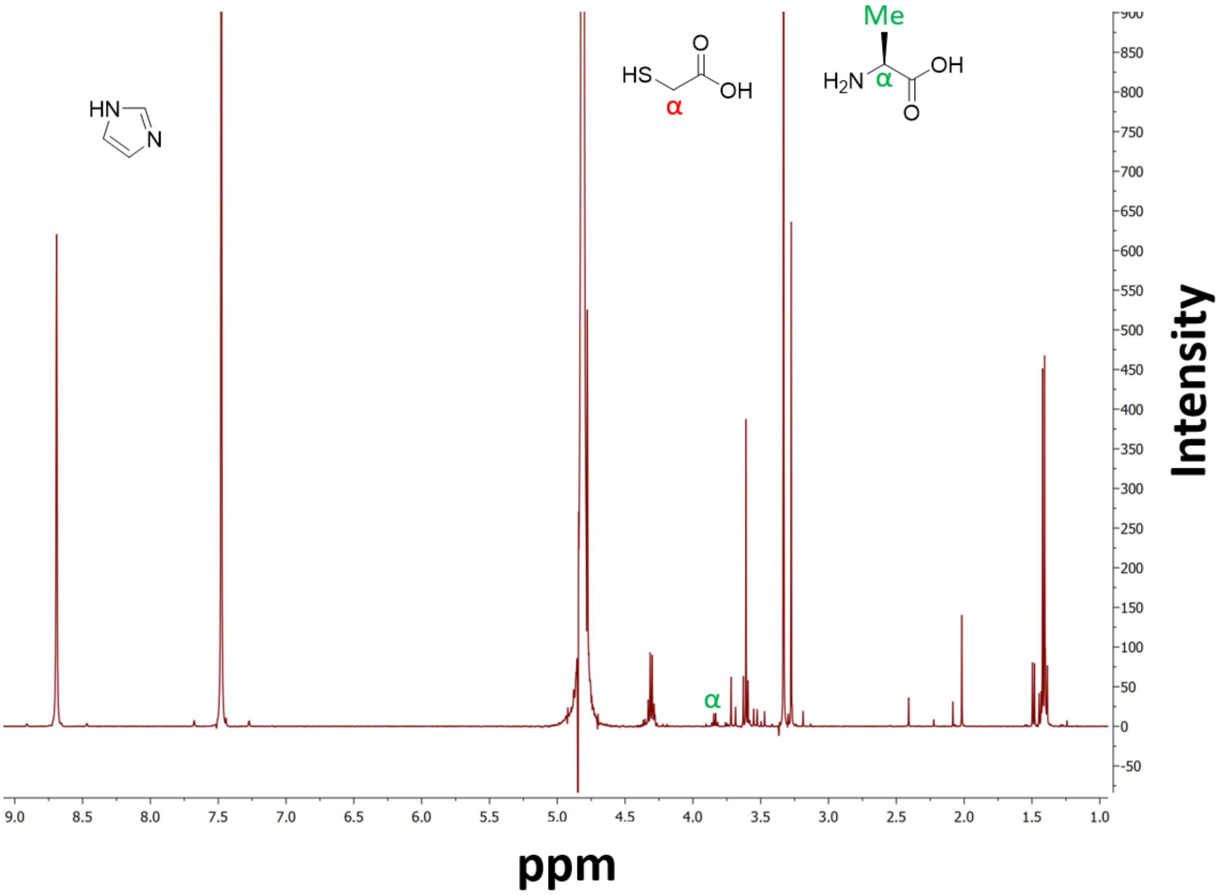

Supplementary Figure 40. ^{1}H NMR Spectrum of the product mixture from dry-down reactions of tg and Ala with 5 eq of imidazole. ^{1}H NMR spectrum of a mixture of tg and Ala in D_2O after dry-down at 65 ˚C for seven days in the presence of 5 equivalents of imidazole (referring to the amount of the amino acid). Integration of the free, un-reacted α–proton resonance of Ala indicated that 89% of Ala was converted into oligomers.

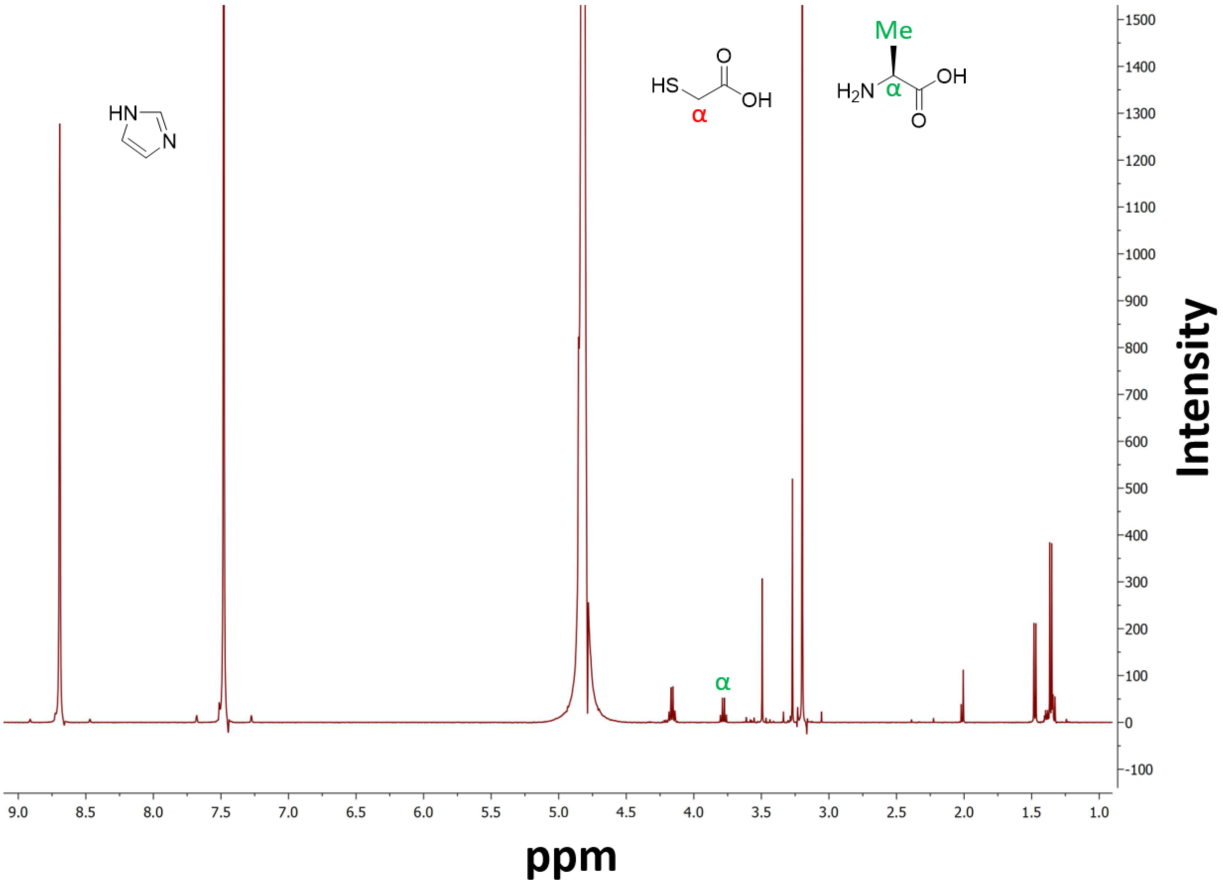

Supplementary Figure 41. ^{1}H NMR Spectrum of the product mixture from dry-down reactions of tg and Ala with 10 eq of imidazole. ^{1}H NMR spectrum of a mixture of tg and Ala in D_2O after dry-down at 65 ˚C for seven days in the presence of 10 equivalents of imidazole (referring to the amount of the amino acid). Integration of the free, un-reacted α–proton resonance of Ala indicated that 71% of Ala was converted into oligomers.

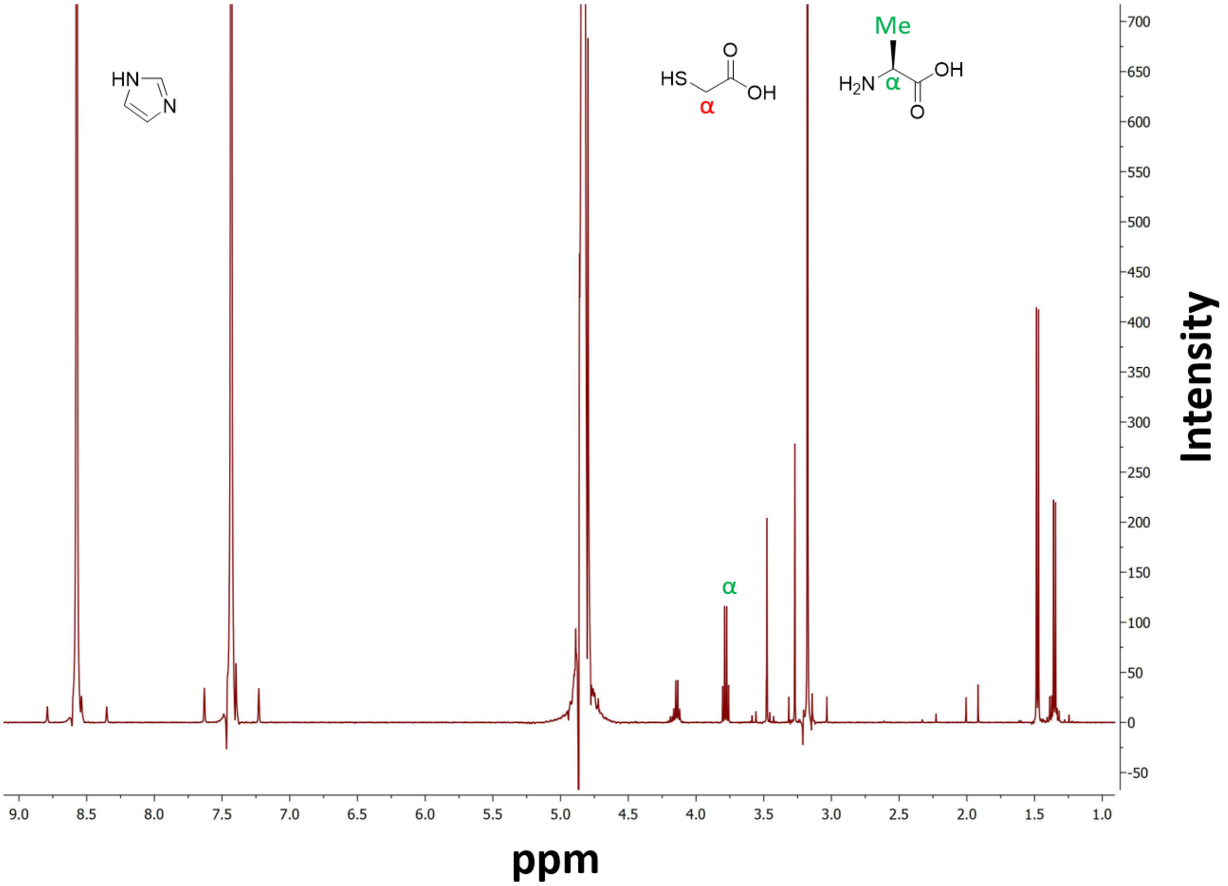

Supplementary Figure 42. ^{1}H NMR Spectrum of the product mixture from dry-down reactions of tg and Ala with 20 eq of imidazole. ^{1}H NMR spectrum of a mixture of tg and Ala in D_2O after dry-down at 65 ˚C for seven days in the presence of 20 equivalents of imidazole (referring to the amount of the amino acid). Integration of the free, un-reacted α–proton resonance of Ala indicated that 42% of Ala was converted into oligomers.

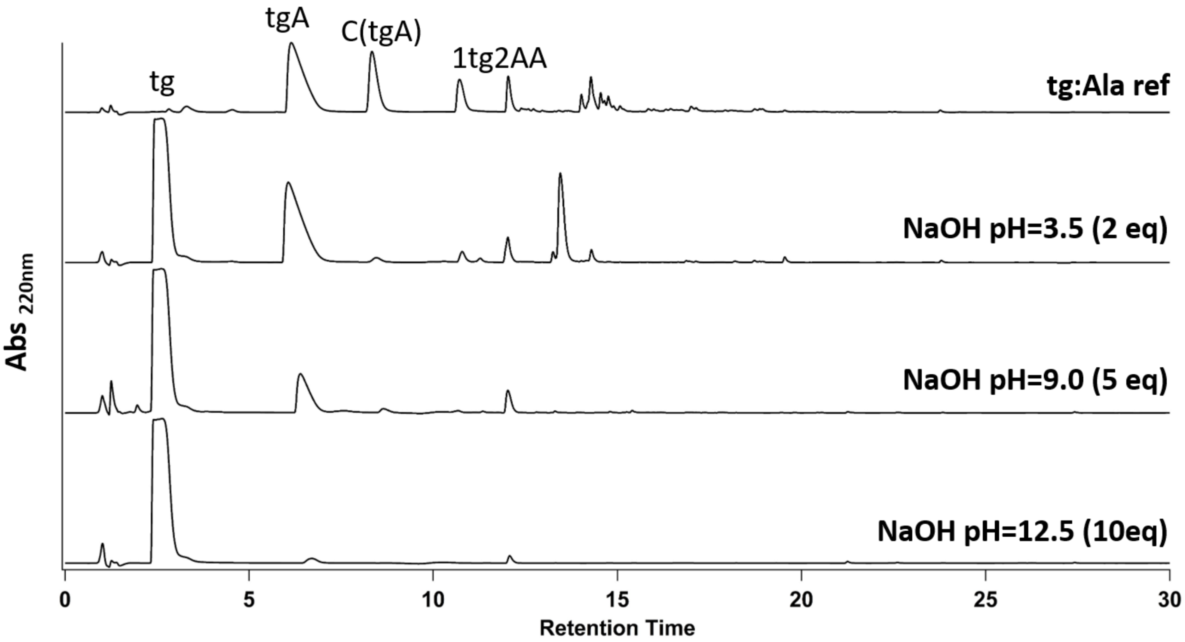

Supplementary Figure 43. Thiodepsipeptide formation occurs under a range of pH conditions. Chromatograms of products resulting from tg and Ala, in a 5:1 molar ratio, being dried at 65 °C for 1 week in various initial reaction pH. Two, 5, or 10 equivalents of NaOH were added with respect to the amount of amino acid to adjust the pH. Products are observed throughout the whole range of pH conditions tested, although the highest reactivity is observed at acidic pH.

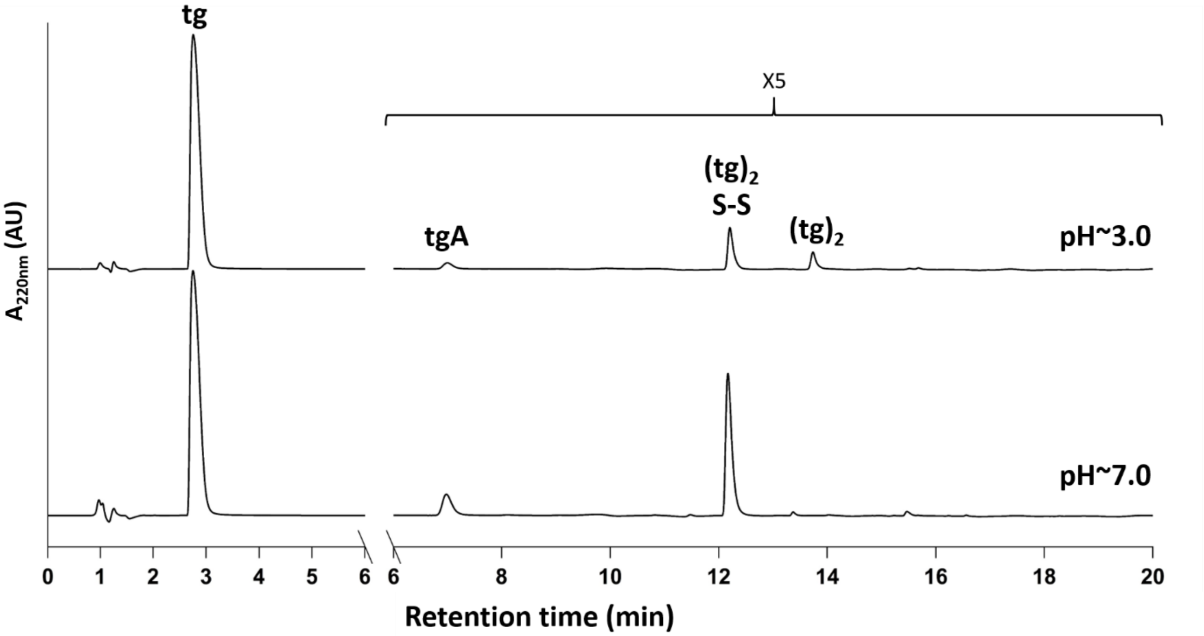

Supplementary Figure 44. Thiodepsipeptide formation occurs in aqueous solution. Chromatogram of products resulting from a mixture of tg and Ala in a 5:1 molar ratio being incubated in water under acidic conditions (*top*, pH~3.0, 55.55mM Ala and 277.75mM tg) or in the presence of sodium phosphate at pH 7.0 (*bottom*, 38.46mM Ala and 192.30mM tg) at 65 ˚C for seven days. Various product peaks are evident even in the wet phase (reactions in aqueous solution that were not dried-down), some of which contain amide bonds (such as tgA).

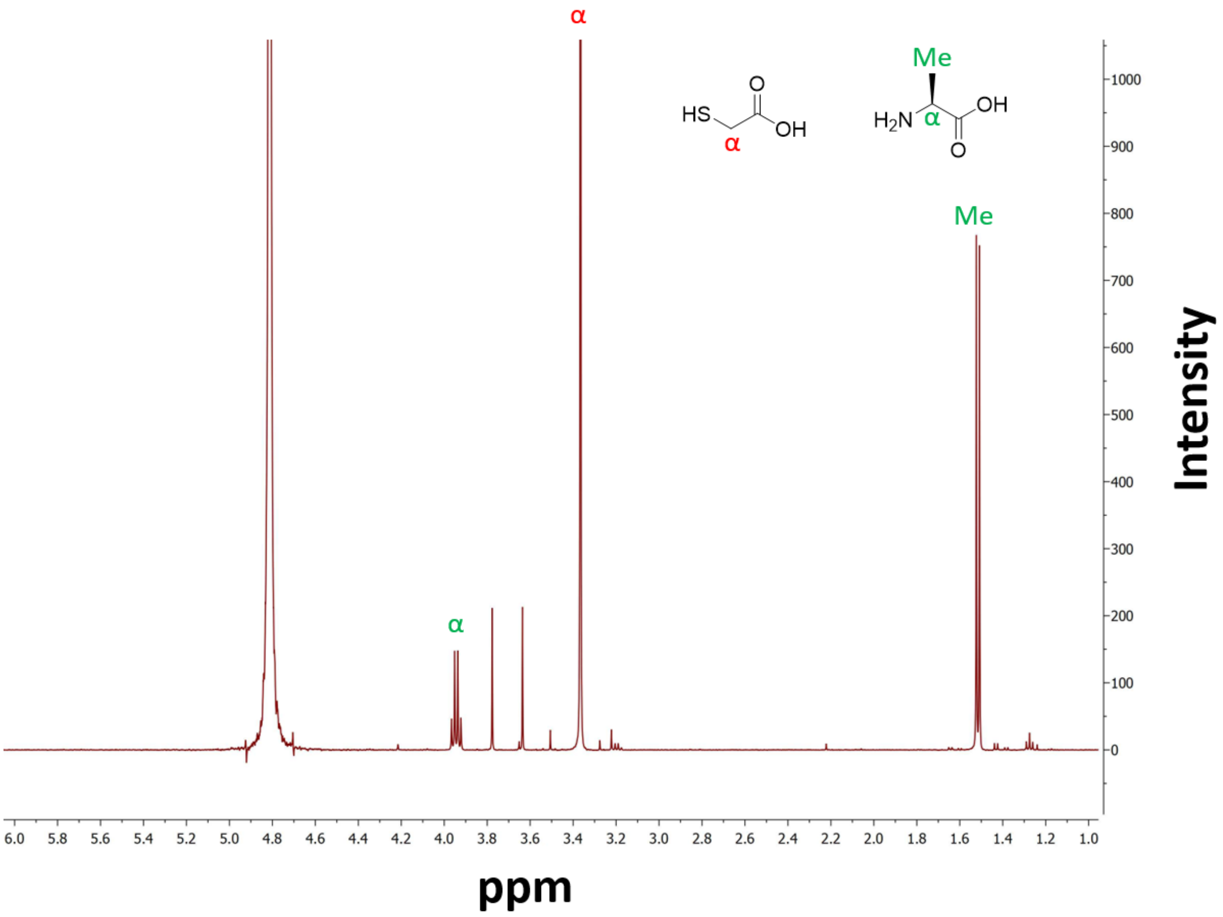

Supplementary Figure 45. ^{1}H NMR Spectrum of the product mixture from dry-down reactions of tg and Ala at 25 ˚C. ^{1}H NMR spectrum of a mixture of tg and Alanine (Ala) in D_2O after drying at 25 ˚C for seven days. Integration of the free, un-reacted α–proton resonance of Ala indicated that 4% of Ala was converted into oligomers.

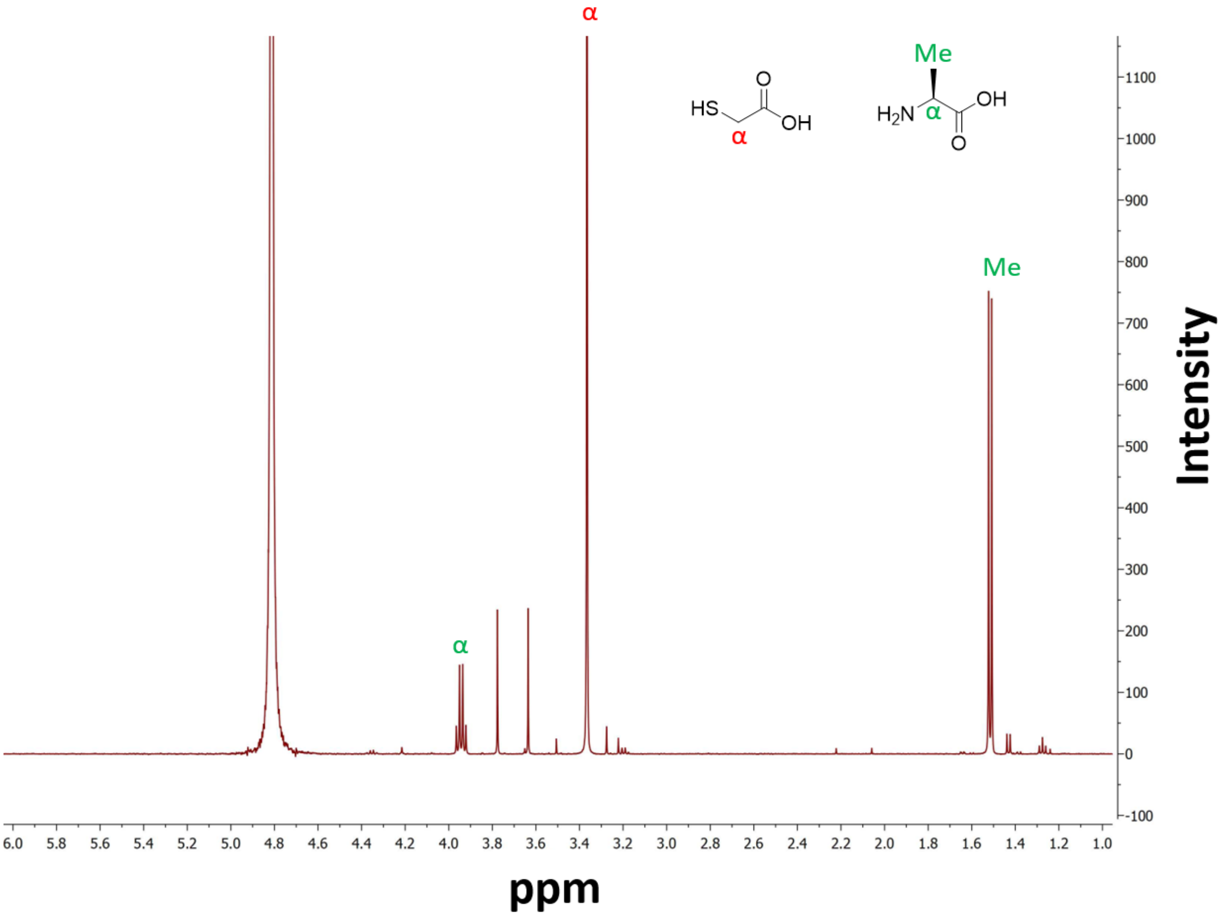

Supplementary Figure 46. ^{1}H NMR Spectrum of the product mixture from dry-down reactions of tg and Ala at 37 ˚C. ^{1}H NMR spectrum of a mixture of tg and Alanine (Ala) in D_2O after drying at 37 ˚C for seven days. Integration of the free, un-reacted α−proton resonance of Ala indicated that 6% of Ala was converted into oligomers.

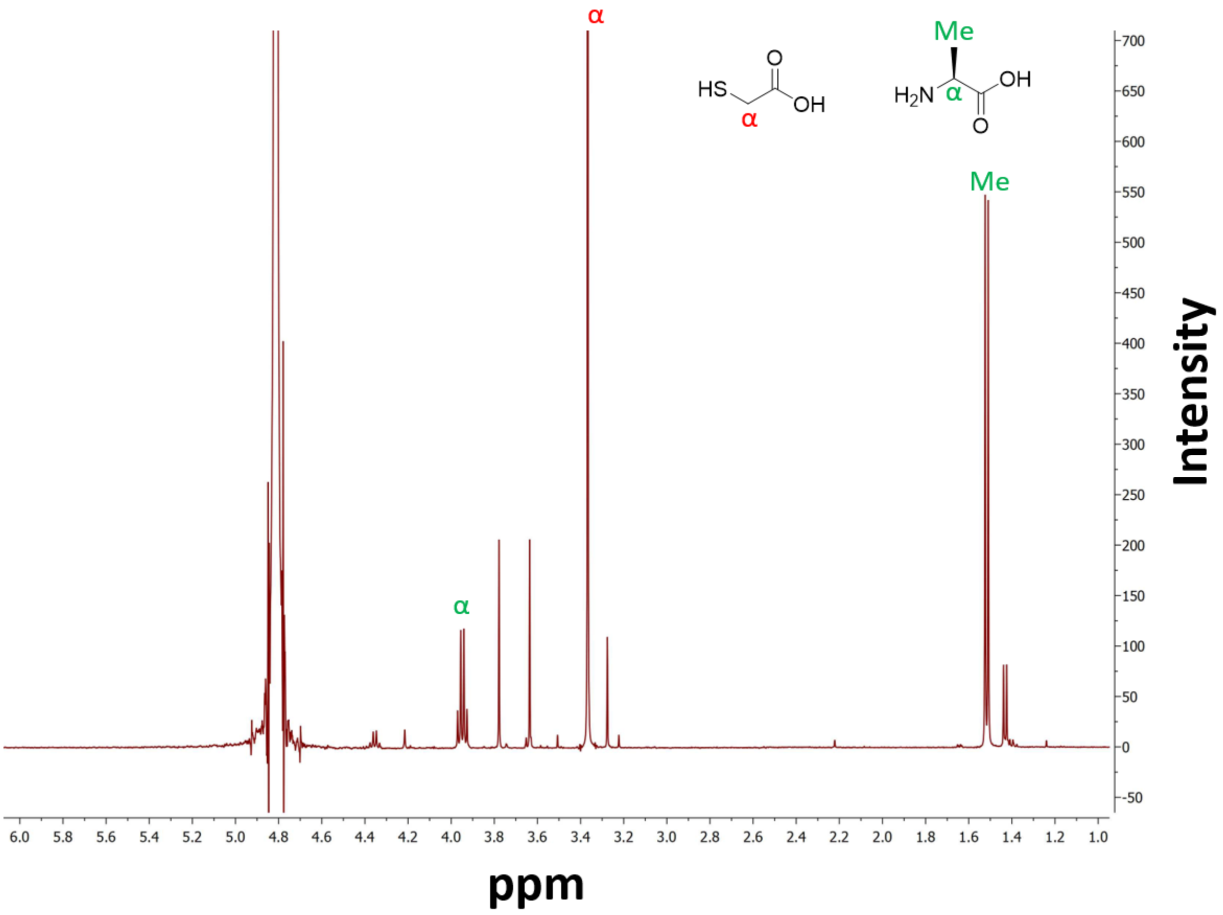

Supplementary Figure 47. ^{1}H NMR Spectrum of the product mixture from dry-down reactions of tg and Ala at 50 ˚C. ^{1}H NMR spectrum of a mixture of tg and Alanine (Ala) in D_2O after drying at 50 ˚C for seven days. Integration of the free, un-reacted α–proton resonance of Ala indicated that 23% of Ala was converted into oligomers.

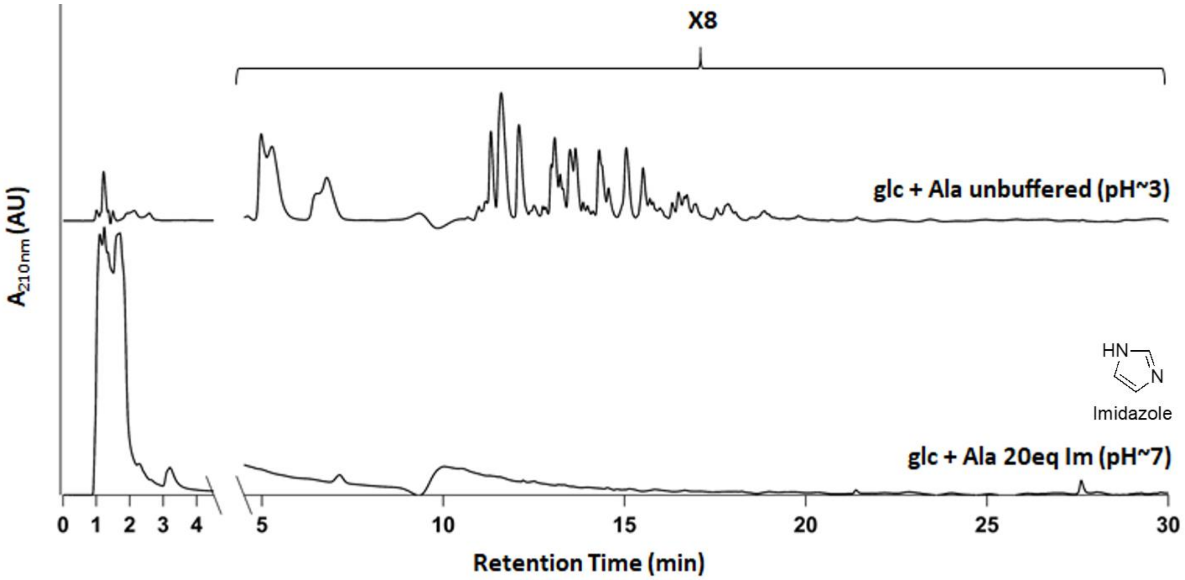

Supplementary Figure 48. Imidazole does not catalyze formation of depsipeptides upon drying of glycolic acid with Ala. Glycolic acid (glc) and L-Ala were dried at a 5:1 molar ratio (tg:Ala) at 65 ˚C in the absence (pH~3) or presence (pH~7.0) of 20 equivalents of imidazole (referring to the amount of Ala). The resulting products were analyzed by hydrophobicity-based separation using C18-HPLC, which shows that the addition of imidazole inhibited formation of depsipeptides.

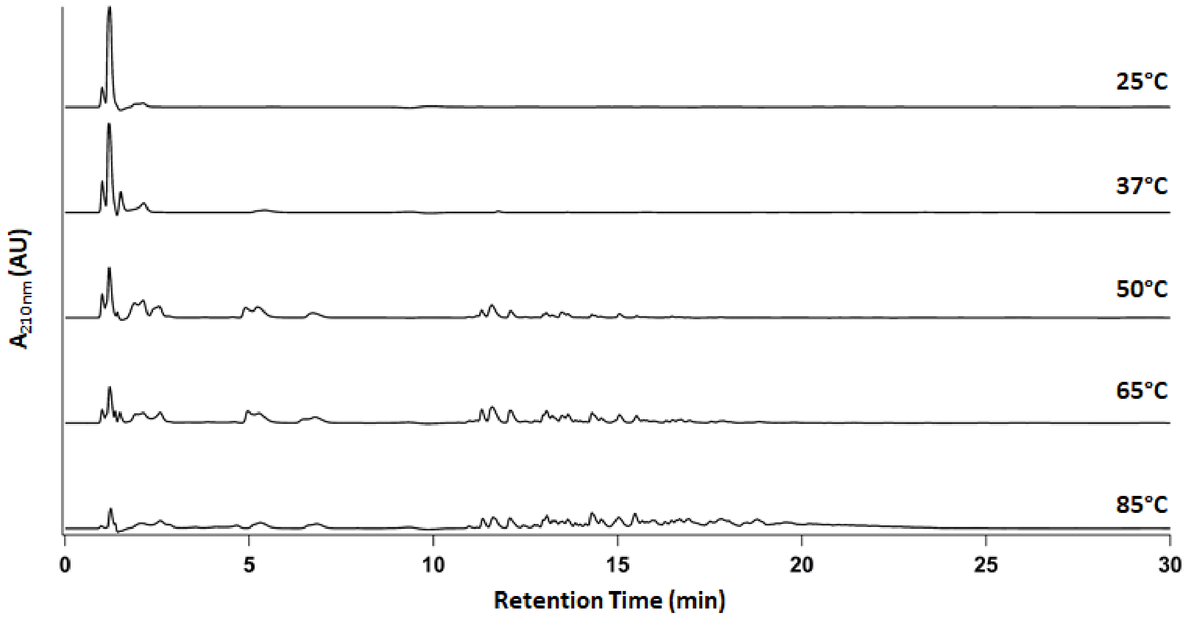

Supplementary Figure 49. Temperature-dependent formation of depsipeptides under dry-down reactions of glycolic acid and Ala. Glycolic acid (glc) and L-Ala were dried at a 5:1 molar ratio (tg:Ala) at varying temperature for seven days and the resulting products were analyzed by hydrophobicity-based separation using C18-HPLC. Only minuscule product formation is observed at 37 ˚C and none at 25 ˚C.

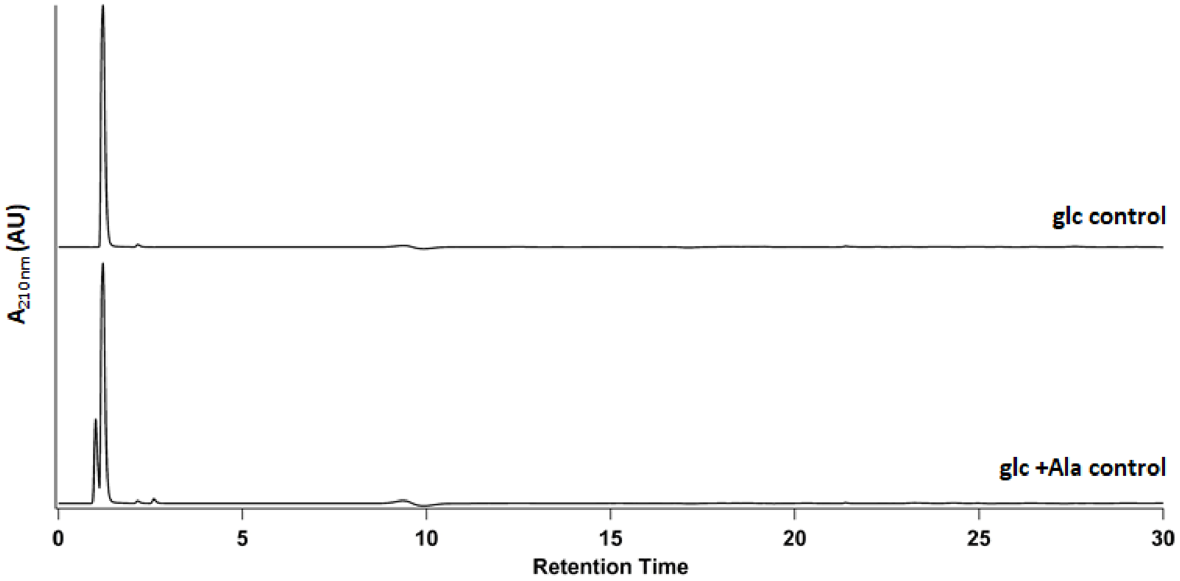

Supplementary Figure 50. Incubation of glycolic acid or a mixture of glycolic acid and Ala in water does not result in formation of polyesters or depsipeptides. Glycolic acid (glc) or a mixture of glc with L-Ala at a 5:1 molar ratio (tg:Ala) were incubated in water at 65 ˚C for seven days and the resulting products were analyzed by hydrophobicity-based separation using C18-HPLC. No products were observed following incubation in water at heated temperature for these monomer mixtures.

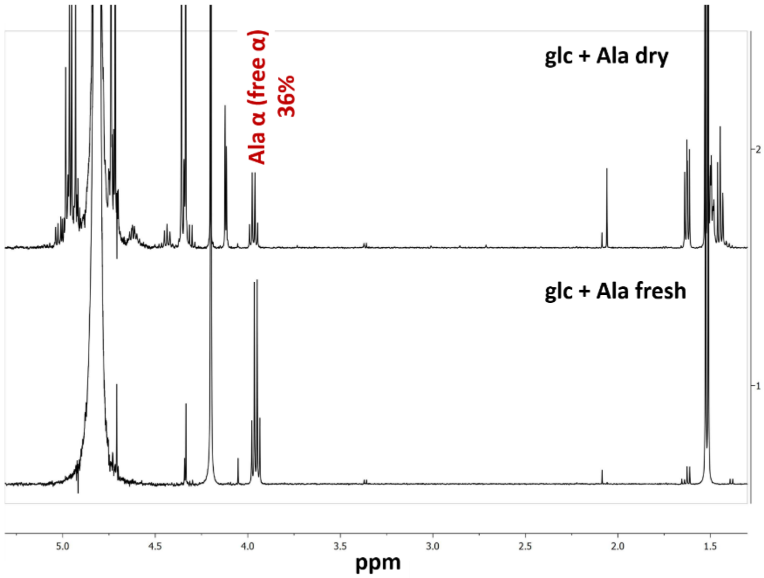

Supplementary Figure 51. Similar conversion of Ala into polymers in dry-down reactions containing mercapto and hydroxy acids. A mixture of glycolic acid (glc) and Ala was dried-down at a 5:1 molar ratio (tg:Ala) at 65 ˚C for seven days. Comparing the ^{1}H NMR of the un-reacted versus reacted mixtures allows one to quantify the overall conversion of Ala monomer into polymers. As seen in the unreacted starting material in a phosphate buffer in D_2O (pH 2.6), the free α-proton chemical shift of Ala is centered at 3.95 ppm. It is evident that 64% of Ala reacted upon dry-down with glc.

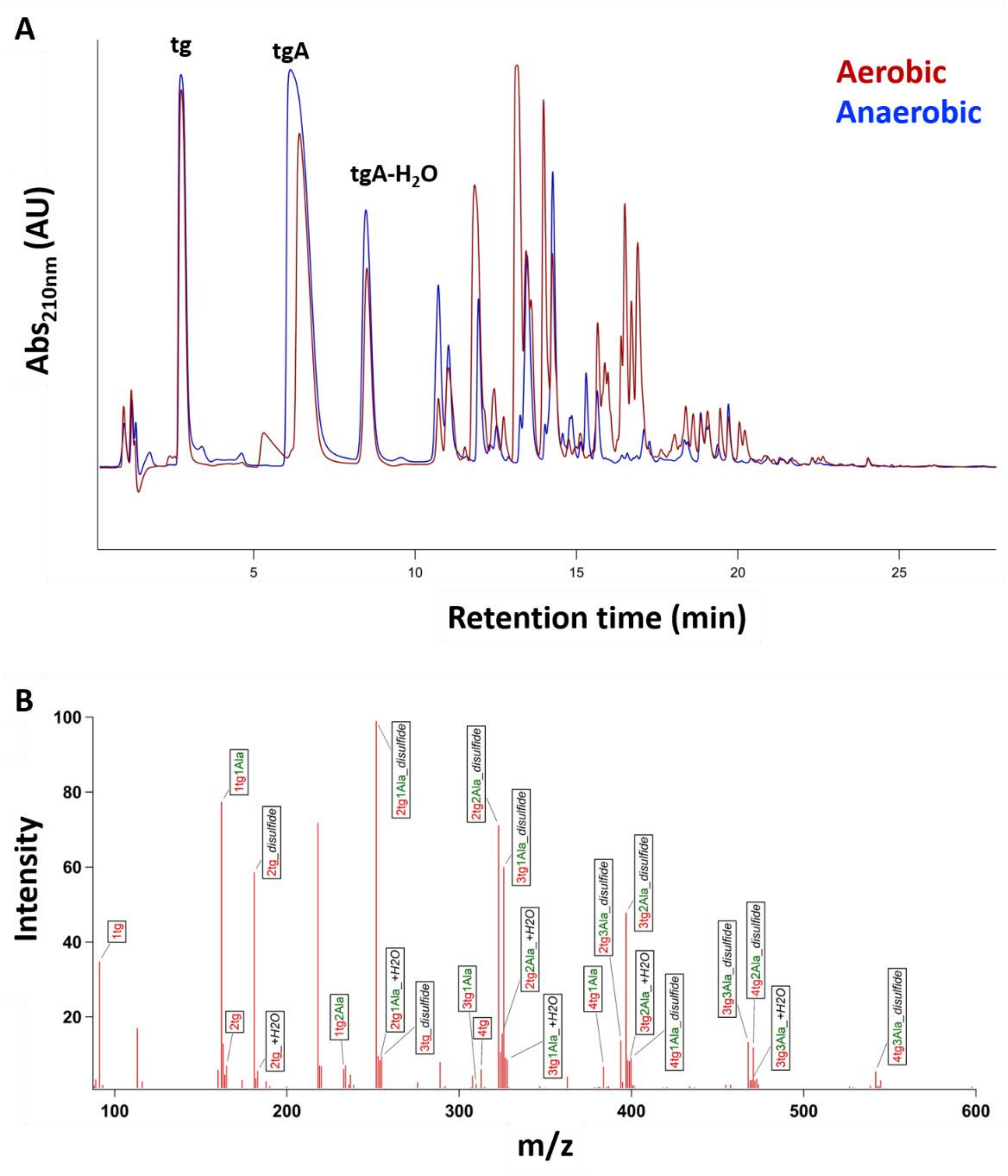

Supplementary Figure 52. Side product formation under oxic conditions. tg and Ala were subjected to continuous dry-down at a 5:1 molar ratio (tg:Ala) at 65 ˚C for up to seven days under either anaerobic or aerobic conditions. Polymer formation is shown by (A) hydrophobicity-based separation using C18-HPLC and by (B) MS. Chromatograms overlaid in A illustrate that more side products are formed under aerobic conditions, supporting the necessity for the usage of an anaerobic chamber.

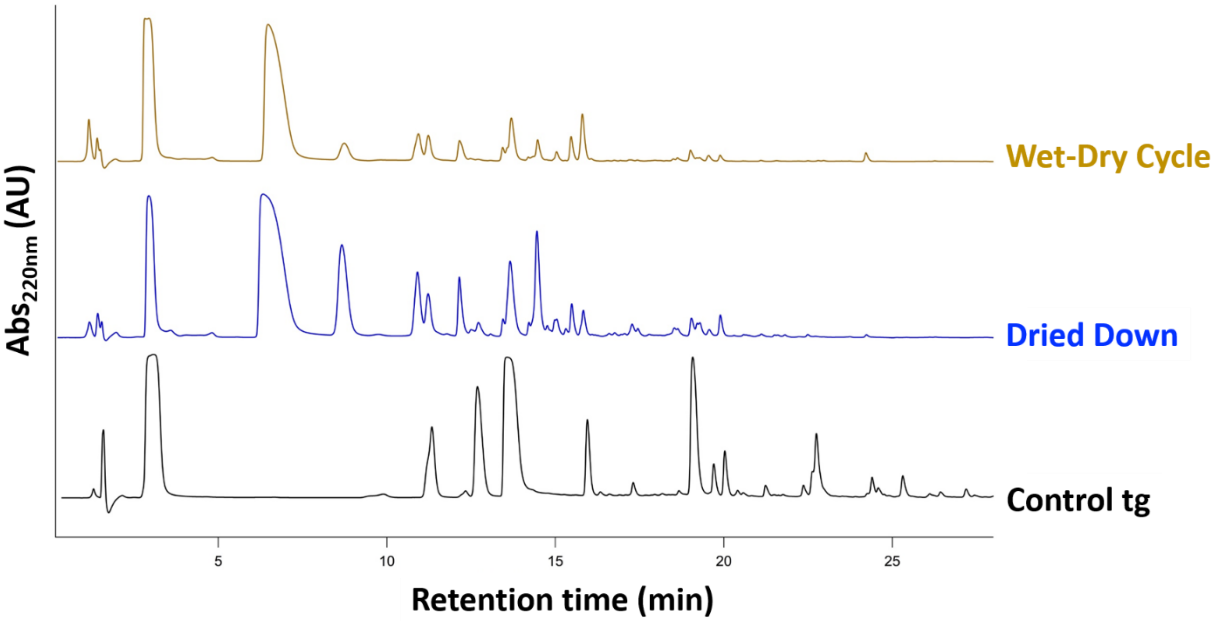

Supplementary Figure 53. Thiodepsipeptide formation during dry-wet cycles. tg and Ala were subjected to daily dry-wet cycles or to continuous dry-down at a 5:1 molar ratio (tg:Ala) at 65 ˚C for up to seven days. Polymer formation is shown via hydrophobicity-based separation using C18-HPLC and indicates that thiodepsipeptides are formed under dry-wet cycles as well, albeit with expected lower yield compared to continuous dry-state reactions.

Supplementary References

1. A. S. Goldsborough *et al.*, Collateral sensitivity of multidrug-resistant cells to the orphan drug tiopronin. *Journal of medicinal chemistry* **54**, 4987-4997 (2011).
2. J. P. McEvoy, Characterizing carbonyls with Infrared spectroscopy: an introductory chemistry experiment in a molecular bioscience program. *J. Chem. Educ.* **91**, 726-729 (2014).
3. A. R. Attar, D. E. Blumling, K. L. Knappenberger Jr, Photodissociation of thioglycolic acid studied by femtosecond time-resolved transient absorption spectroscopy. *The Journal of chemical physics* **134**, 024514 (2011).
4. E. Kim *et al.*, Synthesis of gold nanorod-embedded polymeric nanoparticles by a nanoprecipitation method for use as photothermal agents. *Nanotechnology* **20**, 365602 (2009).
5. K. Tewari, N. Vishnoi, *A textbook of organic chemistry* (Vikas Publishing House, 1976).
