# Supplementary material for: Thioesters provide a plausible prebiotic path to proto-peptides
Source: Nat Commun. 2022 May 11;13:2569. doi: 10.1038/s41467-022-30191-0 (PMC9095695; doi:10.1038/s41467-022-30191-0)
Supplement: Supplementary file 3 — Reporting Summary [file 41467_2022_30191_MOESM3_ESM.pdf]

nature portfolio

Corresponding author(s): Guzman-Martinez & Hud

Last updated by author(s): Apr 4, 2022

Reporting Summary

Nature Portfolio wishes to improve the reproducibility of the work that we publish. This form provides structure for consistency and transparency in reporting. For further information on Nature Portfolio policies, see our Editorial Policies and the Editorial Policy Checklist.

Statistics

For all statistical analyses, confirm that the following items are present in the figure legend, table legend, main text, or Methods section.

n/a	Confirmed	
☒	☐	The exact sample size (n) for each experimental group/condition, given as a discrete number and unit of measurement
☒	☐	A statement on whether measurements were taken from distinct samples or whether the same sample was measured repeatedly
☒	☐	The statistical test(s) used AND whether they are one- or two-sided<br>*Only common tests should be described solely by name; describe more complex techniques in the Methods section.*
☒	☐	A description of all covariates tested
☒	☐	A description of any assumptions or corrections, such as tests of normality and adjustment for multiple comparisons
☒	☐	A full description of the statistical parameters including central tendency (e.g. means) or other basic estimates (e.g. regression coefficient) AND variation (e.g. standard deviation) or associated estimates of uncertainty (e.g. confidence intervals)
☒	☐	For null hypothesis testing, the test statistic (e.g. F, t, r) with confidence intervals, effect sizes, degrees of freedom and P value noted<br>*Give P values as exact values whenever suitable.*
☒	☐	For Bayesian analysis, information on the choice of priors and Markov chain Monte Carlo settings
☒	☐	For hierarchical and complex designs, identification of the appropriate level for tests and full reporting of outcomes
☒	☐	Estimates of effect sizes (e.g. Cohen's d, Pearson's r), indicating how they were calculated

Our web collection on statistics for biologists contains articles on many of the points above.

Software and code

Policy information about availability of computer code

Data collection	ChemStation Edition vC.01.08, TopSpin v4.0.8
Data analysis	Excel, Igor 6.37, MestReNova x64

For manuscripts utilizing custom algorithms or software that are central to the research but not yet described in published literature, software must be made available to editors and reviewers. We strongly encourage code deposition in a community repository (e.g. GitHub). See the Nature Portfolio guidelines for submitting code & software for further information.

Data

Policy information about availability of data

All manuscripts must include a data availability statement. This statement should provide the following information, where applicable:

- Accession codes, unique identifiers, or web links for publicly available datasets
- A description of any restrictions on data availability
- For clinical datasets or third party data, please ensure that the statement adheres to our policy

All the data supporting the findings of this study are available within the main text and its Supplementary Information.

Field-specific reporting

Life sciences study design

All studies must disclose on these points even when the disclosure is negative.

Sample size	No sample size calculation was performed. Experiments were independently repeated from 2-4 times. Sample sizes were chosen based on standard practices to balance the need for ensuring reproducibility while conserving samples and time. The sample numbers used were in all cases sufficient to verify reproducibility of results. Statistical analyses have not been performed.
Data exclusions	Data were not excluded from the paper.
Replication	Experiments were repeated from 2-4 times. Several experiments were repeated independently (at different times and by different individuals) and some dependently (in parallel, by the same individual). All attempts at replication were successful.
Randomization	Randomization is not relevant to our study because all samples were prepared synthetically.
Blinding	Investigators were not blinded to sample identity. Blinding is not relevant to our study because all samples were prepared synthetically.

Reporting for specific materials, systems and methods

We require information from authors about some types of materials, experimental systems and methods used in many studies. Here, indicate whether each material, system or method listed is relevant to your study. If you are not sure if a list item applies to your research, read the appropriate section before selecting a response.

Materials & experimental systems

n/a	Involved in the study	
☒	☐	Antibodies
☒	☐	Eukaryotic cell lines
☒	☐	Palaeontology and archaeology
☒	☐	Animals and other organisms
☒	☐	Human research participants
☒	☐	Clinical data
☒	☐	Dual use research of concern

Methods

n/a	Involved in the study	
☒	☐	ChIP-seq
☒	☐	Flow cytometry
☒	☐	MRI-based neuroimaging
